# Effect of a 72 Hour Stroke Care Bundle on Early Outcomes after Acute Stroke: A Non Randomised Controlled Study

**DOI:** 10.1371/journal.pone.0154333

**Published:** 2016-05-04

**Authors:** Jane Nakibuuka, Martha Sajatovic, Joaniter Nankabirwa, Charles Ssendikadiwa, Nelson Kalema, Arthur Kwizera, Jayne Byakika-Tusiime, Anthony J. Furlan, James Kayima, Edward Ddumba, Elly Katabira

**Affiliations:** 1 Department of Medicine, School of Medicine, Makerere University College of Health Sciences, Kampala, Uganda; 2 Neurological and Behavioral Outcomes Center, University Hospitals Case Medical Center, Cleveland, Ohio, United States of America; 3 Department of Medicine, Mulago National referral hospital, Kampala, Uganda; 4 Department of Anaesthesia and critical care, School of Medicine, Makerere University College of Health Sciences, Kampala, Uganda; 5 Department of Epidemiology and Biostatistics, School of Public Health, Makerere University College of Health Sciences, Kampala, Uganda; 6 University Hospitals Case Medical Center, Neurological Institute, Case Western Reserve University, Cleveland, Ohio, United States of America; 7 Department of Medicine, St Raphael of St Francis Nsambya Hospital, Nkozi University, Kampala, Uganda; University of Glasgow, UNITED KINGDOM

## Abstract

**Background:**

Integrated care pathways (ICP) in stroke management are increasingly being implemented to improve outcomes of acute stroke patients. We evaluated the effect of implementing a 72 hour stroke care bundle on early outcomes among patients admitted within seven days post stroke to the national referral hospital in Uganda.

**Methods:**

In a one year non-randomised controlled study, 127 stroke patients who had ‘usual care’ (control group) were compared to 127 stroke patients who received selected elements from an ICP (intervention group). Patients were consecutively enrolled (controls first, intervention group second) into each group over 5 month periods and followed to 30-days post stroke. Incidence outcomes (mortality and functional ability) were compared using chi square test and adjusted for potential confounders. Kaplan Meier survival estimates and log rank test for comparison were used for time to death analysis for all strokes and by stroke severity categories. Secondary outcomes were in-hospital mortality, median survival time and median length of hospital stay.

**Results:**

Mortality within 7 days was higher in the intervention group compared to controls (RR 13.1, 95% CI 3.3–52.9). There was no difference in 30-day mortality between the two groups (RR 1.2, 95% CI 0.5–2.6). There was better 30-day survival in patients with severe stroke in the intervention group compared to controls (P = 0.018). The median survival time was 30 days (IQR 29–30 days) in the control group and 30 days (IQR 7–30 days) in the intervention group. In the intervention group, 41patients (32.3%) died in hospital compared to 23 (18.1%) in controls (P < 0.001). The median length of hospital stay was 8 days (IQR 5–12 days) in the controls and 4 days (IQR 2–7 days) in the intervention group. There was no difference in functional outcomes between the groups (RR 0.9, 95% CI 0.4–2.2).

**Conclusions:**

While implementing elements of a stroke-focused ICP in a Ugandan national referral hospital appeared to have little overall benefit in mortality and functioning, patients with severe stroke may benefit on selected outcomes. More research is needed to better understand how and when stroke protocols should be implemented in sub-Saharan African settings.

**Trial Registration:**

Pan African Clinical Trials Registry PACTR201510001272347

## Introduction

Across the world, integrated care pathways (ICP) in stroke management are increasingly being implemented to assist health care professionals to manage acute stroke patients in order to achieve pre-specified patient goals efficiently while improving quality of life [1–4]. ICPs appear to be most successful in improving service coordination in the acute stroke context where patient care trajectories are predictable [5]. A review of interventional trials and other studies found that, compared with ‘usual care’ the use of ICPs with stroke was associated with positive and negative impact on patient care, or no impact at all [2, 3, 6]. Patients managed using ICPs for acute stroke reduced the total length of hospital stay and hospital costs, were less likely to be readmitted, or experience pneumonias and urinary tract infections. In addition, they were more likely to have essential and timely diagnostic procedures such as brain computerised tomography (CT) scan, echocardiography and carotid Doppler ultrasound scan. Contrasting reports found that patient satisfaction, quality of life, and probability of good outcomes were significantly lower in the ICP group [2, 6]. One review found that mortality and hospital discharge disposition was similar between patients managed using ICPs and control groups [3]. These findings are not entirely consistent. ICPs for stroke management do contain numerous elements 1) a comprehensive unit with acute and rehabilitation beds; 2) specialist staff (medical, nursing, physiotherapy, occupational, speech and language therapy, social work); 3) multidisciplinary team to ensure good communication, with formal meeting of all staff once per week to plan management of individual patients); 4) education and training with incorporated programmes for staff and provision of information for patients and carers; 5) protocols of care (systematic approach to stroke focused care) that function in a complex and multi-faceted intervention. Stroke focused care includes clinical assessment, routine investigations (biochemistry, hematology, electrocardiography (ECG), CT scan), selected investigations (carotid Doppler ultrasound, echocardiography, magnetic resonance imaging (MRI), angiography), nursing and specialised therapy. Unfortunately, complex ICPs are difficult to implement in resource limited settings. A ‘stroke care bundle’ is a subset of an ICP that contains evidence based care interventions that when combined significantly improve patient outcomes [1, 2, 7, 8]. The Melbourne, Australia 72 hour stroke care bundle [9] contains six elements including a rapid stroke screening for stroke and transient ischemic attack (TIA); brain CT scan in < 24 hours; nil by mouth (NPO) until a bedside swallow evaluation is done in < 24 hours; aspirin if haemorrhagic stroke is excluded within 48 hours; physiological monitoring and management of neurological status, blood glucose, BP, hydration status for 72 hours). The national referral hospital in Kampala, Uganda, like many health centres in sub-Saharan Africa has no stroke unit and thrombolysis, bedside swallow evaluation is not ‘usual care’. Stroke patients are managed on the neurology, neurosurgery units and general intensive care unit (ICU). They may not receive timely clinical and technology based assessment and interventions as well as routine physiological monitoring and management that are important particularly in the acute phase. Thirty day case fatality rate is high 43.8% [10]. The aim of this study was to evaluate the effect of implementing a 72 hour stroke care bundle [9] on early outcomes after acute stroke among patients presenting to the national referral hospital within seven days post stroke.

## Methods

The protocol for this trial and supporting TREND checklist, are available as supporting information (see S1 File and S1 Table respectively). The data base will be made available upon request from the corresponding author.

### Study design

We performed a one year non-randomised controlled study with a quasi-experimental study design. Acute stroke patients were consecutively enrolled (controls first, intervention group second after control group enrolment was complete) into each group over 5 month periods.The control group was managed with ‘usual care’ and the intervention group received a 72-hour stroke care bundle followed by ‘usual care’ thereafter. Patients in both groups were followed to the 30-day from stroke onset. A randomised control study was deemed unethical/impractical in our study setting [11] and thus a sequentially enrolled sample was conducted to provide a control sample and an intervention sample. Given that patients with acute stroke have a number of acute medical problems requiring active intervention during hospital stay, it would be impractical to care for patients on ‘usual care’ and in the intervention group in the same area on the neurology unit. Even with a secluded area for the intervention group, human resource constraints would not permit two different groups of nurses to care for each study group

### Study area and setting

Patients were enrolled in the accident and emergency unit (A&E) at Mulago hospital which has a bed capacity of 1,500. Mulago hospital is located in Kampala the capital city of Uganda with a population of 1,516,210 [12]. The hospital has no stroke unit and thrombolysis and bedside swallow evaluation are not ‘usual care’. The hospital has a radiology department with highly trained specialty personnel. Acute stroke patients (on average 5 patients per week) who present to the A&E unit are housed and managed for a maximum of 24 hours before they are transferred to the neurology unit. Patients that require surgical intervention are transferred to the neurosurgery unit and those that require mechanical ventilation are transferred to the general ICU. The neurology unit which contains on average 50 patients is located on a general medical ward that also includes endocrinology, haematology, and dermatology/ rheumatology units. Collectively, these units provide service to 100 patients on average. Patients are managed by neurologists, internal medicine physicians, medical officers, general nurses, physiotherapists, occupational and speech therapists, social workers, and support staff. Fifteen nurses provide care to the medical ward and patients’ family members assist the nurses in various ways such as turning patients in bed, feeding and bathing them as well as transporting them to the radiology department for investigations.

### Ethical considerations

The study was approved by Makerere University College of Health Sciences’ School of Medicine higher degrees research and ethics committee, Mulago national referral hospital’s research and ethics committee, and The Uganda National Council for Science and Technology. All participants/or family provided written informed consent before participation in the study. All consent forms were approved by the above mentioned research and ethics committees.

The study is in accordance with the Transparent Reporting of Evaluations with Nonrandomised Designs (TREND) statement [13, 14]. Although this trial, aimed to provide preliminary data for a larger study was not registered in an open registry before recruitment of participants started, the authors confirm that all ongoing and related trials for this intervention are now registered under pactr.org. PACTR201510001272347.

### ‘Usual care’ for control group

Patients presenting to the A&E unit with suspected stroke on ‘usual care’ may not receive timely clinical and technology based assessments and interventions as well as routine physiological monitoring and management. Initial stroke screening depends on availability or vigilance of a clinician and brain CT scan may be delayed hours to days. Service limitations are largely due to human resource constraints, insufficient equipment and technology access as well as lack of a formal acute stroke care pathway to standardize clinical care and improve its efficiency and effectiveness [9]. On the neurology unit the ‘Usual care’ for acute stroke is a modification of the American Heart Association /American stroke association (AHA/ASA) guidelines [7, 8]. This includes supportive treatment to ensure a patent airway, adequate oxygen saturation (target >92%), haemodynamic stability, glycaemic control with insulin when serum glucose is >10mmols/L, temperature control with antipyretic paracetamol 1gram if >37.5°C, parenteral normal saline or ringers lactate to maintain adequate hydration in addition to enteral feeding, and measures to prevent pressure sores and deep venous thrombosis with early mobilisation and use of low molecular weight heparin. Antihypertensive drugs (labetalol and hydralazine) are used when BP exceeds 180/105mmHg and 160/100mmHg for ischemic and hemorrhagic strokes respectively. The unit team performs physiologic monitoring and management of neurological status, blood glucose, BP, hydration status (clinical evaluation, fluid input/output), and temperature during the patient’s hospital stay. Among patients with ischemic stroke, antiplatelet drugs including aspirin and clopidogrel, and statins are administered. Rehabilitation includes physiotherapy, occupational, speech and language therapy.

### Stroke care bundle for intervention group

As shown in Table 1 the stroke care bundle aimed to address key deficiencies in ‘usual care’ by providing a formalised acute stroke care pathway to standardize clinical care and improve its efficiency and effectiveness. Funds were availed to ensure timely investigations, sufficient stock of drugs/sundries, equipment such as BP machines, glucometers with gluco-strips, thermometers, pulse oximeters that were required for physiological monitoring and management of acute stroke patients. The assessment tools and protocols for implementing the stroke care bundle elements are available as appendices in the protocol that was provided as supporting information (S1 File). In summary, when a suspected acute stroke patient presented to the A&E, a rapid initial stroke screen was conducted by the study clinicians using the recognition of stroke in the emergency room (ROSIER) scale [15]. The ROSIER assessed the date and time of symptom onset, history of loss of consciousness, seizure activity, level of consciousness using the Glasgow Coma Score (GCS) [16], blood pressure (BP), blood glucose, and hydration status using clinical evaluation. The patient’s temperature was also measured. The study clinicians ensured a patent airway, adequate oxygen saturation (target 92%), temperature control with rectal or parenteral paracetamol if the temperature was >37.5°C, and haemodynamic stability. Patients who presented with blood glucose levels < 3.5 mmol/L were treated with 50mls of 50% dextrose and reassessed when the blood glucose normalised. In addition to the ROSIER assessment, the risk for stroke was also assessed using the ABCD (age, BP, clinical features, duration of symptoms and diabetes) assessment scale [17] among patients that presented in less than 24 hours from stroke onset. Following the rapid initial stroke screen, the patient was then referred for a brain CT scan to confirm stroke. The study clinician prescribed aspirin 300 mg initially and then 150 mg daily if the patient had ischemic stroke on CT scan. Oral medications or fluids were not permitted, until a bedside swallow evaluation [18] was done by a speech and language therapist within 24 hours. Parenteral fluids or drugs could be given before a swallow evaluation was done. Following a swallow evaluation, a patient was categorised as 1) normal feeding, 2) mild swallowing impairment 3) severe swallowing or significant delay in initiation of swallowing. Regular diet, modified diet or blended consistency with thickened fluids and nasal gastric tube insertion for enteral feeding respectively were recommended. Physiological monitoring and management of neurological status, blood glucose, BP, hydration status and temperature were done at 4 to 6 hourly intervals or shorter in case of an abnormal parameter. Management of the abnormal parameters was done as per the study protocols (S1 File). Implementation of the stroke care bundle lasted 72 hours and the patients continued with ‘usual care’ thereafter.

**Table 1:** Elements of the stroke care bundle versus ‘usual care’ for patients presenting to the Accident and Emergency unit with acute stroke.

	Stroke bundle	Usual care
1	Rapid initial stroke screen (ROSIER)	Seen as per availability of a clinician
2	ABCD assessment when TIA is suspected	Not done
3	Brain CT scan in <24 hours	Done as per availability of funds
4	NPO till bed side swallow screen is done in <24 hours	Not done
5	Aspirin within 48 hours if hemorrhagic stroke excluded	Depends on when brain CT scan is done
6	Physiological monitoring and management of Neuro status, blood sugar, blood pressure, hydration status for 72 hours	Not routine

### Training of the study teams

One week prior to commencement of recruitment of patients in the control group, two internal medicine physicians (not part of neurology, and accident and emergency units attending teams) and the principal investigator were trained in participant consenting, administration of the questionnaires, comprehensive clinical assessment, investigations and follow-up procedures.

At the end of the control group recruitment period, a stroke care bundle team comprising two physicians (recruited patients in the control group), one medical officer (looked after control group patients as part of accident and emergency attending team) and fourteen neurology unit nurses (looked after control group patients on the neurology unit) was instructed in protocol bundle implementation and adherence for 3 days (19^th^ July to 21^st^ July). The nurses were trained to do physiological monitoring and management. The head nurse was in charge of the nurses.

### Patient recruitment and study population

We recruited from Monday to Sunday, patients aged 18 years or older, with stroke confirmed on brain CT scan, admitted within 7 days of stroke onset. Patients or proxies provided written informed consent. The preference for a proxy was 1) the spouse, 2) live-in companion, 3) biological child (daughter or son aged 18 years or older, 4) parent, 5) sibling, and lastly 6) a close friend of the patient. We excluded patients who were unconscious or unable to communicate (aphasia, or dementia) with no valid proxy. Stroke was defined according to the criteria of the World Health Organisation (WHO) as sudden onset of focal and at times global neurological deficits, with symptoms lasting more than 24 hours or leading to death, with no apparent cause other than that of vascular origin [19]. Brain CT scan confirmed stroke and classification of stroke subtypes was done using the Trial of ORG 10172 and medical disability guidelines for ischemic and hemorrhagic stroke respectively [20, 21].

### Study procedures

Patients in the control group (details have been described before) [22], and the intervention group had a standardised questionnaire administered by the study clinicians to obtain selected social demographic characteristics including age, sex, tribe, district, religion, level of education, occupation, marital status, date and time of stroke onset, time to hospital presentation from stroke onset, and time of hospital arrival, assessed by an interviewer. Favourable time of hospital arrival was defined in a previous study [23] as daytime presentation during regular hours between 7AM and 5PM Monday to Friday and unfavourable arrival time was any other time including weekends. We also evaluated history of pre-existing stroke risk factors including hypertension, diabetes mellitus, hyperlipidemia, heart disease, prior stroke or TIA, smoking and harmful alcohol consumption. A comprehensive clinical assessment included general and cardiovascular examination, measurement of blood pressure (BP), waist and hip circumferences to determine waist-hip ratio and neurological examination including initial level of consciousness using the Glasgow coma scale (GCS) [16], and severity of stroke using the Scandinavian stroke scale (SSS) [24]. Functional status/disability was assessed using the Barthel Index (BI) [25]. Laboratory investigations included complete blood count (CBC), erythrocyte sedimentation rate (ESR), fasting lipid profile including total cholesterol, low density lipoprotein (LDL), high density lipoprotein (HDL) and triglycerides, fasting blood sugar, HIV test and rapid plasma reagin (RPR) obtained after an eight hour fast. Imaging included brain CT scan, carotid Doppler ultrasound and echocardiography if the cardiovascular examination was abnormal (irregular pulse rate, evidence of valvular pathology, displaced apex beat, presence of a carotid bruit) to look for source of emboli and carotid stenosis. ECG was also done. In addition to the above procedures, patients in the intervention group also had the following: rapid initial stroke assessment and bed side swallow evaluation.

All investigations were done at no cost to the patients in the intervention group. Patients in the control group paid for the brain CT scan.

### Thirty day follow up

Patients in both groups were followed for 30-days post stroke. Discharged patients were scheduled for a neurology outpatient clinic review that coincided with the 30-day follow-up. Prior to the 30-day review date, weekly calls to patients were made to assess general clinical status. BI assessment was done at 30-day follow-up in the neurology clinic. Individuals unable to come to the clinic had the 30-day follow-up conducted in their home. Date of death was obtained for those that died before 30-day follow up.

### Outcome measures

The primary outcome measures were (i) mortality within 3 days from stroke, (ii) mortality within 7 days from stroke, (iii) mortality at 30-days from stroke, and (iv) functional outcome at 30 days as measured by the BI. The BI reflects functional consequences for daily activities that are immediately important to a patient [16] including feeding, dressing, mobility (walking on a level surface and ascending/descending stairs), and personal hygiene (grooming, toileting, bathing, and control of bodily functions). The BI is scored on a total scale of 0–39 (total functional dependence), 40–59 (partially dependent), 60–84 (Independent) and 85–100 (total functional independence). In this study, satisfactory functional outcome was defined as a BI score of ≥ 60. Secondary outcomes included (i) median length of hospital stay, (ii) in-hospital mortality, and (iii) median survival time.

### Calculation of study sample

The study was designed to test the hypothesis that the stroke care bundle would reduce the risk of 30-day mortality compared to ‘usual care’ in patients presenting with acute stroke at Mulago hospital. Using the PASS 2008 software for two independent proportions (null case) power analysis [26–28], and estimating the risk of 30-day mortality among adult stroke patients admitted to Mulago hospital at 43.8% [10], we would need to enrol 127 patients in the control group and 127 patients in the intervention group to observe a difference of 15% or more, setting the power at 80% and significance at 0.05.

### Data analysis

Univariate analysis was used to describe the social demographic, clinical and laboratory characteristics of the study patients. The primary outcomes at 30-days from stroke onset were death defined as the proportion of stroke events that were fatal within 30-days of stroke onset and satisfactory outcome defined as a BI score of ≥60. Secondary outcomes were in-hospital mortality, median survival time, and median length of hospital stay. Time at risk of death was calculated from time of stroke onset to time of death or censor and time at risk of a satisfactory outcome was calculated from time of stroke onset to time of the outcome/censor. Incidence outcomes at 7 days and 30 days were compared using a chi square test and adjusted for potential confounders namely gender, level of education, stroke severity at admission, fasting blood sugar, total cholesterol, LDL, HDL, time to hospital presentation from stroke onset. Measures of association were expressed as risk ratios and a p value ≤0.05 was considered statistically significant. Kaplan Meier survival estimates and log rank test for comparison were used for time to death analysis for all strokes and by stroke severity categories. A significant P value from the log rank test was set at ≤.05. Data were analysed using Stata version 12.0 software (StataCorp, College Station, Texas).

## Results

### Characteristics of the study participants

**Fig 1:**
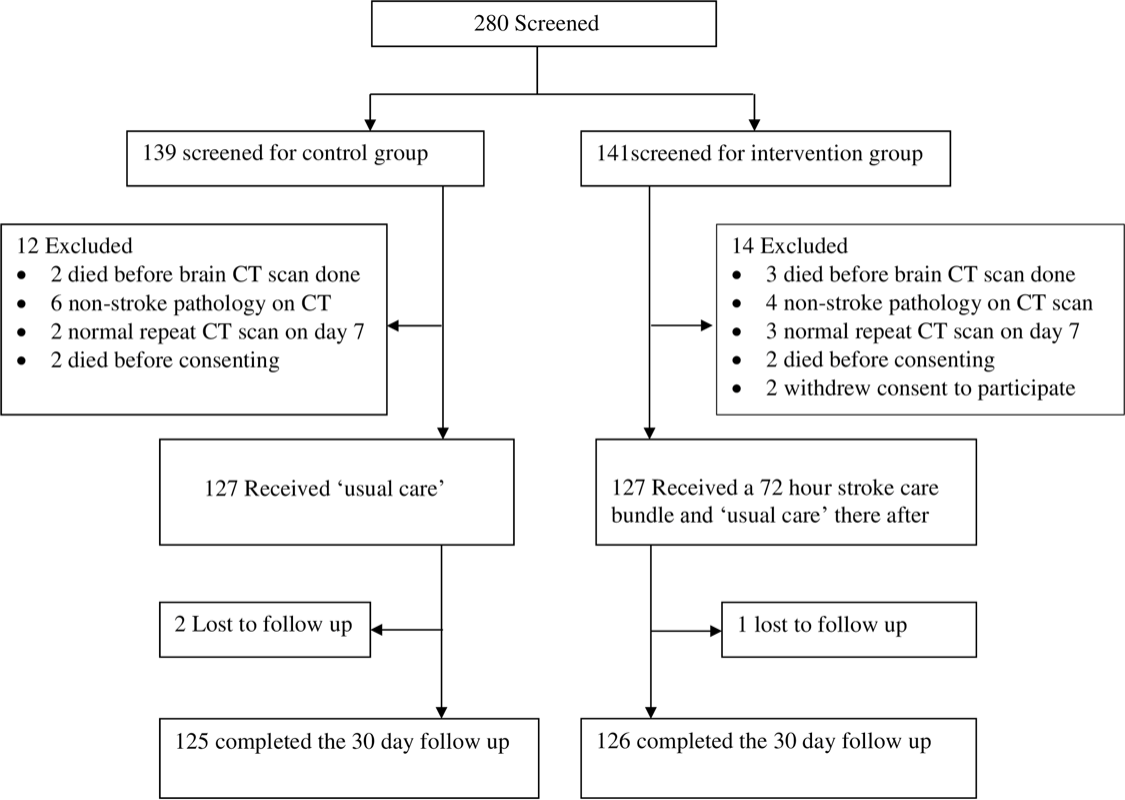
Consort flow diagram.

During the period 15^th^ February 2014 to 19^th^ July 2014, 127 acute stroke patients were consecutively recruited into the control group out of 139 patients that presented to the A&E unit with neurological deficits suggestive of acute stroke. As illustrated in (Fig 1), we excluded 12 patients, 2 patients were lost to follow up, and 125 patients completed the 30-day follow up.

In the period 21^st^ July to 28^th^ December 2014, 127 acute stroke patients were consecutively enrolled into the intervention group out of 141 patients that presented to the A&E unit with neurological deficits suggestive of acute stroke within 7 days. As illustrated in (Fig 1), we excluded 14 patients, one patient was lost to follow up, and 126 patients completed the 30-day follow up.

Table 2 and Table 3 compare the socio-demographics, clinical and laboratory characteristics of the study patients in the control and intervention groups. At admission, the two groups significantly differed in level of education, time to hospital presentation post stroke, stroke severity, and a number of laboratory characteristics including fasting blood sugar, total cholesterol, LDL, HDL and RPR.

**Table 2:** Social demographic characteristics of the study patients.

Variable	Control group (N = 127), n (%)	Intervention group (N = 127), n (%)	P value	Overall P-value
**Sex**				
Male	59 (46.5)	44 (34.7)		
Female	68 (53.5)	83 (65.3)	0.056	
**Age group (years)**				0.636
<51	38 (29.9)	33 (26.0)	0.489	
51–60	26 (20.5)	31 (24.4)	0.456	
61–70	26 (20.5)	22 (17.3)	0.515	
≥71	37 (29.1)	41 (32.3)	0.580	
**Highest level of Education**				
None + Primary	71 (55.9)	90 (70.9)		
Secondary + Tertiary	56 (44.1)	37 (29.1)	**0.013**	
**Marital status**				
Not married	61 (48.0)	66 (51.9)		
Married	66 (51.9)	61 (48.0)	0.534	
**Employment level**				0.440
Un-employed	69 (54.3)	71 (55.9)	0.798	
Casual employment	44 (34.7)	48 (37.8)	0.607	
Professional employment	14 (11.0)	8 (6.3)	0.183	
**Known hypertensive**				
No	49 (38.6)	55 (43.3)		
Yes	78 (61.4)	72 (567)	0.446	
**Known diabetes mellitus**				
No	115 (90.6)	117 (92.1)		
Yes	12 (9.4)	10 (7.9)	0.671	
**Known heart disease**				
No	119 (93.7)	114 (89.8)		
Yes	8 (6.3)	13 (10.2)	0.259	
**Prior stroke /TIA**				
No	37 (29.1)	30 (23.6)		
Yes	90 (70.9)	97 (76.4)	0.320	
**Smoking**				
No	120 (94.5)	118 (92.9)		
Yes	7 (5.5)	9 (7.1)	0.600	
**Alcohol consumption**				
No	84 (66.1)	93 (73.2)		
Yes	43 (33.9)	34 (26.8)	0.218	
**Elevated waist-hip ratio**				
No	38 (29.9)	28 (22.1)		
Yes	89 (70.1)	99 (77.9)	0.156	

TIA Transient Ischemic Attack.

**Table 3:** Clinical and laboratory characteristics of the study patients.

Variable	Control group (N = 127), n (%)	Intervention group (N = 127), n (%)	P value	Overall P-value
**Time to presentation from stroke onset**				**0.015**
Within 24 hours	38 (29.9)	26 (20.5)	0.085	
24–48 hours	26 (20.5)	22 (17.3)	0.515	
> 48 hours to 7 days	63 (49.6)	79 (62.2)	0.043	
**Time of arrival**				
Favorable	67 (52.8)	73 (57.5)		
Unfavorable	60 (47.2)	54 (42.5)	0.449	
**Stroke subtype**				
Hemorrhagic	39 (30.7)	52 (40.9)		
Ischemic	88 (69.3)	75 (59.1)	0.090	
**Temperature**				
Afebrile (temp < 37.5°C)	118 (92.9)	112 (88.2)		
Febrile (temp ≥ 37.5°C)	9 (7.1)	15 (11.8)	0.200	
**Admission blood pressure (BP)**				
BP < 140/90mmHg	30 (23.6)	26 (20.5)		
BP ≥ 140/90mmHg	97 (76.4)	101 (79.5)	0.551	
**Level of consciousness at admission**				
GCS ≥ 9	111 (87.4)	106 (83.5)		
GCS < 9	16 (12.6)	21 (16.5)	0.378	
**Stroke severity at admission**				**0.002**
Mild (45–58)	20 (15.7)	8 (6.3)	0.017	
Moderate (30–44)	25 (19.7)	14 (11.0)	0.054	
Severe (15–29)	43 (33.9)	51 (40.2)	0.299	
Very severe (0–14)	39 (30.7)	54 (42.5)	0.051	
**Disability at admission**				0.406
Independent	18 (14.2)	10 (7.9)	0.109	
Partially dependent	19 (14.9)	26 (20.5)	0.242	
Totally dependent	90 (70.9)	91 (71.6)	0.902	
**Fasting blood sugar**				**0.013**
Normal	65 (51.2)	46 (36.2)	0.016	
IGT	25 (20.5)	29 (22.8)	0.656	
High	36 (28.3)	52 (40.9)	0.035	
**Total cholesterol**				**0.002**
Normal	41 (32.3)	56 (44.1)	0.053	
Borderline	20 (15.8)	35 (27.6)	0.023	
High	66 (51.9)	36 (28.3)	<0.001	
**LDL cholesterol**				**0.001**
Normal	70 (55.1)	94 (74.0)	0.002	
Borderline	16 (12.6)	12 (9.5)	0.415	
High	41 (32.3)	21 (16.5)	0.003	
**HDL cholesterol**				**0.001**
Normal	38 (29.9)	68 (53.5)	<0.0001	
Borderline	59 (46.5)	53 (41.7)	0.441	
High	30 (23.6)	6 (4.7)	<0.0001	
**Triglycerides**				0.756
Normal	101 (79.5)	103 (81.1)	0.749	
Borderline	15 (11.8)	14 (11.0)	0.841	
High	11 (8.7)	10 (7.9)	0.817	
**Rapid plasma reagin**				
Nonreactive RPR	118 (92.9)	127 (100)		
Reactive RPR	9 (7.1)	0 (0.00)	**0.002**	
**HIV**				
Negative	121 (95.3)	124 (97.6)		
Positive	6 (2.7)	3 (2.4)	0.322	
**WBC count**				0.053
Low	13 (10.2)	6 (4.7)	0.095	
Normal	97 (76.4)	96 (75.6)	0.881	
High	17 (13.4)	25 (19.7)	0.177	

IGT Impaired glucose tolerance, LDL low density lipoprotein, HDL high density lipoprotein, RPR rapid plasma reagin, WBC white blood cell, GCS Glasgow coma score.

### Fidelity of intervention delivery

The stroke care bundle contains six elements. 1) The study clinician was readily available to do a rapid initial stroke screen within 15 minutes from patient presentation to the accident and emergency unit; 2) the ABCDD assessment of patients who presented within 24 hours of symptom onset was done concurrently with the initial stroke screening; 3) Of 127 patients recruited during the intervention period, 11 (8.7%), presented to hospital with CT scan of the brain already done at another hospital before they were referred to Mulago hospital. The median time to CT scan for patients in the intervention group was 2 hours (IQR 0.5–12) as compared to the control group 34.5 hours (IQR 24–60) (p = <0.001); 4) Swallow evaluation is not ‘usual care’ and hence none of the control group patients were assessed for swallowing ability. All the 127 patients in the intervention group were evaluated for pre-feeding requirements for a swallow evaluation. Fifty six (44.1%) patients met the pre-feeding requirements and all of them had a bedside swallow evaluation performed within 24 hours of admission; 5) Aspirin was promptly prescribed for all the 75 patients in the intervention group, in whom haemorrhagic stroke was excluded on CT scan; 6) monitoring and management of neurological status, BP, blood glucose, temperature, hydration status was complete for the first 72 hours of admission in 87 patients out of 127 patients (16 died within 72 hours and 24 were discharged before the 72 hours elapsed. Fifteen patients out of 127 had a high temperature on admission and normalisation of the temperature was achieved within 24 hours.

### Outcome measures

Only one person died within 3 days in the control group compared to 16 in the intervention group with the incidence of 3-day mortality of 0.7% (95% CI 0.1–4.3%) in the control group and 12.6% (95% CI 7.9%–19.5%) in the intervention group. Table 4 shows primary outcomes at 7 days and 30 days. Mortality was higher in the intervention group compared to controls at 7-days (RR 13.1, 95% CI 3.3–52.9). There was no significant difference in 30-day mortality (RR 1.2, 95% CI 0.5–2.6) and functional outcome at 30-days (RR 0.9, 95% CI 0.4–2.2) between the control and intervention groups. At 30 days from stroke onset, two patients were lost to follow up in the control group and one in the intervention group. There were 77 (60.6%) survivors in the intervention group and out of these, 35 (45.5%) had satisfactory outcome (22 totally independent and 13 independent). There were 91 (71.7%) survivors in the control group and out of these, 49 (53.8%) had satisfactory outcome (30 totally independent and 19 independent).

**Table 4:** Mortality and satisfactory outcome by treatment group.

Outcome	Control group, 127 patients		Interventional group, 127 patients		Unadjusted RR 95% CI	p-value	Adjusted RR	p-value
	Events	Incidence (%) 95% CI	Events	Incidence (%) 95% CI				
**Mortality**								
Within 7 days	8	6.3 (0.3–11.9)	33	25.9 (19. 1–34.2)	5.2 (2.5–14.2)	<0.001	13.1 (3.3–52.9)	**<0.001**
Within 30 days	34	26.7 (19.8–35.1)	49	38.6 (30.6–47.3)	1.7 (1.01–2.9)	0.046	1.2 (0.5–2.6)	0.676
**Satisfactory outcome**	49	38.6 (30.6–47.3)	35	27.6 (20.5–56.0)	0.6 (0.4–1.03)	0.063	0.9 (0.4–2.2)	0.874

Adjusted for age-group, sex, educational level, stroke severity, fasting blood sugar, total cholesterol, LDL cholesterol, HDL cholesterol, time to hospital presentation, Satisfactory outcome also adjusted for disability at admission.

Regarding secondary outcomes, in-hospital mortality was 41 (32.2%) in intervention group and 23 (18.1%) in the control group with absolute change -14.1(95% CI-24.5–-3.5); P<0.001. The median survival time was 30 days (IQR 29–30 days) in the control group and 30 days (IQR 7–30 days) in the intervention group. The median length of stay was 8 days (IQR 5–12 days) in controls and 4 days (IQR 2–7 days) in the intervention group. Among patients that did not die in hospital, the median length of hospital stay was 8 days (IQR 5–12 days) in controls and 5 days (IQR 3–8 days) in the intervention group.

**Fig 2:**
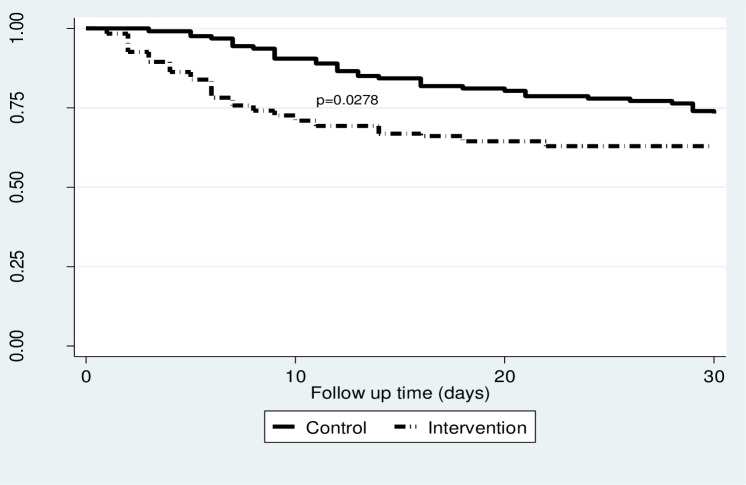
shows Kaplan Meier survival curves comparing survival of all participants in the control group and intervention group.

**Fig 3:**
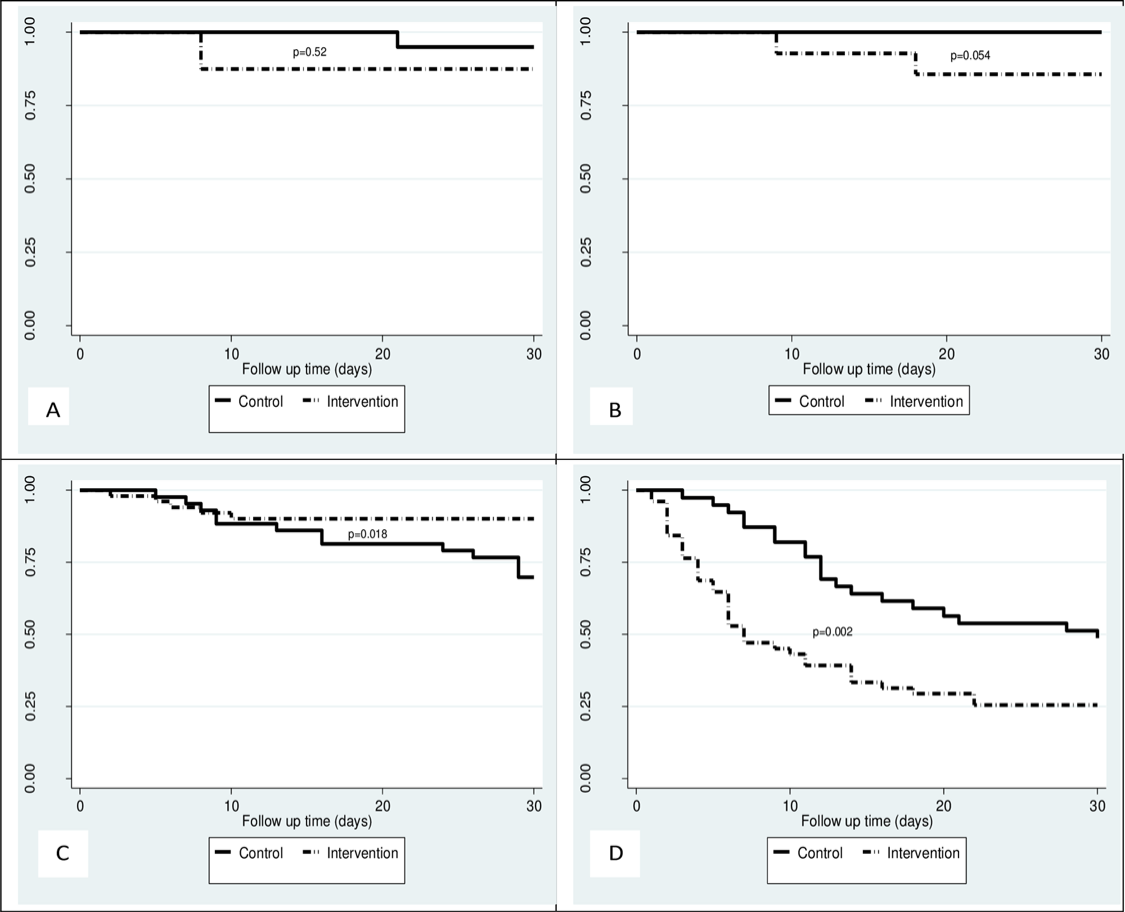
Kaplan Meier survival curves by stroke severity category comparing survival of patients in the control group and the intervention group. (A) shows Kaplan Meier curves comparing survival of participants with mild stroke in the control and intervention groups. (B) shows Kaplan Meier curves comparing survival of participants with moderate stroke in the control and intervention groups. (C) shows Kaplan Meier curves comparing survival of participants with severe stroke in the control and intervention groups. (D) shows Kaplan Meier curves comparing survival of participants with very severe stroke in the control and intervention groups. The p values were obtained from the log rank tests.

As illustrated in (Fig 2), the Kaplan-Meier survival curves comparing survival of patients in the control and intervention groups showed significant difference between the groups for 30-day mortality (log rank test P = 0.0278) in favour of the control group. When stratified by category of stroke severity, no significant difference was found between the groups for the mild stroke category (p = 0.52) as shown in (Fig 3A), although the moderate stroke category showed somewhat difference in survival in favour of the controls (p = 0.054) (Fig 3B). Among patients in the severe stroke category, there was better survival of patients in the intervention group compared to controls (p = 0.018) (Fig 3C) although in the very severe stroke category, there was better survival of patients in the controls compared to intervention group (p = 0.002) (Fig 3D).

## Discussion

In this study, we demonstrated fidelity in implementing the stroke care bundle in a resource limited setting. The processes of care were consistently performed with a 17 fold reduction in time to CT scan.

In spite of the 72 hour stroke focused care, 7-day mortality was higher in the intervention group compared to the controls, and there was no significant difference in 30-day mortality and functional outcome between groups. This is consistent with findings in a Cochrane review [2, 29] with some studies reporting a significant negative effect of ICPs on outcomes [6]. The intervention group generally had more patients with severe stroke, including hemorrhagic strokes and a history of prior strokes, heart disease, abdominal obesity, hyperglycemia, high lipid profiles, delayed admissions that were less educated, and female. Even with standard methods of case-mix adjustment, these differences may have impacted our ability to determine the effectiveness of the stroke care bundle on outcomes. Stroke severity could have adversely contributed to poor outcomes beyond the ability of any interventional care protocol to impact outcome. Severe stroke is an independent predictor of early mortality from stroke from multiple studies [22, 30, 31] and it is directly related to the severity of the neurological damage [32, 33]. In our study, we are uncertain why more severe stroke patients presented to hospital during the intervention study period (the last 6 months of the year). Severe strokes have been associated with seasonal variation [34, 35]. In contrast with our study, implementing an ICP in a cluster randomised trial of ischemic stroke patients significantly lowered risk of 7-day mortality [29]. Similarly, a study in a resource limited setting reported a reduction of in-hospital mortality from 33% to 16% after implementing an ICP [36]. In these studies, patients were admitted to stroke units that had stroke focused care for their entire hospitalization in contrast to our study which had stroke focused care for 72 hours. Admission to stroke units has been reported to improve outcomes [37–39].

Findings in this preliminary study suggest that the stroke care bundle might reduce mortality in some patients with severe strokes. Patients with severe stroke in the intervention group were significantly more likely to be alive at 30 days post stroke compared to patients with severe stroke in the control group. It is possible that the more aggressively implemented care (as represented by the stroke care bundle) is most helpful for individuals with a great amount of impairment and highest risk of complications. Additional research is needed to more conclusively determine if there may be optimal patient populations on which to focus ICPs.

Hospital stay was significantly shorter in the intervention group. This was in agreement with multiple studies [2] but in disagreement with other studies that reported an unclear impact of ICPs on hospital stay [4]. In our study, all intervention patients spent fewer days in hospital. One might argue that this was so because of a higher mortality in this group compared to the controls but likewise, survivors in the intervention group spent less days in hospital compared to controls. Possibly, early CT scan implementation favoured early discharge in less severe strokes or timely aggressive treatment of more severe strokes. Reduced length of hospital stay invariably translates in to reduced hospital costs and it will be important for resource limited settings to study cost effects of ICPs in future work.

Facilitators to implementation of the intervention included adequate funding which ensured readily available resources for stroke focused care. The stroke care protocol streamlined patient flow from the accident and emergency unit to the neurology, neurosurgery units and the general ICU. Training of nurses and explaining the rationale for implementation of the care pathway made them more knowledgeable about acute stroke care guidelines, clearly defined the roles of doctors and nurses, and enhanced their involvement in patient care. Weekly meetings and senior team supervision ensured continued adherence to the study protocols. The main barrier to implementation of the stroke care pathway was the impression of appearing overly prescriptive and additional to the pre-existing burden of care on an already over-stretched nursing staff. An ICU bed was not always readily available and so some admissions were delayed.

There are limitations of our study. The non-randomised design may not have been able to account for temporal differences that could have occurred at our facility during the study time frame or even guarantee complete removal of bias, despite standard methods of case-mix adjustment. The stroke care bundle provided specific stroke–focused care for 72 hours only and this could have been an example of “too little, too late” especially for those with delayed hospital admission to begin with. There could also have been differences in informal supports available to the patients in the intervention and control groups such as care delivered by family. The sample size was small given the wide confidence intervals.

## Conclusions

This preliminary study in Uganda using a stroke care bundle did not find improved mortality or functional outcomes, although there were improved outcomes in some stroke patient sub-groups. Given the fact that stroke burden in the resource limited settings is expected to increase, it will be important to further develop and test ICPs that can facilitate delivery of stroke care that is efficient, practical, and may be able to generally improve patient outcomes. However, interventions to prevent severe strokes should be studied and emphasized as a preferred way to reduce stroke burden.

## Supporting Information

S1 File Study Protocol. (PDF)

S1 Table TREND checklist. (PDF)
