# Supplementary material for: Effect of a 72 Hour Stroke Care Bundle on Early Outcomes after Acute Stroke: A Non Randomised Controlled Study
Source: PLoS One. 2016 May 4;11(5):e0154333. doi: 10.1371/journal.pone.0154333 (PMC4856379; doi:10.1371/journal.pone.0154333)
Supplement: S1 File — (PDF) [file pone.0154333.s001.pdf]

IMPROVING STROKE PREVENTION AND OUTCOMES IN UGANDA: POPULATION SURVEY AND HOSPITAL BASED STUDY

Researcher: Dr. Nakibuuka Jane MBChB (MAK), MMED (Internal Med, MAK)
Registration No: 2011/HD07/4990U

Supervisors

Prof. Elly Katabira, FRCP (School of Medicine, MaKCHS)
Prof. Anthony Furlan, PhD (Case Western Reserve University)
Dr. Jayne Byakika Tusime, PhD (School of Public Health, MakCHS)
Dr. Edward Ddumba, MBBS, MMED, Dip Neuro (Nkozi University)

Proposal submitted in fulfilment of the requirement for the award of Doctor of Philosophy of Makerere University Kampala, Uganda

Version 2

Table of Contents

LIST OF FIGURES v

LIST OF TABLES vi

LIST OF ABBREVIATIONS vii

OPERATIONAL DEFINITIONS ix

ABSTRACT xi

1.0 INTRODUCTION 1

CHAPTER TWO 3

2.0 BACKGROUND 3

2.1 Burden of stroke in the world 3

2.2 Burden of stroke in Africa 3

2.3 Burden of stroke in Uganda 3

2.4 Causes of stroke and subtypes 4

2.4.1 Ischemic stroke 4

2.4.2 Hemorrhagic stroke 5

2.5 Causes of mortality from stroke 5

2.5.1 Complications of stroke 6

2.6 Acute stroke management 8

2.7 Risk factors of stroke 9

2.7.1Traditional risk factors associated with stroke 9

2.7.1.1 Modifiable/ controllable risk factors 9

2.7.1.2 Non-modifiable/uncontrollable stroke risk factors 14

2.7.2 Emerging Risk Factors 15

2.8 Prevention of stroke 18

2.8.1 Perceptions of stroke 19

2.8.2 Knowledge of stroke 21

2.8.3 Sources of information 23

CHAPTER THREE 24

3.0 STATEMENT OF THE PROBLEM 24

CHAPTER FOUR 25

4.0 RESEARCH OBJECTIVES 25

4.1 RESEARCH HYPOTHESIS 25

4.2 Justification of the study 26

4.3 Significance of the study 27

4.4 Figure 1: Conceptual Framework 28

CHAPTER FIVE 29

5.0 METHODOLOGY 29

5A.0 METHODOLOGY FOR AIM I 29

5A.1 Study area and population 29

5A.1.1 Study design 31

5A.1.2 Study population 31

5A.1.3 Selection criteria 31

5A.1.4 Sample size calculation 31

5A.1.5 Sampling procedure 32

5A.1.7 Data collection tools 35

5A.1.9 Data analysis 39

5B METHODOLOGY FOR AIM II 41

5B.1 Study design 41

5B.2 Study site 41

5B.3 Study population 41

5B.4 Selection criteria 41

5B.5 Sample size calculation 42

5B.6 Sampling method and recruitment 43

5B.8 Thirty day follow up 49

5B.9 Data collection 53

5B.9.1 Data collection tools 53

5B.9.2 Data collection method 54

5B.9.3 Adverse events reporting 55

CHAPTER SIX 60

6.0 PROTECTION OF HUMAN SUBJECTS 60

6.1 Institutional Review Boards 60

6.2 Informed consent 60

6.3 Risks and Discomforts 60

6.5 Study limitations 61

References 62

APPENDIX 1: STROKE AND TRANSIENT ISCHEMIC ATTACK (TIA) CARE BUNDLE [73] 70

APPENDIX 2: AUDIT FORM 71

APPENDIX 3: ROSIER SCALE 72

APPENDIX 4: GLASGOW COMA SCALE 73

APPENDIX 5: ABCD2 ASSESSMENT WHEN TIA SUSPECTED [165] 74

APPENDIX 6: DYSPHAGIA SCREENING TOOL 75

APPENDIX 7(a): PHYSIOLOGIC MONITORING 77

APPENDIX 7(b): PHYSIOLOGICAL MONITORING 24 HOUR CHART 82

APPENDIX 8: QUESTIONNAIRE (HOSPITAL STUDY-PRE-INTERVENTION) 83

APPENDIX 9 (a): CONSENT FORM (HOSPITAL STUDY PRE-INTERVENTION) 94

APPENDIX 9 (b): CONSENT FORM FOR IN-HOSPITAL STUDY (PRE-INTERVENTION) IN LUGANDA 97

APPENDIX 10: QUESTIONNAIRE (HOSPITAL STUDY –INTERVENTION) 100

APPENDIX 11 (a): CONSENT FORM (HOSPITAL STUDY-INTERVENTION) 111

APPENDIX 11 (b): CONSENT FORM FOR HOSPITAL STUDY (INTERVENTION STAGE) IN LUGANDA 114

APPENDIX 12: SURVEY QUESTIONNAIRE 117

APPENDIX 13 (a): CONSENT FORM FOR SURVEY 125

APPENDIX 13 (b): CONSENT FORM FOR SURVEY IN LUGANDA 127

APPENDIX 14: THE INTERNATIONAL PHYSICAL ACTIVITY (IPA) SCORE[156] 130

APPENDIX 15: CAGE ALCOHOL DEPENDENCY GRADING SYSTEM [157] 131

APPENDIX 16: DIAGNOSTIC CRITERIA FOR CEREBRAL INFARCTION 132

APPENDIX 17: PROCEDURE FOR MEASURING BLOOD PRESSURE 133

APPENDIX 18: NATIONAL CLINICAL GUIDELINE 127 [158] 134

APPENDIX 19: PROCEDURES FOR ARTHROPOMETRIC MEASUREMENTS 135

APPENDIX 20: SCANDINAVIAN STROKE SCALE [159] 137

APPENDIX 21: MODIFIED RANKIN SCALE [162] 139

APPENDIX 22: THE BARTHEL INDEX [163] 140

APPENDIX 23: ADVERSE EVENT REPORTING FORM [170] 142

APPENDIX 24: ACTIVITY PLAN .... **Error! Bookmark not defined.**

APPENDIX 25: BUDGET .... **Error! Bookmark not defined.**

LIST OF FIGURES

Figure 1, conceptual framework 28

Figure 2, showing the location of Wakiso district on the map of Uganda…………………...29

Figure 3, showing the map of Wakiso district and the location of the selected study areas...30

Figure 4, showing sampling and operational flow diagram for the survey ………….........34

Figure 5, showing sampling and operational flow diagram for study aim 1

(Pre-intervention) study...51

Figure 6, showing sampling and operational flow diagram for study aim 1

(Intervention) study……………………………………………………………..52

Figure 7, demonstrating the Frankfort plane……………………………………………….135

LIST OF TABLES

Table 1, summary of prevalence of stroke associated risk factors in Uganda studies………17

Table 2, showing the characteristics of the selected sub-counties of Wakiso district………31

Table 3, showing the survey's required sample size...32

LIST OF ABBREVIATIONS

AE	Adverse event
AF	Atrial fibrillation
ABCD^2	Age, Blood Pressure, Duration, Diabetes
ADL	Activities of Daily Living
AHA	American heart Association
AIDS	Acquired Immune Deficiency Syndrome
ASA	American Stroke Association
BMI	Body mass index
CBC	Complete Blood Count
CI	Confidence Interval
CT	Computerised tomography
CVA	Cerebral Vascular Accident
CVD	Cerebral vascular disease
DM	Diabetes Mellitus
ECG	Electrocardiography
ESR	Erythrocyte Sedimentation Rate
GCS	Glasgow Coma Scale
HDLc	High density lipoprotein cholesterol
HIV	Human Immunodeficiency Virus
HT	Hypertension
ICH	Intracerebral hemorrhage
IRB	Institutional Review Board
LDLc	Low density lipoprotein cholesterol
MaKCHS	Makerere university College of Health Sciences
MAP	Mean Arterial Pressure
MRI	Magnetic Resonance Imaging
MEPI-CVD	Medical Education Partnership Initiative-Cardiovascular Disease
OR	Odds Ratio
PAR	Population Attributable Risk
PI	Principal Investigator
RCT	Randomised Controlled Trial
ROSIER	Recognition of Stroke In the Emergency Room

SAE	Serious adverse event
SAH	Subarachnoid hemorrhage
SSA	Sub-Saharan Africa
TB	Tuberculosis
TGs	Triglycerides
TIA	Transient ischemic stroke
TOAST	Trial of Org 10172 in Acute Stroke Treatment
UK	United Kingdom
USA	United States of America
WHO	World Health Organisation

OPERATIONAL DEFINITIONS

Stroke: According to the WHO definition, a stroke (cerebrovascular disease) event is defined as rapidly developing signs of focal (global) disturbance of cerebral function of presumed vascular origin leading to death or lasting more than 24 hours (unless interrupted by surgery). This definition includes all patients presenting with clinical signs and symptoms of subarachnoid hemorrhage, intracerebral hemorrhage, thrombosis and embolism but will exclude patients presenting with transient ischemic attacks (TIA) with recovery of the neurological deficit within 24 hours, subdural and extradural haematomas, brain tumours, head injuries.

Current smoker is defined as a participant who smoked at least one cigarette per day over the past 3 months or less.

Current medication: Drugs that the patient is taking during the past three months to present

Household: Is defined as any single permanent or semi-permanent dwelling structure acting as the primary residence for a person or group of people that generally cook and eat together. Some households may include members who sleep in other dwelling structures within the same compound, but cook and eat together and are still dependent on the head of household in the main household.

Head of household: An adult member of the household who primarily makes decisions for the rest of the members, regarding health care, income, food, education, etc.

Household resident: A person who intends to have a sleeping place in a household for the next six months. This includes people who sleep in another house within the same compound, if they still depend on the head of the household for decisions on finances, health care, and share meals together.

Health transition: A term of art referring to the change in the disease 'mix' of a population as it undergoes westernisation which, in general, is marked by an increased lifespan and reduction in death due to infection and an increase in cancer, diabetes and cardiovascular and stroke mortality.

Demographic transition: A shift in a population from an old demographic situation marked by natality and mortality that are high and that approximately balance out, to a modern demographic situation in which natality and mortality are low and also approximately balance out. During the transition, mortality is lower than natality and the population grows fast.

Oropharyngeal transit time: The time from the introduction of the water bolus to the oral cavity, to the point of initiation of the pharyngeal swallow.

30 day mortality will be defined as death occurring within 30-35 days from the onset of the stroke (also referred to as 30 day case fatality).

Adverse event: Any untoward medical occurrence in a participant administered a pharmaceutical product that does not necessarily have a causal relationship with this treatment. Examples include but are not limited to:

1. Worsening of conditions present after the administration of medicine
2. Inter-current illness
3. Events related or possibly related to concomitant medications
4. Hypersensitivity to drugs- paracetamol, labetalol, regular insulin
5. Any other significant complication

Serious adverse event: An experience that results in any of the following outcomes

1. Death during the period of study follow-up
2. Life threatening experience (one that puts a participant at immediate risk of death at the time of the event)
3. Inpatient hospitalisation during the period of study follow –up
4. Persistent or significant disability or incapacity
5. Specific medical or surgical intervention to prevent one of the other serious outcomes listed in the definition

ABSTRACT

Background: Currently stroke represents a serious problem in public health as one of the leading causes of mortality and morbidity worldwide [1-3] with developing countries accounting for 85% of global deaths from stroke [1, 3-5]. In Uganda, stroke seems to be increasing and the case fatality is unacceptably high [6]

Good perceptions and knowledge of stroke; a population's knowledge of the stroke risk factor profiles; and good quality of acute stroke care have been shown to be effective strategies in improving stroke prevention and outcomes [7-9]. In Uganda, perceptions, knowledge of stroke and risk factors associated with stroke in defined urban and rural populations is currently not known. Stroke units are also non-existent in the country and there is no documented proof of practice using set protocols for acute stroke management.

Objectives: As a feasibility study of creating a stroke unit in a low resource setting as well as obtain information to develop targeted interventions in populations at risk for stroke, **Aim I**: **Objective 1** will determine the prevalence of known stroke risk factors and associated socio-behavioural characteristics among urban and rural populations in Wakiso district; **Objective 2** will assess the perceptions, knowledge of stroke and associated factors among urban and rural populations in Wakiso district; **Aim II** will determine the 30 day stroke outcomes and the effect of implementing the "Stroke care bundle" on the 30 day outcomes among adult stroke patients presenting to Mulago national referral hospital

Methods: Aim I is a subset of the MEPI-CVD survey of 5000 participants in urban and rural Wakiso district.

In Aim I Objective 1, a cross sectional study design will be used to determine the prevalence of stroke risk factors and associated socio-behavioural characteristics among adults living in urban Nansana town council and rural Busukuma Sub County in Wakiso district in Uganda. Following household enumeration and mapping, multistage sampling technique will be used to select a sample of participants aged 18 years and above. Data will be collected using a pretested modified version of the manuals on WHO STEP wise approach to stroke and risk factor surveillance (STEPS Stroke; http://www.who.int/chp/steps/Manual.pdf).The data collected from the population survey will be compiled and analysed according to the WHO manual guidelines. Logistic regression analysis will be used to identify the socio-behavioural characteristics associated with the three most prevalent known risk factors for stroke. Only socio-behavioural factors that are significantly associated with known stroke risk factors at bi-variable analysis ($P<0.05$) will be included in the adjusted model.

Objective 2 will assess perceptions and knowledge of stroke among participants 18 years and above living in urban Nansana town council and rural Busukuma sub County, Wakiso district in Uganda. Systematic sampling will be used to select participants from objective who will meet the inclusion criteria. Data will be collected using a pretested modified version of instruments used in previous studies on stroke knowledge and perceptions [10-12]**.** Data collected from the final survey instrument will be analysed using both descriptive and inferential statistics and the level of statistical significance will be set at $p<0.05$.

In Aim II, a non-randomised controlled trial (quasi-experimental study) design will be used. Patients presenting to Mulago hospital with stroke during the study period and meet the inclusion criteria will be enrolled consecutively into the pre-intervention group of the study until a sample size of 127 is attained. This is estimated to last six months immediately after which consecutive enrolment into the intervention group will begin until a sample size of 127 is attained. This is also estimated to last about 6 months. During the pre-intervention stage, participants' clinical, laboratory and imaging presentations will be assessed using standard procedures; validated tools used elsewhere; and certified laboratories. Each participant will be followed up for a period of 30 days to determine outcomes (mortality, disability and dependence) using validated tools used elsewhere. The intervention stage of the study will involve implementation of the "stroke care bundle" to the study participants. All the other information we seek to obtain during the pre-intervention stage of the study, will also be obtained. The effect of implementing the stroke care bundle on 30 day outcomes will also be determined. Data entry, descriptive and comparative statistical analysis will be performed. The level of statistical significance will be set at $p<0.05$.

CHAPTER ONE

1.0 INTRODUCTION

Generally a population's good perceptions and knowledge of stroke especially in regard to recognition of stroke warning signs, stroke risk factor profiles as well as good quality of acute stroke care have been touted as effective strategies in improving stroke prevention and outcomes [7, 9, 10, 13].

They enable communities to adopt healthy lifestyle changes which reduces the risk factors for stroke, improves adherence to medications to control medical risk factors as well as reduces the delays in presenting to hospital in case of a stroke event [7]. Good quality of acute stroke care enables early implementation of management strategies to reverse abnormal physiological findings and prevent complications resulting from stroke such as high temperatures, hyperglycemia, hypertension, infarct extension or re-bleed, aspiration, pneumonia all of which are associated with high mortality from stroke.

Although over the past four decades the above strategies to improve stroke prevention and outcomes were more prevalent in the developed countries, especially among Caucasians, the interest in adopting similar strategies in developing countries has recently increased [7, 9, 14]. Since 2000, there have been multiple reforms taking shape in developing countries, resulting from the awareness of the importance of tackling non-communicable diseases (hypertension, diabetes, stroke, heart disease), whose burden is fast increasing [15-17]. It is projected that these diseases will likely exceed communicable diseases by 2030 as the leading causes of death if nothing is done [16, 18, 19]. The increasing burden of stroke is because of the demographic transitions that have resulted in increase in the population of the elderly as well as rapid western cultural adaptation including sedentary lifestyle, deleterious health behaviour like consumption of high fat/cholesterol diet, alcohol and smoking. These have accelerated key vascular risk factors for cardiovascular disease and stroke such as hypertension, diabetes, hypercholesterolemia, sedentary lifestyle, obesity, heavy alcohol ingestion and smoking. Adoption of the strategies to prevent stroke and reduce mortality from stroke by developing countries is because currently stroke represents a serious problem in public health as one of the leading causes of mortality and morbidity worldwide [1, 3, 15, 17, 19-22] with developing countries accounting for 85% of global deaths from stroke [1, 3-5].

In Uganda, the burden of stroke seems to be increasing and mortality from stroke is high. Isolated studies both hospital and community based have shown that known stroke risk factors are highly prevalent [23-33]. There is however no data on local beliefs/understanding and knowledge surrounding stroke in these communities. Despite existence of stroke units for four decades, there is currently none in the country and neither is there a documented set protocol for the acute management of stroke patients at Mulago, Uganda's national referral hospital. It is in this context that we seek to conduct a feasibility study to subsequent creation of stroke unit care in a low resource setting by implementing a set protocol of stroke management ("Stroke care bundle") and evaluating its effect on 30 day outcomes (mortality, disability and dependence) as well as obtain information on stroke perceptions and knowledge among urban and rural populations in order to contribute to the development of targeted interventions to prevent stroke in populations at risk.

CHAPTER TWO

2.0 BACKGROUND

2.1 Burden of stroke in the world

Stroke is the second leading cause of death and leading cause of adult disability worldwide with 400-800 strokes per 100,000 [20], 15 million new acute strokes every year [34], 28,500,000 disability adjusted life-years [19] and 28-30 day case fatality ranging from 17% to 35% [18]. The burden of stroke will likely worsen with stroke and heart disease related deaths projected to increase to five million in 2020, compared to three million in 1998. This will be a result of continuing health and demographic transition resulting in increase in vascular disease risk factors and population of the elderly. Developing countries account for 85% of the global deaths from stroke [1, 3-5]. The social and economic consequences of stroke are substantial. The cost of stroke for the year 2002 was estimated to be as high as $49.4 billion in the United States of America (USA), while costs after discharge were estimated to amount to 2.9 billion Euros in France [35].

2.2 Burden of stroke in Africa

A systematic review of the existing literature to examine the burden and profile of stroke in the WHO African region reported an annual incidence rate of stroke of up to 316 per 100,000, a prevalence rate of 315 per 100,000 and a three year fatality of up to 84% in Africa [35]. Disabling stroke prevalence may be at least as high as in high-income areas [18].In 2002, model-based estimated age-adjusted stroke mortality rates ranged between 168 and 179 per 100,000 population for countries in the African region [35]. Case–fatality data available from three hospital based urban stroke registers in South Africa (two South African and one from Zimbabwe) found 30 day case fatality ranging between 33 and 35% [15]. Given the economic burden of stroke in the developed countries [35], a small fraction of such amounts can cause enormous economic damage to low income countries especially in SSA, given the younger age at which stroke occurs [36]. A study done in Togo estimated direct cost per person of 936 Euros (3,276,000 Uganda shillings) in only 17 days, about 170 times more than the average annual health spending of a Togolese [35].

2.3 Burden of stroke in Uganda

The actual burden of stroke in Uganda is not known. According to WHO estimates for heart disease and stroke 2002, stroke was responsible for 11 per 1000 population (25,004,000)

disability adjusted life years and mortality of 11,043 [37]. Stroke is one of the common neurological diseases among patients admitted to the neurology ward at Mulago, Uganda’s national referral hospital accounting for 21% of all neurological admissions [38]. Unpublished research done at Mulago hospital, showed a 30 day case fatality of 43.8% among 133 patients admitted with stroke [6]. The economic burden caused by stroke has not been explored in Uganda but given the very high dependant population (53%) [39], high prevalence of HIV/AIDS, drug resistant TB and Malaria, the impact of stroke and other emerging non-communicable diseases on the resource limited economy is astronomical.

2.4 Causes of stroke and subtypes

Stroke or a cerebral vascular accident is the sudden death of brain cells due to inadequate blood flow and oxygen resulting from a blood clot occluding an artery in the brain or a blood vessel rupturing [40]. When either of these things happens, brain cells begin to die and brain damage occurs. About two million brain cells die every minute during stroke with loss of abilities controlled by that area of the brain which include speech, movement and memory. A high number of dead brain cells are associated with increased risk of permanent brain damage, disability or death [40]. Stroke is divided into two broad categories that define its pathophysiology:

2.4.1 Ischemic stroke

Ischemic stroke is caused by blockage of arteries by blood clots or by the gradual build-up of plaque and other fatty deposits [40-42]. These may originate from the affected vessel or may embolise from other intracranial and extracranial vessels or from the aortic arch [40-42]. Majority of emboli originate from the heart as a result of valvular heart disease, arrhythmias, ischemic heart disease, bacterial and non-bacterial endocarditis and cardiomyopathies among others [40-42].

Regarding the prevalence of ischemic stroke, it accounts for 85-90% of all strokes in the developed world with average age between 70-80 years [1]. In Africa on the other hand, several studies have shown prevalence of ischemic stroke as low as 10-40% [43, 44]. In Uganda, an in-hospital study at Mulago hospital found 77.6% prevalence of ischemic stroke, mean age 62.2$\pm$ 16.5 years [23]. A large multicentre case control study that involved 3000 stroke patients from 22 countries including low and middle income, Uganda inclusive, showed 78% prevalence of ischemic stroke [45].

2.4.2 Hemorrhagic stroke

Hypertension is the most important risk factor for vessel rupture in the brain leading to haemorrhagic stroke [41, 45, 46]. Other causes of rupture of vessel walls include cerebral aneurysms, arteriovenous malformations use of anticoagulants and vasculitis [40, 41, 46, 47].

Regarding the prevalence of haemorrhagic stroke, it accounts for 10-15% of all strokes in the developed world and is responsible for more than 30% of all stroke deaths [1, 40, 41, 46]. In Africa on the other hand, several studies have shown prevalence as high as 20-60%. In Pretoria South Africa, 32.8% of 116 stroke patients had haemorrhagic stroke on brain CT scan [15]. Nakibuuka J et al 2012 found 22.4% prevalence of haemorrhagic stroke among 85 adult stroke patients at Mulago hospital in Uganda [23].

The effects of a stroke depend on where the stroke occurs in the brain and how much the brain is damaged, but the clinical symptoms of stroke do not accurately predict its underlying cause or causes. Classic stroke symptoms include the acute onset of unilateral paralysis, loss of vision, speech impairment, memory loss, impaired reasoning ability, coma, or death [48].

2.5 Causes of mortality from stroke

Death from stroke is as a result of co-morbidities and/ or complications. Complications of stroke may arise at different time periods. The beginning of stroke symptoms and the first month following the stroke onset is the most critical period for survival with the highest number of fatalities in the first week [47, 49]. Complications of stroke include hyperglycemia, hypoglycemia, hypertension, hypotension, fever, infarct extension or re-bleeding, cerebral oedema, herniation, coning, aspiration, aspiration pneumonia, urinary tract infection, cardiac dysrhythmia, deep venous thrombosis and pulmonary embolism among others [48]. During the first week from stroke onset, death is usually due to transtentorial herniation and haemorrhage, with death due to haemorrhage happening within the first three days and death due to cerebral infarction usually occurring between the third to sixth day.

One week after onset of stroke, death is usually due to complications resulting from relative immobility such as pneumonia, sepsis and pulmonary embolism [49]. Different studies have found varied factors associated with stroke mortality in their setting. For example, the most common predictors of death from stroke for those aged more than 65 years of age reported by Mackay [48] included previous stroke, atrial fibrillation and hypertension. Nigeria [50]

reported a 12.6% 30 day case fatality of all strokes. Among patients with hemorrhagic stroke: fixed dilated pupil(s), a Glasgow coma score of less than 10 on admission, swallowing difficulties at admission, fever, lung infection, and no aspirin treatment were independent risk factors for a lethal outcome. Yikona J et al also observed that stroke severity, neurological deterioration during hospitalisation, non-use of antithrombolytics during hospital admission and lack of assessment by a stroke team were the most consistent predictors of case fatality at seven days, 30 days and one year after stroke [50]. In Pretoria, South Africa, case fatality at 30 days was much higher, 22% for ischemic stroke, 58% for cerebral hemorrhagic stroke [51] and hypertension was significantly associated with stroke. At Mulago hospital in Uganda, 30 day case fatality of 43.8% was reported among 133 patients (mean age 65.8$\pm$ 15.8 years) with, fever > 37.5^0 (OR 2.81 (95%CI; 1.2-6.6) and impaired level of consciousness with a GCS <9 (OR0.13 95%CI; 0.005-0.35) significantly associated with increased mortality [6]**.**

2.5.1 Complications of stroke

Hyperglycaemia/hypoglycaemia

Hyperglycemia at the time of acute stroke is associated with poorer clinical outcomes, infarct progression and increased mortality especially in the first month of stroke, with non-diabetic patients more affected compared to diabetics [52-54]. It is also associated with reduced functional recovery. Plasma glucose levels above eight mmol/L after acute stroke predicts a poor prognosis after correcting for age, stroke severity and stroke subtype and should be treated actively [52]. The American Heart and Stroke Associations (AHA/ASA) [54], The National Stroke foundation of Australia (NSF 2010) [55], The National Institute for Clinical Excellence [56] recommend cautious treatment of patients with glucose concentrations greater than 8-11mmol/L with subcutaneous insulin. Hypoglycemia on the other hand may cause focal neurological deficits that can be reversed by treatment [53, 54, 57].

Fever

High body temperature within 24hrs from stroke onset is associated with poor outcome and large cerebral infarcts [58]. High temperature seems to be a major determinant even for long-term mortality after stroke. Hyperthermia acts through several mechanisms to worsen brain ischemia including: enhanced release of neurotransmitters, exaggerated oxygen radical production, more extensive blood brain barrier break down, increased numbers of potentially damaging ischemic depolarisations in the focal ischemic penumbra [59, 60]. Most common

causes include chest and urinary tract infections [61]. Regular paracetamol and/or physical cooling measures are reportedly adequate [62].

Blood pressure

Both hyper and hypotension in the first 24 hours after stroke are associated with poor outcome [55, 56, 63, 64], and poor short and long term prognosis [54]. Hypertension may indicate oedema, hemorrhage, and increase in the risk of primary ICH or hypertensive encephalopathy. Many hypertensive patients also have pre-existing hypertension that may or may not have been treated prior to the stroke [56]. According to AHA/ASA [54], for every ten mmHg increase above 180 mmHg, the risk of neurological deterioration increases by 40% and the risk of poor outcome increases by 23%. A study in AHA/ASA, found that an elevated baseline mean arterial BP was not independently associated with poor outcomes, but elevations in mean BP over the first days after stroke were. They also found that in most patients, the BP spontaneously decreases over 4-10 days from stroke onset. BP changes may occur as a result of disturbed cardiovascular autonomic regulation, with changes in absolute BP levels and BP variability both possible.

A Cochrane review (65 Randomized Clinical Trials) concluded that insufficient data exists to evaluate BP lowering post-stroke. Recommended based practice is based on clinical experience and expert opinion with the AHA guidelines recommending starting or increasing anti-hypertensives in ischemic stroke if systolic blood pressure >220 mmHg or diastolic blood pressure >120mmHg, unless end-organ damage is due to high BP. Outside of organ dysfunction, the BP should be cautiously lowered by not more than 10-20%, 15-25% in 24 hours (AHA>ASA). In acute ICH on the other hand, antihypertensive drugs including intravenously administered ones, can be used to maintain SBP<180mmHg (MAP 130mmHg). In the absence of intracranial pressure, neurosurgeons recommend 160/90mmHg (MAP 100mm Hg) [65]

Dysphagia

Dysphagia occurs in 27-55% of patients with new onset stroke [53]. Only about 50% of those affected recover normal swallowing by 6 months [53] It is associated with increased risk of complications such as aspiration, aspiration pneumonia, dehydration and malnutrition [66, 67], hence early screening to prevent these complications [66]. The National Stroke

Foundation [55] recommends that all patients should be screened for swallowing deficits before being given food, drink or oral medications. Screening should be undertaken by personnel specifically trained in swallow screening and a failed bedside screen should be followed by a complete assessment by a speech pathologist prior to any oral ingestion [53].

2.6 Acute stroke management

There have been major advances in the treatment of acute stroke in recent years [68] Fundamental to these advances have been; firstly the use of thrombolysis in ischemic stroke; secondly protocol driven multi-disciplinary care in stroke units to improve survival, independence and quality of life [14, 69-71]; and thirdly development of national guidelines to assist in protocol development and standardisation of care [14, 68].

When suspected of having a stroke or TIA, a rapid stroke screen is done to facilitate early referral to a stroke unit where available and also in eligible patients, being thrombolysed where possible [14].

A stroke unit is an area within a hospital where stroke patients are managed by a coordinated multidisciplinary team specialising in stroke management [14]. There is overwhelming evidence (31 RCT) that stroke unit care significantly reduces death & disability after stroke compared with conventional care in general wards for all people with stroke OR 0.82, 95% CI 0.73-0.92 [14]. Stroke units in low resource settings have also been associated with positive patient outcomes [72]. There are no stroke units in Uganda and a hospital based epidemiological study on stroke reported that patients suspected to have stroke presented on average two days post ictal [23]. It is recommended that in absence of stroke units, hospitals should adhere as closely as possible to the criteria of stroke unit care by use of the acute stroke care pathway [14, 72]. This pathway involves rapid assessment using validated tools, imaging using CT scan/MRI to confirm the diagnosis, routine investigations and added investigations for selected patients, thrombolysis, neuro and surgical interventions where possible, physiologic monitoring and management (GCS, vital signs), secondary prevention by addressing lifestyles modification, intervention to promote adherence to medications (antihypertensives, antiplatelet, anticoagulation, cholesterol lowering drugs, diabetes management) and lastly rehabilitation.

Unlike the acute stroke care pathway protocol that contains a lot of elements and therefore difficult to implement, a “stroke care bundle” contains a subset of evidence based care interventions that when combined define best care and significantly improve patient

outcomes. A Stroke care bundle also encourages clinicians to examine the way they deliver interventions through timely measurement of compliance and provides a method to improve the efficiency and effectiveness of care by standardising clinical care [73]. Testing use of a care bundle is therefore more feasible in our setting.

The validated Australian government "Emergency department stroke and TIA care bundle" [73] contains six key elements that are essential to evidence-based stroke care in the first 72 hours of patient admission (Appendix 1).

1. A rapid initial stroke screen using the ROSIER screening tool [74]
2. $ABCD^2$ assessment when TIA is suspected
3. Urgent CT or MRI
4. Nil by mouth until bedside swallow screen (within 24 hours) for stroke
5. Aspirin as soon as possible (within 48 hours of symptom onset)

   Physiologic monitoring and management (Neurostatus, blood glucose, temperature, blood pressure, hydration status)

2.7 Risk factors of stroke

2.7.1 Traditional risk factors associated with stroke

Stroke can occur in anyone regardless of race, gender or age however the chances of having a stroke increase if an individual has certain risk factors that can cause a stroke. The best way to protect oneself and others is to understand personal risk and how to manage it. Studies have shown that 80% of strokes can be prevented in this way [45, 75].

Stroke risk factors are divided into modifiable and non-modifiable. The modifiable risk factors are further subdivided into lifestyle risk factors or medical risk factors. Lifestyle risk factors which include smoking, alcohol use, physical inactivity and obesity can often be changed while medical risk factors such as high blood pressure, atrial fibrillation, diabetes mellitus and high cholesterol can usually be treated. A large multicentre (INTERSTROKE) case control study showed that there are ten factors that are associated with 90% of stroke risk and half of these are modifiable [45].

Non-modifiable risk factors on the other hand though they cannot be controlled, they help to identify individuals at risk for stroke.

2.7.1.1 Modifiable/ controllable risk factors

<u>Hypertension</u>

Hypertension is the force by which blood pushes against the arteries. If left untreated it can

weaken blood vessels and damage major organs such as the brain and lead to a stroke by accelerating atheroma and thrombus formation resulting into infarcts of large vessels, hyalinosis and fibrin deposition with resultant infarction of small vessel lacunes [76]. Hyalinisation within the small cerebral vessels results in the formation of charcot bouchard micro aneurysms that result into intraparenchymal haemorrhage from perforating vessels. It also leads to rupture of diseased vessels and arterial venous malformations. In autopsy series, hypertension accounts for 40-50% of patients dying of non- traumatic haematomas [42, 43].

Large prospective cohort studies and subsequent systematic reviews have shown hypertension to be the most powerful modifiable predictor of all strokes with estimated PAR ranging from 25-50% [45, 77-79]. There is also evidence to suggest that the PAR for hypertension may be influenced by ethnicity, stroke subtype, geographic location [45, 80].

Furthermore, a meta-analysis of 61 studies involving one million subjects (mostly Caucasian), reported a log-linear relationship between blood pressure and all stroke [77]. As many as 73 million Americans have high blood pressure (one in every four adults) and 31.6% are not aware they have it [81]. High blood pressure is still largely ignored as a public health problem in most developing countries despite a sharp rise in morbidity and mortality from diseases related to hypertension such as stroke [82].Also hypertensive patients might not bother to visit health facilities to have their blood pressure taken as it is largely asymptomatic [83]. Prevalence of hypertension in Uganda is not known but isolated community based studies report prevalence of 37.3-44% in rural and urban populations respectively [24, 30, 33]. A hospital based study in Kampala, Uganda reported 60% prevalence of hypertension among 85 adult stroke patients [23]. Research has shown that less than 20% of hypertension occurs in isolation. Metabolically linked risk factors such as diabetes, obesity, dyslipidemias, all of which lead to amplification of stroke risk, commonly co-exist with hypertension [84]. Excessive alcohol consumption, physical inactivity and a high salt diet can also lead to high blood pressure [84].

Alcohol

Long-term heavy ingestion of alcohol (more than two drinks daily) is a recognised risk factor for all stroke (RR 1.6; 1.4-2.0), particularly for hemorrhagic stroke (RR 2.2; 1.5-3.2) [85].This may be related to a decrease in HDL levels, reduced fibrinolysis, and increased platelet aggregation. Recent ingestion of large amounts of alcohol within 24 hours preceding stroke onset, which also includes non-habitual drinkers, has also been found to be a risk

factor in some studies [45].Reports from the Serenity centre for alcoholics' rehabilitation, 2010, shows that three million Ugandans consume alcohol excessively [86].

Obesity

Obesity and excessive weight put a strain on the entire circulatory system. It is strongly linked to cardiovascular diseases and type II diabetes mellitus (DM) through the promotion of insulin resistance and other associated physiological derangements, including dyslipidemia, elevated blood pressure and increased left ventricular mass. These lead to degenerative changes of vessel walls secondary to the process of atherosclerosis, fibrinoid necrosis of small arteries and arterioles with resultant cerebral infarction [87]. In SSA, the past two decades have seen a dramatic increase in obesity as the region experiences what is called a 'double burden' of disease with malnutrition on the one hand and a growing prevalence of obesity on the other.

Malnourishment in the form of both starvation and overconsumption of cheap and fried foods is increasingly pandemic in Africa largely due to increasing urbanization as jobs have moved out of rural areas and into cities. People from rural communities previously used to large amounts of physical labour and walking as well as an abundance of fruits and healthy grains to eat, have encountered in the cities a new world of less energy-demanding jobs and a plentiful supply of fried, cheap meats laden with trans fats [88]. Demographic and health surveys from seven countries in Africa (Kenya, Tanzania, Senegal, Ghana, Malawi, Niger and Burkinafaso) indicate that the prevalence of overweight/obesity increases by about 5% per year on average in these countries [89]. While the rate of change in urban overweight/obesity did not significantly differ between the poor and the rich, it was substantially higher among the non-educated women than among their educated counterparts.

In South Africa alone, 75% of the black population between the ages of 18 and 65 years and 50% of the white population are either overweight or obese (International Association for the Study of Obesity, IASO). Among certain tribes in Nigeria, women are traditionally fattened up before marriage to make them seem more attractive and healthy to their future husbands.

The prevalence of obesity in Uganda is currently not known but The Uganda Heart Institute predicts that obesity-related heart disease will be the leading cause of death in sub-Saharan Africa by 2020 [88]. Despite the growing problem, many sub-Saharan Africans do not find the region's increasing waistline to be of concern because of social attitudes that make being overweight seem harmless, if not explicitly attractive, "index of affluence and power is linked

to one's size." HIV/AIDS is another unlikely factor in the acceptance of obesity. The virus, known as "slim disease" throughout Africa, is strongly associated with weight loss. So being fat is viewed as a "great thing because it means you don't have HIV". Some Africans, purposely gain large amounts of weight to prove that they don't have the disease [88].

Physical inactivity

Studies have shown a consistent association between physical activity and risk of ischemic and hemorrhagic stroke. The INTERSTROKE case control study (3000 stroke patients from 22 countries) showed regular physical activity (OR 0.69, 0.53-0.90) and a PAR of about 29% which was larger than was reported in INTERHEART for acute myocardial infarction (MI), (12%) [45, 90]. People who exercise five or more times per week, have a reduced stroke risk. A meta-analysis of 31 observational studies confined to high income countries, reported that moderately intense physical activity reduced the risk of all strokes (RR 0.6; 0.5-0.9) with a comparable risk reduction for both ischemic and hemorrhagic stroke [91].

In Uganda, the prevalence of physical inactivity and sedentary behaviour among adults is not known however a national survey carried out in 2003 as part of the WHO 24 countries Global School based Student Health Survey on physical inactivity and sedentary behaviour among 4,218 school children (2,712 analysed, mean age 14.9 years) showed more than 80% physical inactivity among both boys and girls, with 26.2% (95% CI 22.8-29.5) sedentary boys and 25.8% (95% CI 22.2-29.3) sedentary girls [92].A hospital based study on the epidemiology of stroke among 85 stroke patients found the prevalence of physical inactivity of 40% [23].

Diabetes mellitus

Diabetes accelerates atherosclerotic vessel disease. The resultant thrombosis, embolism of large arteries and lacunar disease, especially in the elderly is commonly associated with other risk factors [93]. The incidence of type II DM which is known to increase the risk of developing heart disease and stroke has increased among black Africans in recent years. According to the International Association for the Study of Obesity (IASO), 8 to 12% of the black South African population has type II DM, compared with just 4% of the country's white population [88].In 1972, only 254 people in Uganda had been given a diagnosis of diabetes. Currently Uganda has 560,000 registered people with diabetes but it is thought that an additional 560,000 patients may have the disease but not be aware of it [94]. Prospective cohort studies have determined DM to be a strong risk factor for ischemic stroke [95, 96]. It

confers a fourfold increased risk of stroke compared to those that do not have the disease. There is also convincing evidence of a graded association with increases in HBA1c [97]. Diabetes is commonly associated with other risk factors. The prevalence of hypertension in diabetics has been reported between 22 to 54% [98]. Bateganya M et al, 2002 reported a 34% prevalence of hypertension among 100 DM patients at Mulago hospital in Uganda [99].

Cigarette smoking

Nicotine contained in cigarettes raises BP which causes rupture of aneurysms and arterial venous malformations. Carbon monoxide produced during smoking reduces the amount of oxygen to the brain and cigarette smoke increases the amount of fibrinogen which increases the chance of clotting. In addition, increased serum proteolytic activity of smokers degrades the collagen of the plaque's fibrous cap, which makes it susceptible to rupture [100]. In a number of studies, smoking has been shown to be strongly associated with an increased risk for all strokes and subclinical carotid disease, with a graded linear association for the number of cigarettes smoked [45]. In Uganda, a study of high school students in Kampala (n= 2,789) and the rural tobacco growing town of Arua (n= 1528) found a current smoking prevalence of 5.3% in Kampala compared to 21.9% in Arua [31]. The Global youth tobacco survey carried out in Uganda in 2007 reported lifetime smoking prevalence rate of 16.6% among 13-15year old high school students [92]. Case control studies have also found an association between environmental tobacco smoke and increased risk of stroke [101].

Cardiac causes of stroke

Cardio-embolism is an important cause of ischemic stroke worldwide. In high income countries it accounts for 15 to 25% of all ischemic strokes. Limited data from low-income countries supports a similar frequency of cardio-embolism [23, 45]. Important causes of cardioembolism in high income countries include atrial fibrillation (AF), aortic arch disease and myocardial infarction [102]. On the other hand, in low income countries, rheumatic heart disease in addition to factors mentioned above, remains prevalent and is a frequent cause of premature stroke in young patients in SSA [103, 104]. AF is the most important factor in the occurrence of cardio embolism with a four to seven fold risk of systemic embolisation when compared to comparable groups of patients without AF [105]. The risk becomes greater with older patients. The incidence of emboli varies from 9 to 27% in clinical reports and increases to 41% in necropsy studies [106]. Fleming et al noted a 25% incidence of emboli among 500

patients with AF [107]. O'Donell et al 2010 reported that AF was the most common cardiac source of thromboembolism in cases with ischemic stroke 203 (9%), with regional variation in prevalence: 86 (23%) in high-income countries, 14 (13%) in South America, 16 (7%) in Africa, 41 (6%) in India, and 46 (5%) in Southeast Asia[45]. Cardiac aetiology was associated with an increased risk of ischemic stroke, but not intracerebral hemorrhagic stroke (OR 0·90, 0·52-1·56). Studies have identified three clinical predictors of subsequent thromboembolism: hypertension, recent onset of congestive heart failure (CHF) and previous history of thromboembolism [108].

Dyslipidemias

Hyperlipidemia causes stroke secondary to thrombosis and embolism resulting from degenerative vessel changes due to the process of atherosclerosis [109]. It is arguably the strongest risk factor for coronary heart disease (CHD) with an estimated PAR of over 50% [110]. The relationship between cholesterol and stroke however is much less certain. For ischemic stroke, epidemiological evidence supporting an association has been inconsistent [111, 112]. A meta-analysis of 45 prospective cohort studies reported no significant association between total cholesterol and ischemic stroke (RR 0.8; 0.6-1.1) [77]. However, a large multicentre case control study (3000 stroke patients from 22 countries, Uganda inclusive), found that increased concentration of total cholesterol was not associated with risk of ischemic stroke, but was associated with reduced risk of intracerebral hemorrhagic stroke [45]. Additionally increased concentration of HDL cholesterol was associated with a reduced risk of ischemic stroke, and an increased risk of intracerebral hemorrhagic stroke. A hospital based study in Kampala, Uganda found 31% prevalence of high total cholesterol (>200mg/dl) among 65 patients with ischemic stroke [23].

2.7.1.2 Non-modifiable/uncontrollable stroke risk factors

Age

Advanced age is associated with degenerative changes of the vessel wall resulting from hypertension alone or in connection with atherosclerosis, combined with hemodynamic action and natural weak points in the cerebral vessel wall leading to potential sites for rupture, thrombus formation and thromboembolism [113]. Advanced age is a risk factor for both first and recurrent stroke, with doubling of stroke risk in each decade over age 55 years [114]. There is a seven fold greater risk of dying from stroke than the general population, in

more than 65 years age group [114]. Some risk factors tend to be more prevalent with advancing age such as hypertension and diabetes mellitus whereas others are acquired at a younger age like smoking and thus age can be considered a marker for the duration of exposure to a risk factor [114, 115].

Gender

Male mortality from stroke has been noted to exceed that of females in all age groups less than 70 years, with males having a 2.5 fold increased risk of stroke than females [114].Differences in frequency of key vascular risk factors have been implicated [116]. Gender differences in risk factor profiles may predict differences in outcomes or responses to therapies therefore prevention strategies and public health efforts need to reflect these differences [116].

Family history of stroke

This is an independent risk factor for ischemic stroke with onset before 70 years. The association is not only for large and small vessel disease but also for cryptogenic stroke [117]. In a study of a cohort of men, the relative risk of stroke with positive paternal history was 2.4 fold and relative risk of stroke with maternal history of stroke was 1.4 fold [114].

Previous history of stroke

Stroke recurrence after an initial stroke has varied widely from 3% to 22% at one year and 10% to 53% at five years in different studies [114, 118].

2.7.2 Emerging Risk Factors

HIV/AIDS

Clinical, radiological and post-mortem studies have found a strong association between HIV/AIDS, ischemic stroke and intracerebral haemorrhage [119, 120]. Vascular abnormalities, coagulation disorders and cardioembolism have been identified as the main causes of stroke among HIV\AIDS patients [119-121]. HIV-associated dilated cardiomyopathy as a cause of cerebral infarction resulting from cardioembolism is well described in HIV infection [121].

A prospective analysis of stroke in 35 black South African HIV patients (mean age 32.1 years) reported ischemic stroke with coagulopathy as the commonest cause (60.7%) and,

protein S deficiency accounted for the majority (64%) [122]. The study also reported stroke to be the first manifestation of HIV infection in 20 out of 35 patients. In Uganda, the current national HIV sero-prevalence stands at 7.3% [123] up from 6.4% in 2011 [123]. A descriptive epidemiological study among stroke patients at Mulago hospital, with mean age 62.2± 16.5 years, found only 5 out of 85 stroke patients to be HIV positive. All the HIV positive patients in this study presented with ischemic stroke [23].

Psychosocial stress

This appears to be an important risk factor for stroke but studies are limited and the constructs of psychosocial stress are often imprecise [124, 125]. Globally, the PAR of implicated factors such as depression, perceived stress, social isolation and lack of social support for acute myocardial infarction was about 30% [126]. A large multicentre (22 low and middle income countries, Uganda inclusive) case control study, reported a PAR of 4·6%, (2·1-9·6) for psychosocial stress and PAR 5·2%, (2·7-9·8) for depression with consistent estimates for ischemic and intracerebral hemorrhagic stroke. Depression was associated with an increased risk of all stroke and ischemic stroke, but not intracerebral haemorrhagic stroke (OR1·11, 0·82-1·52) [45].

Sickle cell disease

Sickle cell disease has recently been recognised as a problem of major public-health significance by the WHO with more than 70% of sufferers living in Africa [127]. In Uganda, 25,000 babies with SS disease are born each year [128]. Stroke in various forms is the most common neurological complication known to occur in these patients. Vascular occlusions by sickled cells give rise to the pathological changes underlying the common neurological complications in this disease. These changes tend to be multiple and repetitive [129, 130]. They give rise to very varied and recurrent clinical manifestations of this complication. Nantulya et al, 1989, found 24.4% neurological complications among 25 children with SCA in Aga Khan Hospital in Nairobi, Kenya [130].

Diseases of the vessel wall

Vasculitides such as polyarteritis nodosa, temporal arteritis; collagen vascular diseases like systemic lupus erythematosus, rheumatoid arthritis, syphilitic vasculitis, are known causes of stroke [131].There is activation of complement cascade by antigen-antibody complexes

lodging into gaps between endothelial vessels, with resultant production of lysozymes and destruction of vessel wall with haemorrhage and fibrinoid necrosis [131].

Hypercoagulable states

Disseminated intravascular coagulation, haemoglobinopathies, thrombocytopenia, antiphospholipid antibody syndrome, hyperhomocysteinemia, deficiency of antithrombin III, Protein S and C and hyperfibrinogenemia lead to disturbance of normal properties of blood increasing the risk of cerebral infarction by formation of clots and cerebral hemorrhage. Antiphospholipid antibody syndrome and hyperhomocysteinemia associated with arterial thrombosis are particularly known to significantly increase risk of infarction [93, 132]

Most epidemiological studies have reported a consistent and linear association between elevated levels of homocysteine and risk of ischemic stroke with meta-analyses reporting a 1.5 to 2 fold increase in risk [93, 132]

.

Table 1: Summary of prevalence of stroke associated risk factors in Ugandan studies

	**Author**	**Habitat<br>Age group**	**Association/Comments**	**Prevalence**
**1**	Mayega RW et al<br>PLoS one.<br>2012;7(10):e47632[24]	Rural<br>Age 35-60yrs	 Hypertension<br>Overweight<br>Obesity	N=1,656<br>20.5%<br>18%<br>5.3%
**2.**	Maher D et al<br>J Hypertens.<br>2011;29(6):1061-8[28]	Rural<br>(Masaka)<br>Age ≥13yrs	 Hypertension<br>Abdominal obesity in females<br>Current smoking<br>Males<br>Females	N=5372<br>22.0%<br><br>71.3%<br><br>13.7%<br>0.9%
**3.**	Wamala JF et al<br>Afr Health Sci.<br>2009;9(3):153-60[133]	Rural<br>(Rukungiri)<br>Age ≥20yrs	 Hypertension	N=842<br>30.5%
**4.**	Mondo Charles Kizza et al<br>Uganda Medical Journal.<br>2012;Vol 1:issue 001,10-16[30]	Rural<br>(Kasese)<br><br>Age ≥18yrs	 Hypertension<br>Males<br>Females<br>Overweight<br>Males<br>Females<br>Obesity<br>Males<br>Females	N=611<br><br>22.1%<br>20.5%<br><br>15.0%<br>16.8%<br><br>4.9%<br>9.0%

			Diabetes<br>Fasting blood sugar>6.1mmol/l<br>Males<br>Females<br><br>Physical inactivity<br>Daily smokers	7.2%<br><br><br>10.0%<br>31.0%<br><br>51.0%<br>9.6%
**5.**	Baalwa J et al<br>Afr Health Sci. 2010 Dec;10(4):367-73[25]	Urban (Kampala)<br>Rural (Kamuli)<br>Age 18-30yrs	Obesity<br>Urban<br>Rural<br>Overweight<br>Urban<br>Rural	N=683<br>4.4%<br>0%<br><br>10.2%<br>10.6%
**6.**	Bimenya GS et al<br>Afr Health Sci.2006 Sept;6(3):139-44[26]	Urban (Kampala executives)	Hyperlipidemia and other dyslipidemias	N=174<br>LDL 48%<br>TG 47%<br>TC 66%<br>LDL/HDL 12%<br>TC/HDL 53%<br>TG/HDL 68%
**7.**	Mpabulungi L et al<br>Croat med J.2004 Feb;45(1):80-3[31]	Urban (Kampala)<br>Age 13-15yrs	Current smokers	N=2789<br>5.3%
**8.**	Muula AS<br>Afr Health Sci 2007 Mar;7(1):45-9[32]	Urban (Kampala)<br>Age 13-17yrs	Tobacco smoking	N=<br>5.6%
**9.**	Kayima J et al<br>Cardiovascular [33]	Urban (Kampala)<br>Rural (Wakiso)<br>Age ≥18yrs	Hypertension<br>Urban<br>Rural	N=315<br>32.4% overall
**10.**	Lasky D et al<br>Nutrition. 2002 May;18(5):417-21[27]	Urban (Kampala)<br>Rural (Mukono)	Type 2 DM	N=440<br>8.1%

2.8 Prevention of stroke

More than 70% of strokes are first events [134], thus making primary stroke prevention a particularly important aspect. Interventions should be targeted at behaviour modification [3, 4, 134], which however requires information about the baseline perceptions, knowledge and prevalence of risk factors in defined populations.

2.8.1 Perceptions of stroke

<u>Beliefs regarding stroke</u>

Even though stroke affects more people each year than any other neurological illness worldwide, it is often misinterpreted or confused with other conditions [135]. The most common myths about stroke in the Western world indicate that it is a disease of the heart, strikes the elderly, cannot be treated and is unpreventable [7]. In SSA on the other hand, though local beliefs and understandings surrounding stroke are poorly understood, they differ from the common myths about stroke in the Western world [12]. Published studies on this subject in Africa, reported that stroke-like symptoms were considered both a physical and social condition resulting from natural or environmental causes and supernatural causes such as demons and witchcraft [12, 136, 137]. Therefore both traditional and Western medicines were used for treating stroke. Although Western health care treatment was used, it was regarded as culturally inappropriate[12].

In Tanzania, studies report that access to health care is strongly influenced by cultural knowledge and interpretation of disease symptoms [138, 139], structural and gender constraints [139, 140] and trust in providers [141]. For example, a study on perceptions of stroke among 80 participants in urban (Dar-es-Saalam) and rural (Hai) areas of Tanzania, found that stroke in Dar-es-Saalam was widely believed to emanate from supernatural causes (demons and witchcraft) that some respondents referred to as satan, devil or evil spirit. Such demons intentionally attack a person, in association with witchcraft, or strike accidentally. Witchcraft allegations involving stroke were mainly associated with conflict and jealousy within the community, conflict for land between neighbours or jealousy related to social advancement (e.g. taking children to school).

On the other hand, in Hai, explanations drew mostly on ‘natural’ causes (hypertension, fatty foods, stress). Stroke was widely understood to be caused by high BP, a view which arose largely through interactions with health practitioners (doctors, nurses and village health workers) who also told them that it should be controlled by medication and dietary salt reduction. In Dar-es-Salaam, by contrast, only a handful of respondents reported that high BP causes stroke, a consequence of reduced contact with health professionals or to dominance of other belief systems. Many more respondents mentioned stress as the cause of stroke in Hai, compared with Dar-es-Salaam. They said life had become difficult and people tended to think and worry a lot about many things such as how they would feed their families or pay their children’s school fees. In both sites, stroke was also said to be the expression of ‘God’s will

or resulted from angry ancestral spirits. These different beliefs and explanatory models fed into treatment-seeking behaviours[12].

Actions regarding stroke

The actions taken by people in case of a stroke largely depend on the population's characteristics and medical services. In developed countries with functional EMS, researches on action following a stroke are geared towards activation of this service or not [7, 8]. Activation of EMS is critical in timely arrival at a hospital with a stroke centre to enable patients benefit from evidence based advanced practices such as use of recombinant tissue Plasminogen activator (rtPA) in ischemic stroke, band ligation or coiling for ruptured aneurysms etc. Timely intervention has been proven to lead to better outcome. In a systematic review of 39 studies done in the developed world, between 27 and 100% of participants stated that they would call the EMS or visit a hospital emergency department in case they or a relative were experiencing a stroke [7, 142, 143]. However, when stroke patients were asked about what they had actually done at the onset of symptoms, only 18% had called the emergency services.

On the other hand, EMS is largely unknown in SSA as it is only accessible in very few communities in very few countries. Advanced practices of acute stroke management (use of rtPA in ischemic stroke, band ligation or coiling for ruptured aneurysms) are also almost non-existent [35]. Regarding actions after stroke therefore, medical pluralism based on multiple illness theories and religious ideologies is a common feature of treatment seeking (visiting doctors, self-treatment, healers, prophets and churches) in SSA [12, 141, 144, 145]. In the above study in rural Hai and urban Dar-es-salaam for example, the first treatment seeking option for the majority in Hai where the populations enjoyed frequent interactions with medical practitioners, was hospital, while in Dar-es-Salaam, with a dominance of cultural beliefs whose populations therefore widely believed that stroke emanated from supernatural causes such as demons and witchcraft, they sought the help of traditional healers. In both the urban and rural sites, multiple treatment options (serially or simultaneously) were the norm. Analysis of patient and carer narratives suggested that causation beliefs outweighed other factors, such as cost and distance, in shaping effective treatment.

In Uganda, local beliefs surrounding stroke and treatment seeking behaviours are currently not known.

2.8.2 Knowledge of stroke

Knowledge of stroke warning signs/symptoms and risk factors in the general population is associated with improved early presentation to hospital following occurrence of stroke for effective acute management, and enhancing success of primary prevention of stroke initiatives [7, 8, 10, 11, 13, 35, 146-148]. Stroke warning signs and symptoms which tend to occur suddenly include any of the following; painless weakness on one side of the body, numbness or dead feeling on one side of the body, painless loss of vision in one or both eyes, loss of one half of vision, loss of the ability to understand what people are saying and loss of the ability to express oneself verbally or in writing. Multiple studies including systematic reviews done in developed countries mainly among Caucasians, and few studies done in Africa to assess the knowledge of stroke among the general population, health workers, stroke patients and carers of stroke patients, generally reported poor levels of knowledge about recognizing and preventing stroke [7, 8, 10, 11, 146, 148].

Regarding the knowledge of warning signs and associated factors, an integrative review of 39 studies out of 169 on stroke knowledge and awareness done in North America, Europe, Asia, UK and Australia, with few studies done in North America including only African Americans that were originally English speaking showed that the ability to name one symptom of stroke varied significantly between studies from 25 to 100%. When asked open ended questions, the ability to name one symptom of stroke was 25 to 72% and it was 95 to 100% when asked closed ended questions. Older members of the population, ethnic minority groups (African Americans and Hispanics) and those with lower levels of education had consistently poor levels of stroke knowledge. For example, 47% of participants less than 65years knew a symptom/sign of stroke compared to only 28% of participants more than 65 years [7]. A systematic review from a gender perspective showed that better knowledge of stroke was observed among women compared to men in majority of the studies although there was a general lack of knowledge in both genders [8]. Why these particular groups have poorer levels of health knowledge is not fully understood [7].

Regarding the knowledge of risk factors for stroke and associated factors, Jones S et al in an integrative review of 39 studies on stroke knowledge and awareness showed that the ability to name one risk factor for stroke ranged between 18% to 94% when asked open ended questions and 42% to 97% when asked closed ended questions. Only 36% identified high BP as a main risk factor for stroke when asked open ended questions. Other risk factors

commonly identified without the prompt of a question included stress, diet, alcohol excess, inactivity, older age and smoking as causes of stroke. In contrast, when given options, more than 80% recognised high BP, previous stroke and a family history of stroke as risk factors. Consistently poor levels of knowledge of stroke risk factors in these studies were found among older members of the population. Approximately 50% of patients less than 65years were aware of their own personal risk factors for stroke, compared with 30% of those aged 65years or more. Inadequate knowledge of risk factors was also common among African Americans and people with low levels of education [7].

Literature search revealed only two studies done in Africa on knowledge of stroke warning signs and risk factors and both were done in Nigeria among health workers and the general population [10, 13]. Among 900 adults, mean age of 43.6+/-17.63 years in the general population in Osogbo, Nigeria, knowledge of stroke warning signs and risk factors was good with 80.1% of respondents aware of the risk factors and 76.9% of the warning signs. However, their baseline knowledge about stroke was poor. The most common risk factors identified by respondents were hypertension (78.2%), stress (59.9%) and old age (58.3%). Lack of exercise and obesity were less recognised by 18.9% and 11.9% respectively. Knowledge of stroke risk factors was found to be associated with age (p=0.001) and educational status (p=0.0018), while knowledge of established warning signs of stroke was associated with educational status [10]. In Uganda, the public's knowledge of stroke risk factors, warning signs and associated factors is not known.

Regarding other factors associated with stroke knowledge, among populations at high risk for stroke because of presence of known major risk factors and in whom stroke knowledge was hypothesised to be good, studies generally observed poor knowledge [149, 150]. A study done among 400 Omani patients with stroke risk factors, 98% had not been advised by their attending physician that their clinical conditions were risk factors for stroke [149].

Among stroke support group members (stroke patients, care givers), a cross-sectional questionnaire survey among 133 members of eleven German stroke support groups reported that overall, members were well informed about all aspects of modern stroke care. In this study 80.3% had good symptom knowledge, 64.7% had good risk factor knowledge, and 79.7% had good action knowledge. Stroke knowledge was excellent in 44.0% of subjects.

Age less than 70 years and not having had a stroke were significant predictors for excellent stroke knowledge [151].

Even though higher level of education and being a clinical worker correlated better with stroke among hospital workers in an African community, studies demonstrated gaps in their stroke knowledge [13].

2.8.3 Sources of information

Several studies on stroke knowledge in developing countries especially among Caucasians, have also studied the main ways in which participants gained information about stroke [7]. Sources varied according to population characteristics and for any given source there was often a big difference between studies in terms of the number of participants who had gained information from that source. People generally obtained information about stroke from family and friends and this was in agreement with majority of studies in the developed countries [7, 152]. In Osogbo, Nigeria however, the most common source of information was doctors and hospital personnel. Less than a tenth of the respondents listed family members as their source of information [10]. In Tanzania, a study of urban rural contrasts in explanatory models and treatment seeking behaviours observed that interactions with medical practitioners was the main source of stroke knowledge in rural Hai populations while in urban Dar-es Salaam, with a dominance of cultural beliefs and hence less interactions with medical practitioners, traditional healers were the main source of information [12].

Other sources of information from studies included, personal experience through knowing a stroke survivor or their family and a variety of media (television, radio) [7, 152]. Methods of mass media were the most commonly cited sources of stroke information in a study in Ohio in the USA with television identified as the most common source followed by newspaper and magazines. Even though doctors was the fourth most commonly cited source of information identified by respondents in this study, it was also the most commonly cited source of information among all individuals 75 years or older among blacks[153].

From multiple studies, literature as a source of information included books, magazines, pamphlets and newspapers. Stroke campaigns from schools, internet and public libraries were also cited [7, 152], though internet and libraries were the least accessed sources of information, cited in only three studies by 1 to 3% of participants.

In Uganda, the sources of stroke information among the general public is not known.

CHAPTER THREE

3.0 STATEMENT OF THE PROBLEM

Stroke is becoming a serious problem in public health in developing countries, Uganda inclusive [1, 3, 15, 17, 19, 20]. Firstly, western cultural adaptations over the past three decades have led to an increase in vascular risk factors such as HT, DM, cardiovascular diseases, obesity, smoking and heavy alcohol consumption with resultant increase in stroke [1]. Secondly, knowledge of stroke warning signs and risk factors as well as perceptions of stroke by the general population, although well described have been found to be generally poor among those with low levels of education and ethnic minorities in developed countries and in Africa. These factors govern control and prevention of risk factors of stroke as well as treatment seeking behaviours [7, 10, 12]. This has also resulted in increase in stroke and poor outcomes. Thirdly, the absence of stroke units in Uganda and lack of use of set protocols for stroke management has resulted in unacceptably high case fatalities from stroke in hospital studies [6].

According to WHO estimates for heart disease and stroke, in Uganda in 2002, stroke was responsible for 11 per 1000 population (25,004,000) DALYs and mortality of 11,043, which figures were projected to rise exponentially [37]. Unless this trend is dramatically reversed, an estimated 26,850 of our nation's bread winners will die a premature death per year while survivors suffer from life time disability with resultant increase in the already high dependant population; 53%, of Uganda's population [39]. When coupled with the already existing high prevalence of HIV/AIDS, drug resistant TB and Malaria, the impact of stroke on the resource limited economy is astronomical.

Strategies to improve stroke prevention and outcomes are still insubstantial and unknown in Uganda in the sense that there is absence of documented proof or effects of its practice. These strategies include improving the perceptions and knowledge of stroke in the general population, prevention and control of known risk factors for stroke and acute management of stroke patients in stroke units.

CHAPTER FOUR

4.0 RESEARCH OBJECTIVES

Aim I

1. To compare the prevalence of stroke risk factors and their associated socio-behavioural characteristics among urban and rural populations in Wakiso district.

2. To assess the perceptions and knowledge of stroke, and associated factors among urban and rural populations in Wakiso district.

Aim II

1. To determine the 30 day outcomes and associated factors among adult stroke patients presenting to Mulago national referral hospital.

2. To evaluate the effect of implementing the "Stroke care bundle" on the 30 day outcomes among adult stroke patients presenting to Mulago hospital.

4.1 RESEARCH HYPOTHESIS

Aim I

Risk factors for stroke; perception and knowledge of stroke is associated with one's social, economic and demographic characteristics (sex, education status, occupation)

Aim II

There is a reduction in 30 day mortality of 15% and significantly better functional state among patients presenting with stroke when "the stroke care bundle" is implemented.

4.2 Justification of the study

In Uganda, stroke seems to be increasing and mortality is unacceptably high. Stroke associated risk factors are also highly prevalent in the communities. "Risk factors of today are the diseases of tomorrow" [154].

Correct perceptions and adequate knowledge of stroke (warning signs, risk factors, risk factor profiles and associated socio-behavioural characteristics) as well as good quality of acute stroke care have been touted as effective strategies in improving stroke prevention and outcomes[7]. They govern and enhance the success of targeted interventions to control and prevent stroke and its risk factors especially among populations at risk, through adopting healthy lifestyles changes, improving treatment seeking behaviour and adherence to medications to control medical risk factors. They also reduce the delays in presenting to hospital in case of a stroke event. Set protocols for acute stroke management enable early implementation of treatment strategies to reverse abnormal physiological findings and prevent complications [9]. They also test the feasibility of creation of a stroke unit.

Currently knowledge regarding these effective strategies is lacking and given the economic constraints faced by our country, resources must be judiciously used to optimise care for those already affected and to formulate and implement effective strategies to prevent stroke.

A comprehensive study to contribute to this knowledge gap is therefore required.

4.3 Significance of the study

This project which seeks to improve stroke prevention and outcomes through research, advocacy and service training will contribute in 4 major ways

1. The study will contribute to the growing body on works on Non Communicable Diseases at the Ministry of Health and other stake holders by providing information to understand better the perceptions and knowledge of stroke, socio-behavioural characteristics associated with highly prevalent stroke risk factors, and effect of a validated set protocol of acute stroke care on outcomes post stroke.

2. The study will provide the initial infrastructure and opportunity of setting up a stroke unit at Mulago national referral hospital.

3. This study will provide preliminary data that will guide the development of targeted interventions to prevent stroke in populations at risk.

4. The research will be used as a stepping stone for further research on improving stroke prevention and outcomes in Uganda, results of which can be compared between and among populations and countries.

4.4 Figure 1: Conceptual Framework

Theoretical underpinnings of comprehensive stroke prevention and management

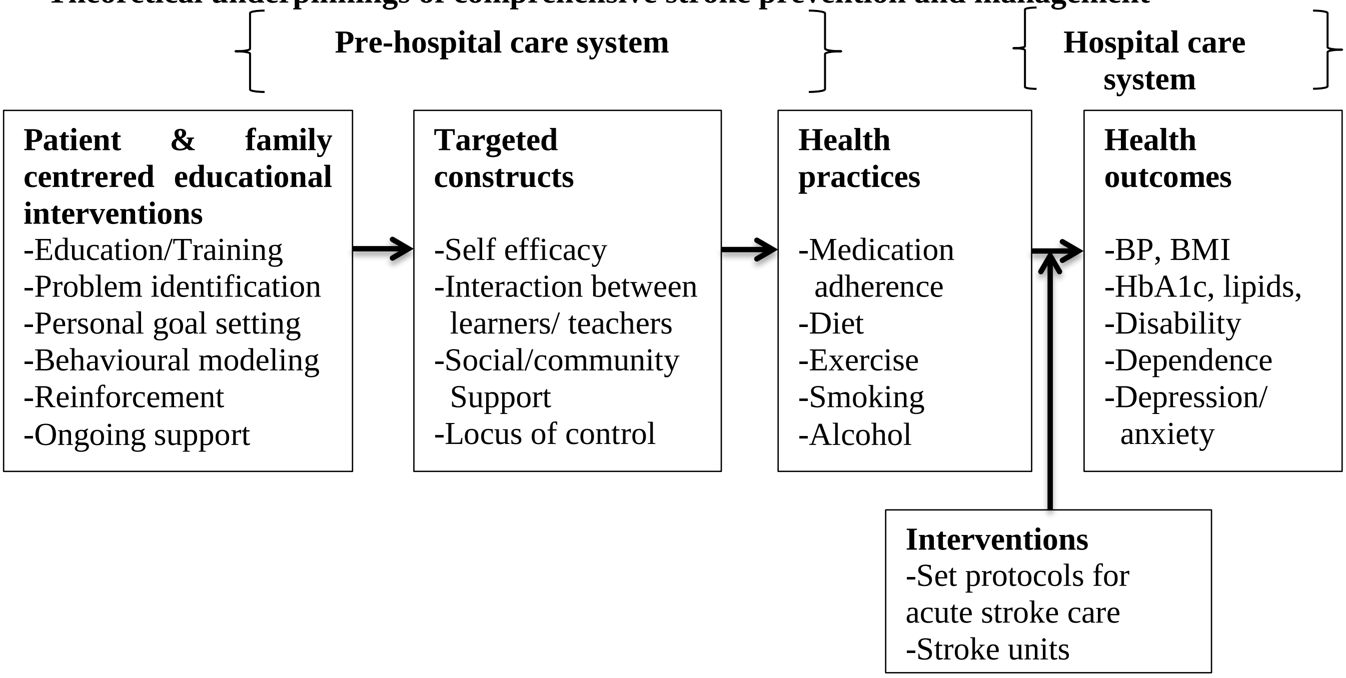

CHAPTER FIVE

5.0 METHODOLOGY

5A.0 METHODOLOGY FOR AIM I

5A.1 Study area and population

The study will be conducted in Wakiso district located in central Uganda. Wakiso covers an area of 2,807.75 square kilometres and is bordered by Kalangala Islands (in Lake Victoria) to the South, Mpigi and Mubende districts to the West, Luwero to the North, and Mukono district to the East. It is the second most populated district in the country with a current estimated population of 1,310,000 [39] and an annual growth rate of 4.1%. In 2002 census and projections to 2010, 53% of the population was <18 years of age [39].

Figure 2: Showing the location of Wakiso district on the map of Uganda

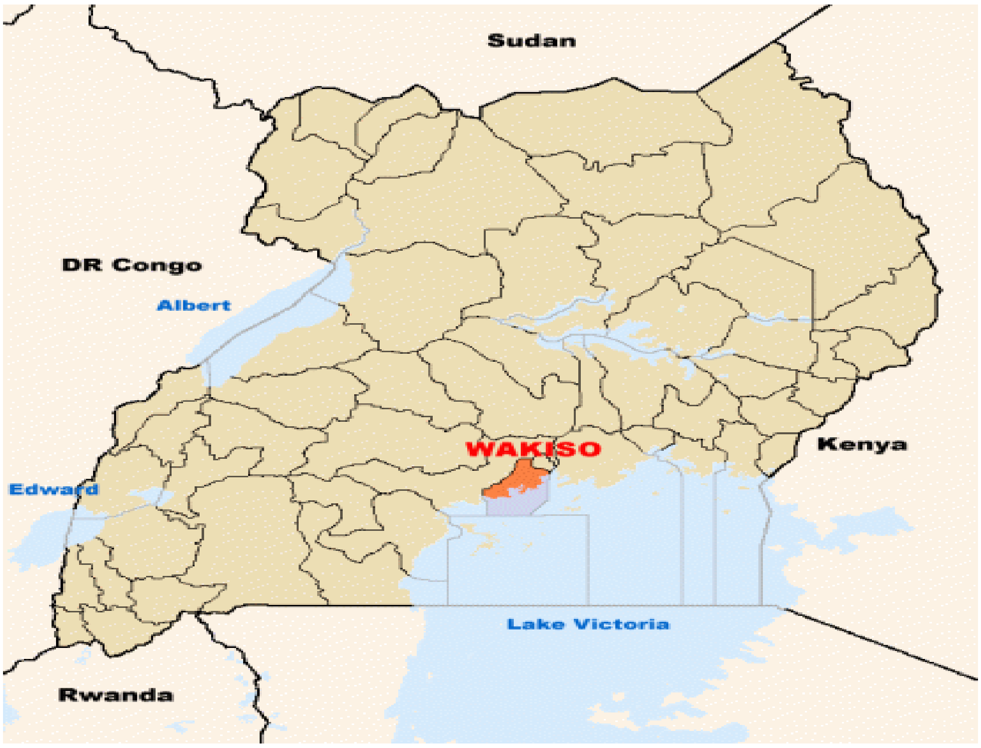

This district surrounds the capital city, Kampala, and because of this some of the sub parishes such as Kireka, Bweyogerere, Zana, Entebbe and parts of Kawempe, are “functionally” part and parcel of Kampala. The district is unique in that it has areas with markedly different levels of socio-economic development, ranging from peri-urban neighbourhoods (bordering the city) to typically rural areas. Most people (92%) live in rural areas. The district is heterogeneous, the population being made of people of varied ethnicity [39]. Most of the

people understand or speak Luganda, a local Bantu dialect of the Baganda, the indigenous tribe of the region whose main occupation is subsistence farming.

The district has a total of eighteen sub counties. The urban sub counties include Makindye, Wakiso town council, Kira town council, Entebbe municipality and Nansana town council while the rural sub counties include kakiri, Katabi, Masuulita, Namayumba, Nsangi, Ssisa, Busukuma, Gombe, Nabweru, Nangabo, Bussi, Mende and Kasanje. For this study Nansana Town Council and Busukuma Sub-Counties, were selected as the study sites. Nansana TC is an urban, highly populated sub-county with majority of its population running small businesses as their main source of income. Busukuma sub-county is a predominantly rural sparsely populated sub-county where majority of the population are subsistence farmers.
Figure 3 below shows the location of Nansana Town Council and Busukuma Sub-Counties

Figure 3: Map of Wakiso District showing the location of the selected study areas.

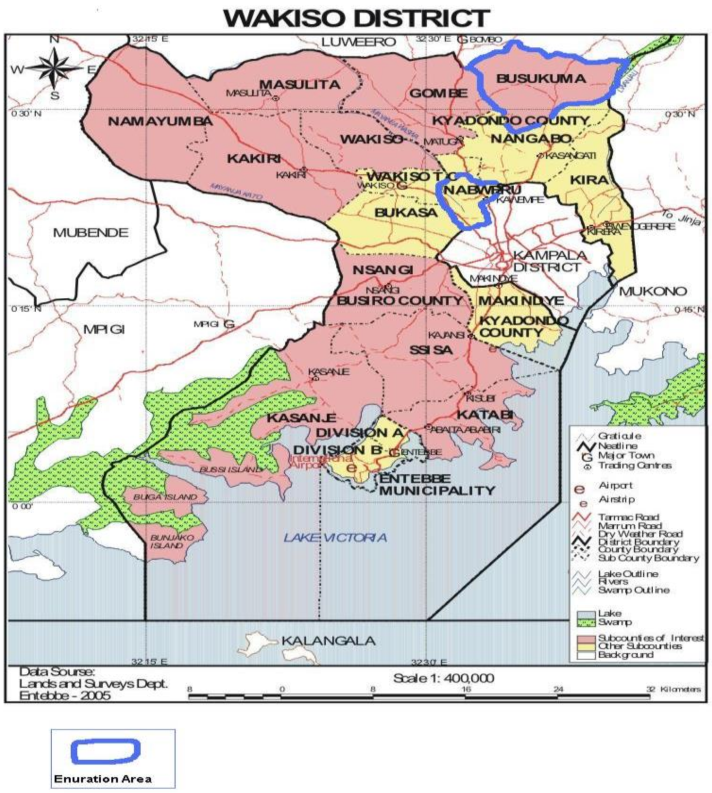

Wakiso district has one hospital; Entebbe hospital (grade A and B) and six (6) health centre IVs. In addition there are several public health centres III & II and a number of private health

facilities. Table 2 summarizes the demographic characteristics of the selected sub-counties, in comparison to the national figures.

Table 2: Characteristics of the selected sub-counties and Wakiso district

Characteristic	Nansana TC	Busukuma	Wakiso	National
Population	93,700	41,100	1,260,000	32,000,000
Number of households	26,575	13,091	317,000	N/A
Persons per household	3.5	3.1	4.1	4.7

5A.1.1 Study design

This will be a cross-sectional study to take place in Nansana Town Council and Busukuma Sub-Counties of Wakiso district.

5A.1.2 Study population

Target population: All adults 18 years and above living in Wakiso district.

Accessible population: All adults 18 years and above residing in the households of Nansana Town Council and Busukuma Sub-Counties.

Study population: Adults 18 years and above residing in the selected households of Nansana Town Council and Busukuma Sub-Counties that consent to participate in the study.

5A.1.3 Selection criteria

We will include all residents of Nansana Town Council and Busukuma Sub-Counties who are 18 years and above who have provided informed consent to participate in the study. We will exclude participant not available for meetings on 3 appointment dates.

5A.1.4 Sample size calculation

The formula: $\left\{ n = \frac{Z_\alpha{}^2(pq)}{d^2} \right\}$ x deff, will be used to determine the sample size, where p is the prevalence of hypertension and q is the complement of the prevalence. The tolerable error margin of the estimate is d, alpha is the level of significance and deff is the design effect to account for clustering effect. Using level of significance of 0.05, an error margin of 5% and a

design effect of 1.5, the adjusted sample requirement for an assumed 10% level of non-response (nr =10%) = N*

Based on previous cross sectional studies of non-communicable diseases and their risk factors in Kasese and Masaka districts in Uganda [28, 30] and in sub-Saharan Africa [15], as well as a meta-analysis for West African countries [155], hypertension was the most prevalent risk factor shown to vary from 4-22%.

Table 3 showing the required sample size for the survey

P	q(=1-p)	α	d	n	n x 1.5	N* (nr=10%)	Comment
0.04	0.96	0.05	0.05	59	89	98	
0.10	0.90	0.05	0.05	139	207	230	Meta-analysis, west African countries, Recent cross sectional study on NCDs in Kasese
0.18	0.82	0.05	0.05	227	340	378	Masaka MRC study
0.22	0.78	0.05	0.05	263	395	439	Cross-sectional study on CVD in SSA

The largest sample size is **440.**

Four hundred and forty participants will therefore be recruited from urban Nansana town council and 440 from rural Busukuma sub-county.

Since this research is a subset of the (MEPI-CVD) survey on prevalence and risk factors for cardiovascular diseases in urban and rural populations in Wakiso district in Uganda, we will utilise the data we will obtain from 5000 study participants.

5A.1.5 Sampling procedure

Participants for this survey will be selected using a multi stage sampling procedure at the sub-County, parish and village level. The enumeration units are the households. At the village level, a sampling frame of the household will be obtained from the census and households will be selected by systematic sampling. At the household level, one member out of all household members above 18 years will be randomly selected to participate. Those selected will be approached personally and requested for consent to participate into the study. Random selection of an eligible member of each household and request for consent to participate in the study will be done by a team of researchers every afternoon following an advance notice

of two days. Those that consent will be enrolled into the study and asked to attend a centrally placed research clinic/health centre the following morning after fasting overnight for at least eight hours. All those invited will be given numbered identity cards and transport to the centre will be reimbursed. If a person fails to turn up after their first invitation they will be visited up to three times either at home or at their place of work in order to find out the reason for failure to turn up and encouraging them to honour the invite. Participants who fail to turn up after the three invitations will be deemed non-responders.

Figure 4: Sampling & operational flow diagram for survey

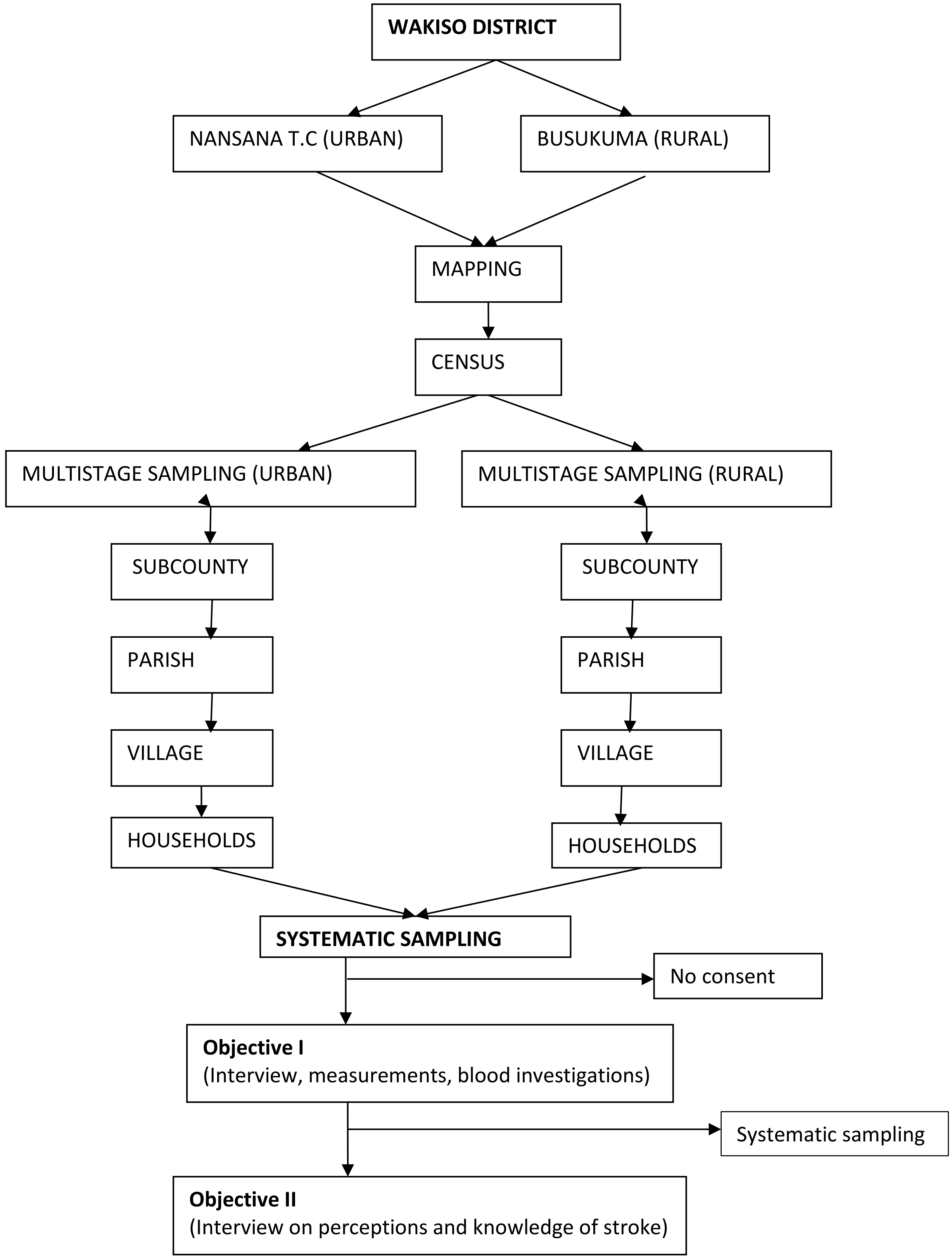

5A.1.6 Data Collection

Along with the PI and a specially trained survey team information required will be collected using a pre-coded and pre-tested standardised data collecting tool. This questionnaire will be administered by face to face interviews in a research clinic or a selected area ensuring a setting that provides maximum privacy to conduct the interview.

5A.1.7 Data collection tools

A pre-coded and pre-tested questionnaire consisting of four sections will be used as the data collecting tool. Electronic versions of the survey questionnaire will be created for personal digital assistants (PDA) using appropriate software. Survey teams will move in pairs; an interviewer who will administer the questionnaire and enter the data into the PDA and an assistant who will record answers on a paper questionnaire.

In **Section 1** of the questionnaire, participants will be asked to self report socio-demographic data including age, gender, marital status, tribe, religion, highest level of education attained.

Section 2 of the questionnaire will assess the prevalence of stroke risk factors in the study population. This section will be divided into three subsections as shown below.

1. <u>Self-reported stroke risk factors;</u>

- Present medical history for symptoms suggestive of a stroke namely sudden painless weakness, numbness or dead feeling on one side of the body, sudden inability to understand what other people are saying, and inability to express self verbally or in writing and sudden loss of one side of your vision, sudden severe headache that can’t be explained, convulsions and vomiting, loss of consciousness. The duration since onset of symptoms will also be obtained.
- Past medical history of stroke risk factors namely previous stroke/TIA, diabetes, hypertension, high blood cholesterol, heart disease (coronary heart disease and heart failure), obesity, cancer, sickle cell disease and HIV/AIDS. A physician’s diagnosis of the aforementioned medical conditions made during previous clinic visits or hospital admissions will be accepted.
- Lifestyle/social activity namely current smoking, alcohol intake, physical activity, will be obtained. Current smoking will be determined as the number of cigarettes smoked per day. This will be divided into 3 groups (5 to 15, 16 to 20, and >=21 cigarettes per day)Physical activity will be categorised into moderate activity, low activity and sedentary according to the International Physical Activity (IPA) score (Appendix 14)

Alcohol consumption will be based on CAGE system categorised as; I) no suspected alcohol problem, II) suspected alcohol problem, III) alcohol abuse/ dependency and broadly into number of drinks in a month (> or < than 30). (Appendix 15)

- Dietary habits regarding frequency of intake of fruits and vegetables as well as specific foods daily and weekly
- Family history of stroke, TIA, hypertension, diabetes, heart attack, heart disease, high body fat will be obtained.
- History of current use of drugs; antihypertensives e.g. captopril, atenolol, nifedipine, aprinox; antiplatelet agents such as aspirin, clopidogrel; anticoagulants such as warfarin, heparin; antithrombotic such as streptokinase; lipid lowering drugs such as atovastatin, fenfibrate, simvastatin will be sought. Use of oral contraceptives (women only): past and /or current use of birth control pills either for contraception or other indications. The past medical history will be reviewed for other medications and previous history of hospitalisation. Review of previous discharge summaries, results of previous laboratory tests, and prescriptions from previous clinic visits, will be reviewed in an attempt to obtain information not mentioned during the interview. The information will be presumed to be absent if we are unable to find clear documentation in the case where a participant will volunteer information that he or she is not sure about.

2. Blood pressure, Heart rate and rhythm and Anthropometric measurements

   The survey team will perform some simple measurements on the study participants. These will be done in the clinic using calibrated equipment. The following measures will be taken using standardized protocols and recorded:

- Blood pressure (Appendix 17)
- Height (Appendix 19)
- Weight (Appendix 19)

  Height and weight will be used to calculate the body mass index (BMI)
- Waist and hip circumference (Appendix 19)

  Waist and hip circumference will be used to calculate the waist-hip ratio

3. Collection of body fluids samples and laboratory studies

   The final stage of the survey will involve collection of blood samples from the participants in the morning hours after at least 8 hours of overnight fasting. A veni-puncture will be performed under aseptic conditions and in line with standard procedures using a 24 gauge needle and syringe to obtain about 10mls of blood.

This will be placed in varied amounts in appropriate vacutainer tubes and used to carry out the following tests:

i) Fasting lipids (LDL, HDL and triglycerides, total cholesterol): 4mls of blood will be dispensed into a yellow top with a gel separator vacutainer. Analysis will be done using Eon One semi-automated clinical chemistry analyser. The values obtained will be classified according to ATP III guidelines. **.**

ii) Rapid Plasma Reagin (RPR): 2 mls of blood will be dispensed into a red top vacutainer and left to clot to obtain serum which will be used to determine the reagin antibody titres using the syphilis RapiCard Insta test and RPR carbon if positive.

iii) HIV serology:2mls of blood will be dispensed into a red top vacutainer and left to clot to obtain serum that will be used to for HIV serology. Tests to detect HIV antibodies will be done using HIV rapid strips (Determine, Statpak and Unigold), as outlined in the national guidelines for HIV testing from the Ministry of Health. Pre and post test counselling for HIV will be done and participants with positive results will be referred for further management at established centres.

iv) Fasting blood glucose: Blood will be obtained by a prick at the tip of the patient's ring finger using a lancet. One to two drops of whole blood (10-20 microlitres) will be placed at the tip of a glucostick, which will then be placed into an On call plus glucometer for measuring fasting blood glucose.

Section 3 of the questionnaire will assess the participant's perceptions of stroke. They will be asked about the causes of stroke (demons, witchcraft, God's will, any of the risk factors, inheritance, fatty foods, angry ancestral spirits, don't know), organ of the body affected by stroke (brain, heart, liver/kidney, don't know), planned response to an event of stroke (call general practitioner or family doctor, ask family members or relatives to help, go to chemist for advice or medication, self medication, ask friend or neighbours for help, go to hospital, visit alternative health care providers (herbal med, traditional healers), seek spiritual healing (prayer), combination of hospital and tradition, combination of hospital and faith) , concern about possibility of having a stroke (none, low life time chance, moderate life time chance, high life time chance), concern about possibility of having a stroke in last twelve months (yes, no, never bothered about it, don't know), whether stroke is preventable (slightly, to some extent, totally), if stroke can be prevented if treated early, likelihood of stroke events (once, more than once, don't know), effect of stroke on day today activities like driving a car, dressing, use of the toilet and having a job.

Section 4 of the questionnaire will assess the participant's knowledge of stroke warning signs, knowledge of stroke risk factors, planned response to an event of stroke, concern about possibility of having a stroke and sources of stroke information. The stroke warning signs whose knowledge will be assessed will include dizziness, blurred or double vision or loss of vision, headache, sudden difficulty in speaking or understanding or reading, chest pain or chest tightness, nausea/vomiting, tiredness, fever/sweating, shortness of breath, numbness tingling sensation or dead sensation of any body part, numbness tingling sensation or dead sensation of any part/one side of the body, weakness of any part/one side of the body, paralysis of any part/one side of the body, fainting or black out or collapse and I don't know.

The participant's knowledge of stroke warning signs will be described based on the key below [10, 148]:

Good- knowledge of 5- 10 stroke warning signs

Fair- knowledge of 2-4 stroke warning signs

Poor- knowledge of at least one stroke warning sign

The stroke risk factors whose knowledge will be assessed will include old age, hypertension, diabetes, cigarette smoking, heart disease, alcohol, atherosclerosis, high cholesterol, obesity, genetics (hereditary), stress, lack of exercise, poor hygiene, headache or migraine, cancer, use of oral contraceptives, bad diet, tremors, others and I don't know.

The participant's knowledge of stroke risk factors will be described based on the key below:

Good- knowledge of 5- 10 stroke risk factors

Fair- knowledge of 2-4 stroke risk factors

Poor- knowledge of at least one stroke risk factors

Lastly the participants will be asked about the sources of information in regard to stroke (life experiences of friends, life experiences of family members, health personnel, electronic media; television, radio, newspaper, community meetings etc.).

5A.1.8 Data management

The data obtained will be checked for completeness at the end of each participant-researcher contact. Every evening, data will be downloaded to a Microsoft access database STATA version 12. Data captured using paper record forms will be used as back up if synchronisation of the PDA to the database fails. In such situations, data from paper record forms will be entered into the access database by the data entry clerk and will be double entered to ensure accuracy. Back-up files of the database will be kept at the end of each data entry session. Safety of all the data will be ensured by using laptops with a password known by only the study team members as well as storage in cupboards under lock and key. At the end of the day a meeting will be held to discuss the research progress, challenges and way forward.

5A.1.9 Data analysis

The data obtained will be organised and coded and then exported to SPSS version 12 software for analysis by the PI assisted by a statistician.

Analysis plan

Regarding Objective 1: Descriptive statistics of mean, frequencies and percentages will be used to summarise and present data on the participants' socio-demographic variables

The prevalence of known risk factors for stroke will be calculated as percentages, the total study sample being the denominator.

Logistic regression analysis will be used to identify the socio-behavioural characteristics associated with the three most prevalent known stroke risk factors. Only socio-behavioural factors that are significantly associated with stroke risk factors at bi-variable analysis ($P<0.05$) will be included in the adjusted model.

Regarding objective 2: Descriptive statistics of mean, frequencies and percentages will be used to summarise and present data on the participants' socio-demographic variables

Descriptive statistics of mean, frequencies and percentages will be used to summarise and present data on the participants' stroke related knowledge and perceptions (organ involved, knowledge of at least one or more established stroke risk factors and warning signs, knowledge of stroke preventions, treatment choice among others).

Bivariate analysis will be used to test the associations between components of stroke related knowledge (organ affected by stroke, ≥ 5 risk factors and ≥ 5 warning symptoms of stroke)

and socio-demographic characteristics, planned response to an event of stroke and self-reported stroke risk factors. All tests of hypothesis will be two tailed with a type 1 error set at 0.05. Factors significant at bivariate analysis will be subjected to multivariate analysis using multivariable logistic regression model.

5B METHODOLOGY FOR AIM II

5B.1 Study design

This will be a non-randomised controlled trial (quasi experimental study)

5B.2 Study site

The study will be carried out on the Accident and Emergency unit, Neurology (medical and surgical) wards and the general intensive care unit (3D) at Mulago hospital. Mulago, Uganda's national referral hospital and Makerere University College of Health Sciences' teaching hospital has a bed capacity of 1,500 beds. It has an Accident and Emergency unit where all patients with suspected stroke present. On average this unit receives 25 to 30 patients with suspected stroke per month. The hospital also has a Radiology department with a Computerised Tomography (CT) scan and other imaging facilities, adequacy of laboratory and other supportive services as well as well trained manpower such as radiologists, cardiologists and neurosurgeons to help interprete findings of imaging investigations and provide surgical services to the patients.

5B.3 Study population

Target population: All adult patients with stroke in Uganda

Accessible population: Adult patients that present to Mulago hospital with stroke

Study unit: All patients 18 years and above with stroke presenting to the Accident and Emergency unit during the study period and meet the inclusion criteria.

5B.4 Selection criteria

<u>Inclusion Criteria:</u>

- Aged 18 years and above
- Stroke confirmed by CT scan of the brain.
- Patient and/or attendant give informed consent to participate in the study.

<u>Exclusion criteria:</u>

- Patients unable to communicate because of severe stroke, aphasia, or dementia without a valid surrogate respondent (Is considered a spouse or first degree relative that is living in the same home or is self-identified as aware of the participant's previous medical history and current therapies)
- Patients presenting after 7 days since onset of stroke

5B.5 Sample size calculation

The study will involve 2 groups of stroke patients. One group (P2) will participate in the pre-intervention stage estimated to take about 6 months and the second group (P1) will participate in the intervention stage that will follow immediately after. The intervention stage is also estimated to take about the same time.

Using PASS 2008 software for two independent proportions (null case) power analysis (Chow SC et al 2003, Fleiss JL et al 2003, Lachin JM 2000, Machin D 1997), the sample size for the two groups will be calculated as follows

Numeric Results of Tests Based on the Difference: P1 - P2

H0: P1-P2>=0. H1: P1-P2=D1<0. Test Statistic: Z test with pooled variance

H0 is the null hypothesis

H1 is the alternative hypothesis

'P1' is the proportion for group one under H1. This is the treatment or experimental group. Effect of implementing the stroke bundle is expected to reduce mortality by 15% [72], therefore P1= 28.8%

'P2' is the proportion for group two. This is the standard, reference, or control group. Un published study done in 2009 to 2010 by Kwarisima L et al, reported 30 day mortality among adult stroke patients admitted at Mulago hospital of 43.8% [6]

Power	Sample Size Grp 1 N1	Sample Size Grp 2 N2	Prop|H1 Grp 1 or Trtmnt P1	Prop Grp 2 or Control P2	Diff if H0 D0	Diff if H1 D1	Target Alpha	Actual Alpha	Beta
0.8027	127	127	0.2880	0.4380	0.0000	-0.1500	0.0500		0.1973

Note: exact results based on the binomial were only calculated when both N1 and N2 were less than 100.

'Power' is the probability of rejecting a false null hypothesis. It should be close to one.

'N1 and N2' are the sizes of the samples drawn from the corresponding populations.

'Target Alpha' is the probability of rejecting a true null hypothesis that was desired.

'Actual Alpha' is the value of alpha that is actually achieved.

'Beta' is the probability of accepting a false null hypothesis.

Summary Statements

Group sample sizes of 127 in group one and 127 in group two, achieve 80% power to detect a difference between the group proportions of -0.1500. The proportion in group one (the

treatment group) is assumed to be 0.4380 under the null hypothesis and 0.2880 under the alternative hypothesis. The proportion in group two (the control group) is 0.43800. The test statistic used is the one-sided Z test with pooled variance. The significance level of the test was targeted at 0.0500. The significance level actually achieved by this design is NA.

5B.6 Sampling method and recruitment

Pre-intervention group

- Study participants who meet the inclusion criteria will be selected by consecutive sampling until the sample size of 127 is attained.
- The principal investigator (PI) and trained research assistants will recruit patients from Monday to Sunday of each week. They will be called to the Accident and Emergency unit (resuscitation room and emergency wards) by clinicians serving in this unit, each time they receive an adult ($\geq$18 years) patient with suspected stroke, whose symptoms have lasted $\leq$7 days. The PI and research assistants will take note of these patients, talk to them and or their attendants about the study, including aims, objectives, benefits and risks. They will attend to their questions and then request them to participate in the study. Patients with suspected stroke who will give written informed consent or their attendant will have an audit of the elements of the stroke care bundle resume at this time. The "Emergency department stroke and transient ischemic attack care bundle" or "Stroke care bundle" is a tool adopted from the Australian government National Health and Medical Research Council [73]. If implemented from the time a patient with suspected acute stroke presents, it takes 72 hours. Elements of the stroke care bundle that will be audited will include the rapid initial stroke screen using the validated ROSIER scale, $ABCD^2$ assessment when TIA is suspected, CT scan of the brain in under 24 hours of admission, Nil by mouth until bedside swallow screen is done within 24 hours, Aspirin within 48 hours if haemorrhagic stroke is excluded, Physiologic monitoring and management of neurological status, blood glucose, BP, hydration status and temperature. They will mark as Y (yes) the elements of the bundle that will have been applied, N/A- for the elements that will not apply to the patient, CI- for the elements that are contraindicated in the patient and lastly N (no)- for the elements that will not have been applied.

- Following CT scan of the brain, all patients with confirmed stroke, eligible to participate in the study and have none of the exclusion critteria will be enrolled into the study consecutively until the sample of 127 is attained.
- Patients with suspected stroke, in whom CT scan of the brain will reveal pathology other than vascular as responsible for the symptoms (stroke mimics), will not be eligible to participate in the study. The attending team will continue with patient management.
- A patient whose results of the initial CT scan of the brain are reported as normal (done in less than seven days since onset of neurological deficits) will be followed up for the results of a repeat CT scan on the 7th day. If the repeat CT scan results are also reported as normal, the patient will not be eligible to participate in the study. The attending team will continue with patient management.
- The principal investigator and research assistants will then take a detailed clinical history from the patient or the attendant (The first choice of proxy will be the spouse, live in companion, followed by a daughter/son ($\geq$18 years), parent, sibling, or close friend of the patient).
- Information sought after will include selected socio-demographic characteristics; present medical history of stroke; past medical history of known stroke associated risk factors; and history of past and current medications namely anti-hypertensive, anticoagulants, diabetes mellitus glycemic control drugs, lipid lowering drugs and antiplatelet agents.
- Physical examination will include baseline physiological monitoring: temperature, neurologic status, blood pressure, finger stick glucose and hydration status. Neurological examination for level of consciousness, and patterns of presenting signs, severity of stroke using the Scandinavian stroke scale, cardiovascular examination including pulse rate and rhythm, apex beat, carotid bruits. A bedside swallow evaluation will also be done.
- Laboratory investigations will be done on blood samples obtained every morning after patients have fasted for at least 8 hours over night. Investigations done will include fasting lipid profile, fasting blood sugar, RPR, CBC and ESR. HIV serostatus of patients will be obtained from the HIV routine counselling and testing programme currently running on the emergency wards.
- CT scan of the brain other than confirm stroke, will be used to determine the stroke subtypes and exclude stroke mimics.

- Imaging investigations will include CXR for patients with fever, 12 lead ECG, echocardiography and doppler ultrasound of the carotid arteries among patients with abnormal cardiovascular examination.
- Observation for physiologic monitoring of temperature, blood pressure, blood sugar and hydration status will be done 4 to 6 hourly till 72 hours post stroke confirmation.
- Patients will be followed up for a period of 30 days from admission to determine the length of stay in hospital and 30 day outcomes (mortality, disability and activities of daily living).

Intervention group

- The sampling method and recruitment will be done as in the pre-intervention study. However unlike in the pre-intervention study where stroke care bundle elements will be audited, they will be implemented in this group.

5B.7 Study measurements

Socio-demographics

- Socio-demographic characteristics will include age, sex, tribe, religion, marital status, highest level of education attained, next of kin and occupation.

Present medical history

- History of date of symptom onset and time in order to obtain the duration since symptom onset, symptoms suggestive of a stroke namely sudden onset (or on awakening from sleep) of painless weakness, numbness or dead feeling on one side of the body (face, arm, leg), sudden inability to understand what other people are saying, and inability to express self verbally or in writing and sudden loss of one side of your vision. Others will include sudden severe headache that couldn’t be explained, loss of consciousness, convulsions, vomiting.

Past medical history

- History of risk factors for stroke, namely previous stroke/TIA, diabetes, hypertension, high blood cholesterol, heart disease (coronary heart disease and heart failure) and HIV/AIDS. A physician’s diagnosis of the aforementioned medical conditions made during previous clinic visits or hospital admissions will be accepted.

Modified versions of validated questionnaires “The stroke risk assessment form” and “Questionnaire for verifying stroke/TIA free status (QVSFS)” adopted from the American Stroke Association 2003and recently updated and validated respectively will be incorporated into the data-collecting tool**.**

Lifestyle/social activities namely current smoking, alcohol intake, physical activity, will be obtained.

Current smoking will be determined as the number of cigarettes smoked per day. This will be divided into 3 groups 1) 5 to 15, 2) 16 to 20, and 3) 21 or more.

Leisure- time physical activity will be categorised according to the physical activities questionnaire [156] into three subcategories according to the exercise frequency as 1) “never/rarely”, 2) “1 to 2 times per week,” and 3) “3 or more times per week” (Appendix 14**)**

Alcohol consumption will be based on CAGE system [157] (Appendix 15) categorised as; 1) no suspected alcohol problem, 2) suspected alcohol problem, 3) alcohol abuse/ dependency.

- Family history of stroke, TIA, hypertension, diabetes, heart attack, heart disease, high body fat.
- History of current use of drugs to control blood pressure such as calcium channel blockers, ACEI, ARBs; control blood sugar such as glibenclamide, metformin, thiazolidindiones ; antiplatelet agents such as aspirin, clopidogrel; anticoagulants such as warfarin, heparin; antithrombotic such as streptokinase; lipid lowering drugs such as atovastatin, fenfibrate, before admission will be sought. Past and /or current use of birth control pills among women, either for contraception or other indications.

The past medical history will be reviewed for other medications and previous history of hospitalisation. Review of previous discharge summaries, results of previous laboratory tests, and prescriptions from previous clinic visits, will be reviewed in an attempt to obtain information not mentioned during the interview. The information will be presumed to be absent if we are unable to find clear documentation in the case where an attendant volunteers information that he or she is not sure about.

Physical examination.

- Temperature will be measured using a digital thermometer placed into the axilla of the patient. It will be removed when it beeps and the reading displayed will be taken.

- The radial pulses will be examined for irregularity of the rhythm by finger palpation, and the heart will be auscultated for irregularity of the heartbeat using the diaphragm of a stethoscope applied to the apex.
- Carotid artery bruits will be examined for, by placement of the diaphragm of the stethoscope over the carotid arteries.
- Blood pressure will be measured using Omron M6 automatic blood pressure monitors. Blood pressure will be measured two times in the lying position (most of the patients are unable to sit up) at an interval of five minutes and an average of the two measurements obtained. This will be classified according to the National Clinical Guideline Centre (NCGC) clinical guideline 127 [158].(Appendix 18) with hypertension classified as SBP$\geq$140mmHg and DBP $\geq$ 90mmHg**.** Diagnosis of hypertension will be made in case of an established diagnosis before onset of the stroke or target organ damage otherwise hypertensive readings on admission may be a reflection of stroke reactive hypertension.
- Waist circumference will be measured to the nearest centimeter, using a soft unstretchable tape measure midway between the lowest rib and the iliac crest at the end of a normal expiration. It will be held snug but not compressing the skin.
- Hip circumference will be measured to the nearest centimeter using an unstretchable tape measure in the horizontal plane over the widest part of the gluteal region.
- Waist to hip ratio will be obtained by dividing waist by hip circumference.
- The nervous system will be examined for alteration in level of consciousness and pattern of stroke presentation. This will involve assessment of the motor system for presence of expressive aphasia, hemiparesis, quadriparesis, facial weakness, gaze deviations, dysathria and dysphagia, muscle power, tone and reflexes.

  The sensory system will be assessed for signs of hemianaesthesia or quadrianaesthesia, receptive aphasia. Visual fields will be assessed crudely for defects such as hemianopia, hemi-inattention, and diplopia/dysconjugate gaze. Stroke severity will be determined using the Scandinavian Stroke Scale (SSS) [159] (Appendix 20). In case of unclear signs, a consultant neurologist will be requested to re-examine the patient.

Investigations

- **Computerised Tomography (CT) scan.**

  A plain CT scan of the brain will be used to confirm stroke in the study patients. A Philips MX 16 slice CT scanner situated at Mulago Hospital’s department of radiology

will be used. Interpretation of the CT scan findings will be done with assistance from Senior Consultant Radiologists.

Cerebral infarction:

A low-density lesion on CT scan that conforms to a vascular territory compatible with the clinical presentation.

Ischemic stroke subtypes will be classified according to the Trial of ORG 10172 in acute stroke treatment (TOAST) as atherosclerotic, cardioembolic lacunar or other etiology [160](Appendix 16)

Cerebral haemorrhage:

High-density areas of haematoma on CT scan without contrast enhancement.

it will be classified according to medical disability guidelines for definition of heamorrhagic stroke on non-contrast CT scan as intraparenchymal haemorrhage if high density lesion is in the brain parenchyma or Subarachnoid hemorrhage (SAH) if high-density lesion is in the subarachnoid space [161]**.**

- **12 lead Electrocardiography**

  All patients will have a 12 lead ECG done using CARDIOVIT AT-102 machine. This will be done to diagnose atrial fibrillation and electrocardiographic manifestations of ischemic heart disease.

- **Echocardiography**

  Transthoracic echocardiography will be done using the Phillips HD7 XE machine situated on the cardiology ward. Cardiologists will look for cardio-embolic sources of stroke such as akinetic wall segments, mural thrombi, severe valvular lesions, multiple valvular lesions, valvular vegetations and ventricular ejection fraction of less than 35% in patients with abnormal cardiovascular examination.

- **Doppler ultrasound scans of the carotid arteries.**

  Doppler ultrasound scan of the carotid arteries will done using the Phillips HD7 XE machine. Cardiologists will look for atherosclerotic plaques, discrete lesions, and

abnormal velocity flow patterns as evidenced by turbulence, irregular flow peaks, and evidence of carotid artery stenosis.

Laboratory investigations

The study subjects will be required to fast for at least eight hours overnight and the blood will be drawn in the morning before breakfast.

Blood

- Using aseptic technique, a 24-gauge needle with a 10ml syringe will be used to draw 6mls of blood from a peripheral vein of each study patient.
- 3mls of blood will be put in a sequestrine bottle for determination of the CBC by coulter counter machine and the ESR by Westergren method.
- The remaining 3mls will be put into a sterile plain vacutainer tube for measuring fasting serum lipids and RPR from serum. The lipid/cholesterol assay will be done by using Eon One semi-automated clinical chemistry analyser. Values obtained for the lipid profile will be classified according to ATPIII criteria of classification of lipids. The RPR test for syphilis will involve 2 tests. Test 1 will be by the Syphilis Rapicard Insta test. All positives by this test will then be subjected to a second test, the RPR carbon.
- Results of the patient's HIV serostatus will be obtained from the HIV Routine Counselling and Testing programme currently operating on the emergency and medical wards for the hospital study. HIV test for the survey participants will be done using the serial algorithm (3tests) as per the Ministry of Health guidelines. Test 1 will determine HIV1/2(screening). All positives will be subjected to the STST PAK for confirmation. UNIGOLD will be the 3rd test (tie breaker).
- Blood will be obtained by a prick at the tip of the patient's ring finger using a lancet. One to two drops of whole blood (10-20 microlitres) will be placed at the tip of a glucostick, which will then be placed into the On call plus glucometer for measuring fasting blood glucose.

5B.8 Thirty day follow up

- Patients will be reviewed and followed up to 72 hours from admission to observe physiological monitoring of temperature, blood pressure, blood glucose and hydration status in order to complete auditing of the stroke care bundle. There after they will be

followed up every after 2 days from the day of admission while on the ward and at discharge to determine length of hospital stay of both in-hospital deaths and the non-fatal cases. In case of the intervention stage, patients will be reviewed and followed up to 72 hours from admission to implement the elements of the stroke care bundle. Thereafter, they will also be followed up every after 2 days while on the ward and at discharge to determine length of hospital stay of both in-hospital deaths and the non-fatal cases. Similar to the pre-intervention stage, at discharge, nonfatal participants will be provided with a review date in the Neurology out patients clinic to coincide with the 30th day since admission. Notification will be given a week prior to the review date and telephone contact with the patient will be maintained every week post discharge. A patient’s contact details (patient’s telephone number and physical address and telephone contacts of at least 2 next of kin and their physical address) will be obtained at the time of enrolment in order to make contact post-discharge possible. In case of death within 30 days from admission, the date of the eventual death and the suspected cause will be recorded. The 30th day review for participants who will have survived will be done either in the neuro-outpatient clinic, contacting the family for a personal visit to their home or by telephone enquiry in order to determine the outcomes of disability as well as activities of daily living. Disability status at 30 days will be assessed using the modified Rankin scale (MRS) [162]. MRS measures performance of specific tasks. The scale consists of six grades from 0 to 5; 0 denotes no symptoms and 5 indicates severe disability (Appendix 21). For clinical purposes, mild disability will range from 0 to 2; moderate disability (3 to 4) and 5 will indicate severe disability. Barthel index (BI) [163] will be used to measure the patient’s performance in activities of daily living over 10 domains (feeding, bathing, grooming, dressing, bowels, bladder, toilet use, transfers, mobility and stairs) after summing the scores from each domain to provide a total score (range 0-100). A score greater than or equal to 95 will be indicative of independence (Appendix 22).

Figure 5. Sampling and operational flow chart for Study III (Pre-intervention stage)

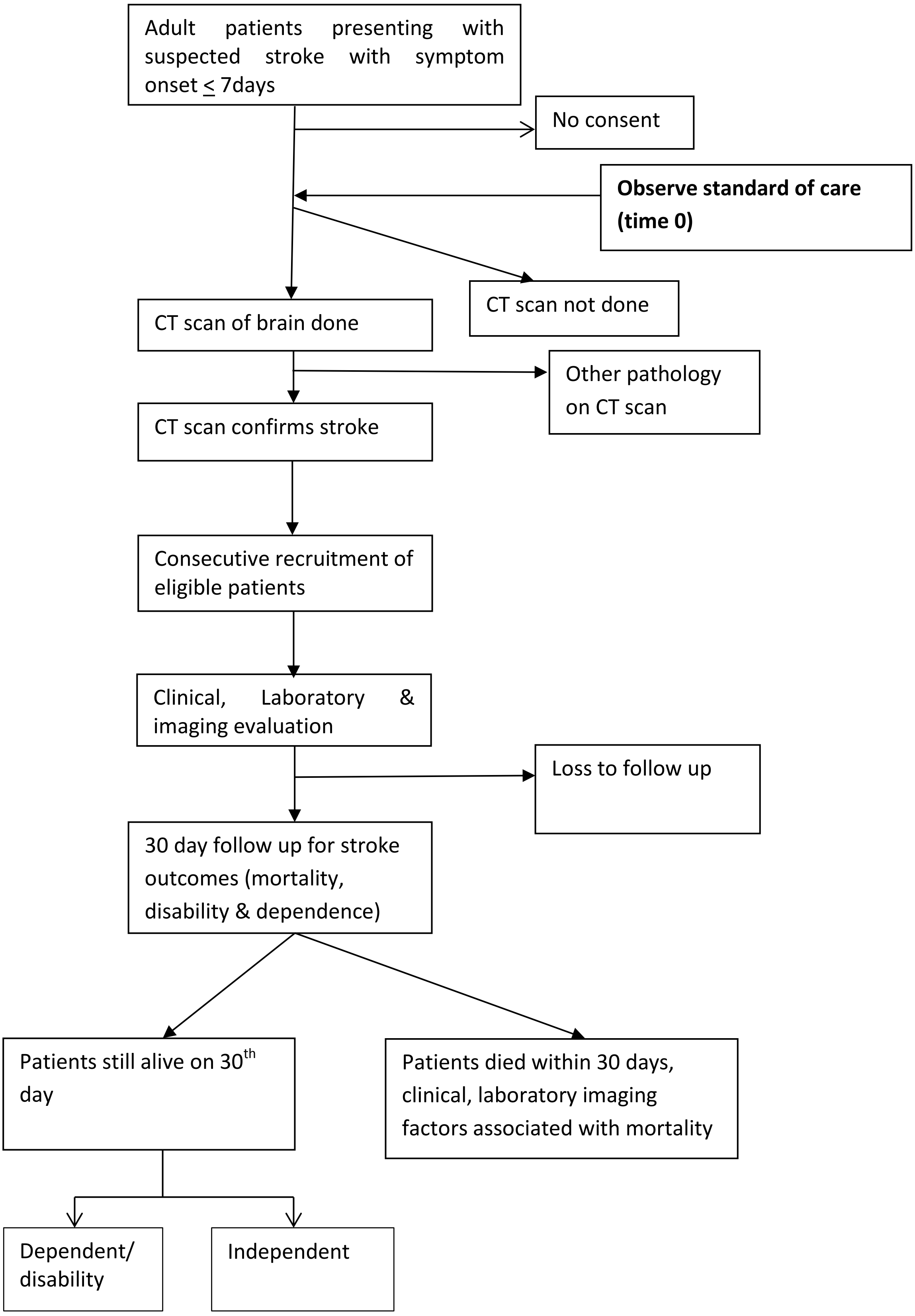

Figure 6. Sampling and operational flow chart for Study Aim I (Intervention stage)

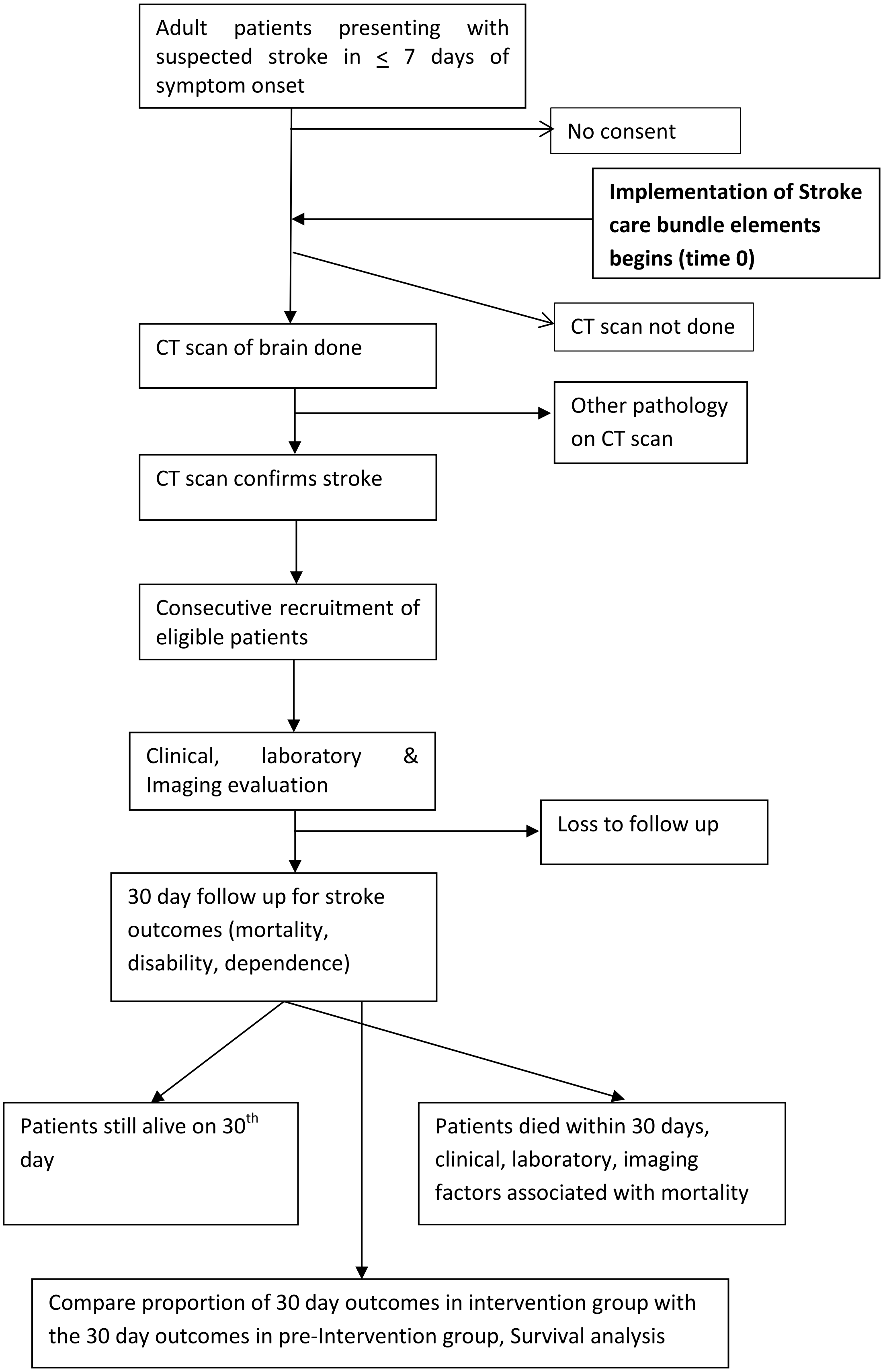

5B.9 Data collection

Along with the PI and 2 specially trained medical research assistants who work as doctors at Mulago hospital, doctors, neurologists and physicians (internal medicine), neurosurgeons, radiologists, cardiologists, nurses, social workers, physiotherapists, and a speech therapist working in the study area will cooperate in the study that will take place at Mulago hospital as stated below

Doctors: They will participate in notifying the research team about patients presenting to the emergency unit with suspected stroke. They will also take care of these patients as per the standard of care.

Neurologists and internal medicine physicians: They will take care of the stroke patients admitted on the neurology ward as well as participate in decision making regarding further management of patients presenting with stroke mimics.

Neurosurgeons: Following results of CT scan of the brain, they will be responsible for carrying out indicated surgical interventions.

Radiologists: They will participate in the final interpretation of the CT scans of the brain and chest x-ray films.

Cardiologists: They will participate in the final interpretation of echocardiography, doppler ultrasound of the carotid arteries imaging and 12 lead ECG findings.

Nurses: They will take part in notifying the research team about patients presenting to the Accident and Emergency unit with suspected stroke. They will also take care of the patients in the emergency unit, the neuro- medical and surgical wards as per the standard of care.

Social workers and physiotherapists: They will participate in the care of patients as per the standard of care

Speech therapist: She will carry out swallow evaluation assessment of all patients who will have failed the swallow evaluation test carried out by the PI and research assistants.

The medical research assistants will receive intensive training on definitions/terminologies in respect to pre-coded and pre-tested standardised data collecting tools and responses, including independent assessment of neurological deficits at admission.

5B.9.1 Data collection tools

The PI and trained research assistants will collect data, using pre-coded and pre-tested data collecting tools as shown in the appendices listed below. Relevant information will be gathered and the variables to be measured will include selected socio-demographic

characteristics, an audit of elements of the stroke care bundle (Appendix 1) rapid initial stroke screen using the validated ROSIER scale (Appendix 3), $ABCD^2$ assessment when TIA is suspected (Appendix 5), CT scan of the brain in under 24 hours of admission, Nil by mouth until bedside swallow screen (Appendix 6) is done within 24 hours, Aspirin within 48 hours if haemorrhagic stroke is excluded, Physiologic monitoring and management of neurological status, blood glucose, BP, hydration status, temperature) (Appendix 7), clinical characteristics (present and past medical history of stroke and associated risk factors, Physical examination for temperature, BP, heart rate, pulse rate, rhythm and clinical features of stroke, stroke severity and stroke associated risk factors), laboratory characteristics (fasting lipid profile, RPR, CBC and ESR, fasting blood sugar, HIV serostatus) and imaging (CXR will be done among febrile participants and 12 lead ECG, echocardiography and doppler ultrasound of the carotid arteries will be done among participants with abnormal cardiovascular examination). Other than confirm a stroke, CT scan of the brain will be used to determine the stroke subtypes. Ischemic strokes will be classified according to the Trial of ORG 10172 in Acute Stroke Treatment or TOAST as, atherosclerotic, cardio-embolic, or other etiology to include lacunar and unspecified stroke subtypes [160]**.** Hemorrhagic strokes will be classified according to medical disability guidelines for definition of hemorrhagic stroke on non-contrast CT scan as intraparenchymal hemorrhage or subarachnoid hemorrhage [161]**.**

5B.9.2 Data collection method

Data will be collected by the PI and trained medical research assistants during face to face interviews with the study participants or valid surrogates, by physical examination of the study participants and from results of imaging and body sample investigations. Telephonic responses of study participants who will fail to turn up for the 30 day follow up to the neurology clinic for review and cannot be followed up at home will also be obtained. During the pre-intervention stage, the stroke bundle audit data will be obtained by on-going chart audits. Sources of data will include the medical records file (emergency unit documentation, medication record, radiology documentation, speech therapy documentation), radiology data base and speech therapy data base. All the information obtained will be filled in a pre-coded and pre-tested data collection tools. The data obtained will be checked for completeness at the end of each participant-researcher contact. At the end of each day, a meeting will be held to discuss the research progress, challenges and way forward.

5B.9.3 Adverse events reporting

Regarding the intervention stage of the study, all observed or volunteered adverse events regardless of suspected causal relationship to the intervention (stroke care bundle) shall be reported. Adequate information will be obtained to determine the outcome of the adverse event (AE) and to assess whether it meets the criteria for classification as serious adverse event (SAE). Notification of the PI about the event will be done and then reported to the local IRB and Data and Safety Monitoring Board (DSMB). The PI will assess the SAE causal relationship to the study interventions, follow up until the event or sequela has resolved or stabilised at a level acceptable to the IRB and DSMB.

Identification of adverse events

At follow up during hospitalisation, at any unscheduled hospital visit after discharge and at 30 days post stroke, study clinicians will assess the participants according to a standardised operating procedure (SOP). After discharge and before day 30, self-reporting will be used. A severity grading scale, based on toxicity grading scales developed by the WHO and the National Institutes of Health, Division of Microbiology and Infectious Diseases, will be used to grade severity of all symptoms and physical examination findings. Any new event, or/ and event present at baseline that is increasing in severity, will be considered an adverse event.

Reporting of serious adverse events

For each adverse event identified and graded as moderate, severe or life threatening, a serious adverse event report form will be completed (Appendix 23)

The following information will be recorded for all adverse experiences that are reported:

Description of event, date of event onset, date event reported, severity of the event, relationship of the event to study medication, is the event serious, initials of the person reporting the event, nature of the event, outcome, date event resolved.

The adverse events will be documented and submitted to the IRB with the annual report while the serious adverse events will be reported in 24 hours and then full report within 5 working days. Guidelines for reporting of serious adverse events provided by the Makerere University School of Medicine Research and Ethics Committee, the Uganda national Council for Science and Technology, and the DSMB will be followed.

Data and safety monitoring board

A data and safety monitoring board will be assembled in conjunction with Makerere university College of health Sciences, consisting of a minimum of a chairman, a safety monitor, a clinical monitor and a statistician. They will review interim analyses of data and oversee evaluation of emerging safety data on an on-going basis.

Stopping guidelines

Interpretation of results and decisions about discontinuation of the study will be made by the members of the DSMB. Stopping guidelines will be outlined in detail in the DSMB shell report, and will be based on the primary outcome and the incidence of the serious adverse events.

5B.10 Data management and analysis

The data obtained will be organised and coded. Raw data will be stored to prevent loss. It will then be double entered using STATA version 12 and then exported to SPSS version 15.0 software for analysis by the PI assisted by a statistician.

Analysis plan

Description of the socio-demographic characteristics of the study participants will be described. Categorical variables will be summarised using frequencies and percentages and the results presented as tables. Continuous variables will be summarised using means (standard deviations) and medians (interquartile ranges).

Objective 1

To observe standard of care with regard to elements of the “Stroke care bundle” among patients presenting with stroke to Mulago hospital

Analysis of data obtained from fully filled observation forms of the 127 pre-intervention stage participants. Performance of each stroke bundle element and the level of bundle completion (none of the elements is audited as no “N’’) will be summarised using frequencies and percentages. The results will be presented as bar charts.

To determine the 30 day outcomes (mortality, disability and activities of daily living) and associated factors (socio-demographics, clinical and laboratory presentations) among adult stroke patients presenting with stroke at Mulago hospital

The study population will be categorised into participants who died within 30 days and those who were still alive at the end of the study period, the degree of disability (mild, moderate,

severe) and the performance in activities of daily living (independent, dependent). The data obtained will be summarised using frequencies and percentages and the results presented as tables and bar charts. Bivariate analysis using the Chi square test will be used to test the association between each outcome and [categorized] social demographic characteristics and clinical and lab characteristics. Simple logistic regression will also be used for bivariate analysis. A p-value will be set at the 5% level for all analyses and two sided tests of significance will be used.

1. Outcome of mortality at 30 days (alive, dead)
    - Socio-demographic characteristics against mortality
    - Clinical, laboratory and imaging characteristics against mortality
1. Outcome of ADL at 30 days (independent, dependent)
    - Socio-demographic characteristics against dependence
    - Clinical, laboratory and imaging characteristics against dependence
2. Outcome of disability at 30 days (mild, moderate, severe)
    - Socio-demographic characteristics against moderate to severe disability
    - Clinical, laboratory and imaging characteristics against moderate to severe disability

Factors significantly associated with the 30 day outcomes at bivariate analysis, will be subjected to multivariate analysis using multivariable logistic regression.

The above analysis will be done for both study participants in the pre-intervention stage and the intervention stage.

<u>Objective 2</u>

To determine the effect of implementing the “Stroke care bundle” on 30 day outcomes among adult stroke patients presenting with stroke at Mulago hospital.

Hypothesis: There is a reduction in 30 day outcomes of mortality of 15% among patients presenting with acute stroke when a “Stroke care bundle” is implemented.

- The 30 day outcome measures are categorical data, 2 independent groups (pre-intervention stage and the intervention stage) outcome proportions are being compared. Therefore the chi-square statistic will be used.
- The confidence level will be set at 95% ($\alpha=0.05$)
- One tailed testing will be used and the level of significance will be set at $p<0.05$

Survival analysis

- Kaplan Meier estimates of survivor functions of both groups

- Log rank test to test equality of survival function across groups
- Cox regression model

Safety analysis will also be done for the intervention stage. Adverse events will be collected for each study participant during this stage and safety data summarised in tables. Each adverse event will be counted once according to the date of onset. During the intervention stage data collection, interim analysis will be performed at 50% of the study sample data.

5.11 Quality assurance.

The internal validity will be ensured throughout the study by

- Training research team members in effective communication with the study participants
- Training specific to tasks that different members of the research team will perform such as interviewing techniques, physical measurements, use of PDAs, completing the questionnaires will be provided in order to collect high quality data
- Use of written standard operating procedures (SOPs) for the project activities as well as providing all the necessary project documents
- Use of validated equipment that will be standardised before use to carry out the physical measurements.
- Pilot testing the study instruments for clarity and duly standardised
- Cross checking the questionnaires to ensure completeness before the investigators leave the study site as well as holding brief meetings at the end of each day to ensure that good quality data is collected.
- Proper labelling and handling of specimen that are in turn analysed at a competent laboratory that has handled similar research projects before
- Use of specialised personnel as per protocol to carry out specialist investigations as well as assist in their interpretation.
- Ensuring that query programs are written into the database to limit the entry of incorrect data and ensure entry of data into the required fields.

CHAPTER SIX

6.0 PROTECTION OF HUMAN SUBJECTS

Ethical consideration

6.1 Institutional Review Boards

To undertake the proposed study will require approval by all the relevant Institutional Review Boards (IRB) which will include: 1) The School of Medicine Research and Ethics Committee (SOMREC) of Makerere University College of Health Sciences, 2) Mulago National Referral Hospital's Research and Ethics Committee (MREC), 3) The Uganda National Council for Science and Technology (UNCST). Any amendments or modification to the submitted protocol and related information sheets will be reviewed prior to project implementation.

6.2 Informed consent

Approval from concerned authorities will be sought before activities are commenced in the selected study areas. All participants will be requested to give written informed consent either by signature or thumb print prior to participating in the study activities. In case of a study activity involving patients with altered level of consciousness or who are unable to talk, the first choice of proxy will be the spouse, live in companion, followed by a daughter/son ($\geq$18 years), parent, sibling, or close friend of the person. Consent forms will be provided in a simple and concise language both in English and Luganda, describing the purpose of the study, potential risks and benefits. In case the need arises, a translator and an impartial witness in case of those that cannot read will be provided. All study participants will be ensured of confidentiality of the privileged information that will be collected.

6.3 Risks and Discomforts

This is a minimum risk study in which participants will experience some discomfort due to the needle prick while withdrawing blood. Participation may lead to loss of privacy just as occurs in any kind of research. However, all information gathered during this study will be treated as private by the study staff and the records will be kept securely in locked filing cabinets. No personal information that can link results to their owners such as names or telephone contacts will be used in any reports arising out of the study.

6.4 Benefits to the study subjects

A comprehensive stroke risk factor assessment useful in holistic patient management will be

done at no cost to the survey participants and results of the investigation will be returned to them. In case of abnormal findings, guidance on treatment, care or follow up will be provided by the survey team doctors.

In regard to the in-hospital study, investigations will also be done at no cost to the patients. Results will be availed to the attending teams for continued patient management.

6.5 Study limitations

Study III will utilise a non-randomised controlled study (Quasi-experimental study)

A randomised controlled trial will not be possible due to the fact that elements of the stroke care bundle have been proved through evidence based research to improve outcomes post stroke. It would therefore be deemed unethical to withhold them.

There is also lack of documented standard of care in the most recently released Uganda Clinical guidelines 2012.

Chart reviews or use of an existing historical control (about 4 years) were also considered however the reviewed data revealed a lot of missing information of interest as well as lack of multiple measurement points of interest for the study. We however acknowledge that due to lack of randomisation, temporal and or seasonal confounders may be present.

References

1. Feigin, V.L., et al., *Stroke epidemiology: a review of population-based studies of incidence, prevalence, and case-fatality in the late 20th century.* Lancet Neurol, 2003. **2**(1): p. 43-53.
2. Dalal, P.M., et al., *Population-based stroke survey in Mumbai, India: incidence and 28-day case fatality.* Neuroepidemiology, 2008. **31**(4): p. 254-61.
3. Dalal, P.M. and M. Bhattacharjee, *Stroke epidemic in India: hypertension-stroke control programme is urgently needed.* J Assoc Physicians India, 2007. **55**: p. 689-91.
4. Gupta, R., et al., *Epidemiology and causation of coronary heart disease and stroke in India.* Heart, 2008. **94**(1): p. 16-26.
5. Srindharan, S. and e. al., *Incidence, types, risk factors and outcome of stroke in a developing country: the Trivadrum Stroke Registry.* Stroke, 2009. **40**: p. 1212-18.
6. Kwarisiima, L., *Thirty day mortality, clinical and laboratory presentation among adult patients with stroke admitted at Mulago hospital* 2011.
7. Jones, S.P., et al., *Stroke knowledge and awareness: an integrative review of the evidence.* Age Ageing, 2010. **39**(1): p. 11-22.
8. Stroebele, N., et al., *Knowledge of risk factors, and warning signs of stroke: a systematic review from a gender perspective.* Int J Stroke, 2011. **6**(1): p. 60-6.
9. *Organised inpatient (stroke unit) care for stroke.* Cochrane Database Syst Rev, 2007(4): p. CD000197.
10. Ayanniyi, O., O. Akande, and A.F. Mustapha, *Knowledge and perception of stroke among adults in Osogbo, Nigeria.* Afr J Med Med Sci, 2006. **35**(4): p. 447-52.
11. Yoon, S.S. and J. Byles, *Perceptions of stroke in the general public and patients with stroke: a qualitative study.* BMJ, 2002. **324**(7345): p. 1065-8.
12. Mshana, G., et al., *Urban-rural contrasts in explanatory models and treatment-seeking behaviours for stroke in Tanzania.* J Biosoc Sci, 2008. **40**(1): p. 35-52.
13. Akinyemi, R.O., et al., *Knowledge and perception of stroke amongst hospital workers in an African community.* Eur J Neurol, 2009. **16**(9): p. 998-1003.
14. Langhorne, P., *The main components of stroke unit care: what is the question?* Cerebrovasc Dis, 2007. **23**(5-6): p. 464; author reply 465.
15. Connor, M.D., et al., *The burden of stroke in Sub-Saharan Africa.* Am J Prev Med, 2007. **33**(2): p. 172-3.
16. Connor, M.D., et al., *Burden of stroke in black populations in sub-Saharan Africa.* Lancet Neurol, 2007. **6**(3): p. 269-78.
17. Walker, R., *Stroke mortality in urban and rural Tanzania. Adult Morbidity and Mortality project. .* Lancet, 2000. **355**: p. 1684-1687.
18. Feigin, V.L., et al., *Worldwide stroke incidence and early case fatality reported in 56 population-based studies: a systematic review.* Lancet Neurol, 2009. **8**(4): p. 355-69.
19. WHO., *The World Health Report 2004:changing history. Geneva.* 2004.
20. Banerjee, T.K., M.K. Roy, and K.K. Bhoi, *Is stroke increasing in India--preventive measures that need to be implemented.* J Indian Med Assoc, 2005. **103**(3): p. 162, 164, 166 passim.
21. SASPI, project, and team, *Prevalence of stroke survivors in rural SouthAfrica: results from the Southern Africa stroke prevention initiative (SASPI)Agincourt field site.* Stroke, 2004. **35**: p. 627-32.
22. Reddy, S., *Cardiovascular diseases in non-western countries.* NEJM, 2004. **350**: p. 24.
23. Nakibuuka, J., *A descriptive epidemiological study on stroke in Kampala, Uganda: A hospital based study.* AJNS, 2012. **31**(1).
24. Mayega, R.W., et al., *Modifiable socio-behavioural factors associated with overweight and hypertension among persons aged 35 to 60 years in eastern Uganda.* PLoS One, 2012. **7**(10): p. e47632.

25. Baalwa, J., et al., *Prevalence of overweight and obesity in young adults in Uganda.* Afr Health Sci. **10**(4): p. 367-73.
26. Bimenya, G.S., et al., *Plasma cholesterol and related lipid levels of seemingly healthy public service employees in Kampala, Uganda.* Afr Health Sci, 2006. **6**(3): p. 139-44.
27. Lasky, D., et al., *Obesity and gender differences in the risk of type 2 diabetes mellitus in Uganda.* Nutrition, 2002. **18**(5): p. 417-21.
28. Maher, D., et al., *Epidemiology of hypertension in low-income countries: a cross-sectional population-based survey in rural Uganda.* J Hypertens, 2011. **29**(6): p. 1061-8.
29. Wamala, J.F., et al., *Prevalence factors associated with hypertension in Rukungiri district, Uganda--a community-based study.* Afr Health Sci, 2009. **9**(3): p. 153-60.
30. Mondo, C., *The prevalence and distribution of non-communicable diseases and their risk factors in Kasese district, Uganda.* Uganda Medical Journal, 2012. **1**(001): p. 10-16.
31. Mpabulungi, L. and A.S. Muula, *Tobacco use among high shool students in Kampala, Uganda: questionnaire study.* Croat Med J, 2004. **45**(1): p. 80-3.
32. Muula, A.S. and L. Mpabulungi, *Cigarette smoking prevalence among school-going adolescents in two African capital cities: Kampala Uganda and Lilongwe Malawi.* Afr Health Sci, 2007. **7**(1): p. 45-9.
33. Kayima, J., *Prevalence of hypertension among urban and rural populations in Wakiso district.* 2007.
34. Shah, B. and P. Mathur, *Workshop report on stroke surveillance in India. Division of non-communicable diseases, Indian Council of Medical Research, New Delhi.* 2006.
35. Owolabi, M.O., *Taming the burgeoning stroke epidemic in Africa: stroke quadrangle to the rescue.* West Indian Med J, 2011. **60**(4): p. 412-21.
36. Sacco, R.L., et al., *American Heart Association Prevention Conference. IV. Prevention and Rehabilitation of Stroke. Risk factors.* Stroke, 1997. **28**(7): p. 1507-17.
37. WHO., *The Atlas of Heart Disease and Stroke.* 2009.
38. Sico, J., *Serving International Neurology from Uganda.* Neurology today, 2010. **10**(8): p. 4.
39. UBOS, *Projections of demographic trends in Uganda.* 2007. **1**.
40. *Stroke 101 fact sheet National stroke Association.* 2010.
41. Sturm, J.W., et al., *The Avoid Stroke as Soon as Possible (ASAP) general practice stroke audit.* Med J Aust, 2002. **176**(7): p. 312-6.
42. Barnett, H.J., *Clinical evaluation of strokes and threatened strokes.* Clin Neurosurg, 1976. **23**: p. 540-54.
43. Wiredu, E.K. and P.K. Nyame, *Stroke-related mortality at Korle Bu Teaching Hospital, Accra, Ghana.* East Afr Med J, 2001. **78**(4): p. 180-4.
44. Nyame, P.K., K.B. Jumah, and S. Adjei, *Computerised tomographic scan of the head in evaluation of stroke in Ghanaians.* East Afr Med J, 1998. **75**(11): p. 637-9.
45. O'Donnell, M.J., et al., *Risk factors for ischaemic and intracerebral haemorrhagic stroke in 22 countries (the INTERSTROKE study): a case-control study.* Lancet.
46. Nilsson, O. and A. Lindgren, *Incidence of intracerebral and subarachnoid haemorrhage in Southern Korea.* J.Neurol.Neurosurg.Psychiatry 2000. **69**: p. 601-607.
47. Jonatham, R., *Beyong hypertension: unravelling the causes of intracerebral hemorrhage.* Stroke, 2002. **33**(5): p. 1190.
48. Bath, P.M. and K.R. Lees, *ABC of arterial and venous disease. Acute stroke.* BMJ, 2000. **320**(7239): p. 920-3.
49. Brandt, T., A.J. Grau, and W. Hacke, *Severe stroke.* Baillieres Clin Neurol, 1996. **5**(3): p. 515-41.
50. Yikona, J., *Prevention of hypertension and stroke in Africa.* Lancet, 2000. **356**(9230): p. 678-9.
51. Rosman, K.D., *The epidemiology of stroke in an urban black population.* Stroke, 1986. **17**(4): p. 667-9.

52. Bruno, A., et al., *Acute blood glucose level and outcome from ischemic stroke. Trial of ORG 10172 in Acute Stroke Treatment (TOAST) Investigators.* Neurology, 1999. **52**(2): p. 280-4.
53. Bayley, M., et al., *Balancing evidence and opinion in stroke care: the 2008 best practice recommendations.* CMAJ, 2008. **179**(12): p. 1247-9.
54. Adams, H.P., Jr., et al., *Guidelines for the early management of adults with ischemic stroke: a guideline from the American Heart Association/American Stroke Association Stroke Council, Clinical Cardiology Council, Cardiovascular Radiology and Intervention Council, and the Atherosclerotic Peripheral Vascular Disease and Quality of Care Outcomes in Research Interdisciplinary Working Groups: The American Academy of Neurology affirms the value of this guideline as an educational tool for neurologists.* Circulation, 2007. **115**(20): p. e478-534.
55. Wright, L., et al., *Stroke management: updated recommendations for treatment along the care continuum.* Intern Med J, 2012. **42**(5): p. 562-9.
56. Swain, S., et al., *Diagnosis and initial management of acute stroke and transient ischaemic attack: summary of NICE guidance.* BMJ, 2008. **337**: p. a786.
57. Castillo, J., *[Transient ischemic attack. Ad hoc Editorial Committee of the Task Force on Cerebrovascular Diseases of SEN].* Neurologia, 1998. **13 Suppl 3**: p. 11-2.
58. Greer, D.M., et al., *Impact of fever on outcome in patients with stroke and neurologic injury: a comprehensive meta-analysis.* Stroke, 2008. **39**(11): p. 3029-35.
59. Ginsberg, M.D., *Adventures in the pathophysiology of brain ischemia: penumbra, gene expression, neuroprotection: the 2002 Thomas Willis Lecture.* Stroke, 2003. **34**(1): p. 214-23.
60. Williams, C.A., et al., *A brief descriptive analysis of stroke features in a population of patients from a large urban hospital in Richmond, Virginia, a city within the 'stroke belt'.* Neuroepidemiology, 2003. **22**(1): p. 31-6.
61. Langhorne, P., et al., *Medical complications after stroke: a multicenter study.* Stroke, 2000. **31**(6): p. 1223-9.
62. Den Hertog, H.M., et al., *Cooling therapy for acute stroke.* Cochrane Database Syst Rev, 2009(1): p. CD001247.
63. *Guidelines for management of ischaemic stroke and transient ischaemic attack 2008.* Cerebrovasc Dis, 2008. **25**(5): p. 457-507.
64. *Health care guideline: diagnosis and initial treatment of ischemic stroke. .* 2008.
65. Ezzeddine, M.A., et al., *Blood pressure management in patients with acute stroke: pathophysiology and treatment strategies.* Neurosurg Clin N Am, 2006. **17 Suppl 1**: p. 41-56.
66. *Management of patientswith stroke: identification and management of dysphagia. .* 2004.
67. *Management of patients with stroke or TIA: assessment, investigation, immediate management and secondary prevention. .* 2008.
68. Thorogood, M., et al., *Secondary prevention of stroke--results from the Southern Africa Stroke Prevention Initiative (SASPI) study.* Bull World Health Organ, 2004. **82**(7): p. 503-8.
69. Connor, M.D., G. Modi, and C.P. Warlow, *Differences in the nature of stroke in a multiethnic urban South African population: the Johannesburg hospital stroke register.* Stroke, 2009. **40**(2): p. 355-62.
70. Ingeman, A., et al., *Quality of care and mortality among patients with stroke: a nationwide follow-up study.* Med Care, 2008. **46**(1): p. 63-9.
71. Thijs, V., et al., *Organisation of inhospital acute stroke care and minimum criteria for stroke care units. Recommendations of the Belgian Stroke Council.* Acta Neurol Belg, 2009. **109**(4): p. 247-51.
72. de Villiers, L., S.Z. Kalula, and V.C. Burch, *Does multidisciplinary stroke care improve outcome in a secondary-level hospital in South Africa?* Int J Stroke, 2009. **4**(2): p. 89-93.
73. Jay, W., *Emergency department stroke and transient ischaemic attack care bundle.* 2009.

74. Nor, A.M., et al., *The Recognition of Stroke in the Emergency Room (ROSIER) scale: development and validation of a stroke recognition instrument.* Lancet Neurol, 2005. **4**(11): p. 727-34.
75. Stern, E.B., et al., *Community education for stroke awareness: An efficacy study.* Stroke, 1999. **30**(4): p. 720-3.
76. Reschja, et al., *Comparative autopsy studies on cerebral atherosclerosis in Nigerian and Senegal Negroes, American Negroesand Caucasians.* Atherosclerosis, 1970. **12**(3): p. 401-7.
77. *Cholesterol, diastolic blood pressure, and stroke: 13000 strokes in 450,000 people in 45 prospective cohorts.* Lancet, 1995. **346**: p. 1647-1653.
78. *Blood pressure, cholesterol, and stroke in Eastern Asia. .* Lancet, 1998. **352**: p. 1801-1807.
79. Lewington, S., et al., *Age-specific relevance of usual blood pressure to vascular mortality: a meta-analysis of individual data for one million adults in 61 prospective studies.* Lancet, 2002. **360**(9349): p. 1903-13.
80. Sacco, R.L., D.E. Kargman, and M.C. Zamanillo, *Race-ethnic differences in stroke risk factors among hospitalized patients with cerebral infarction: the Northern Manhattan Stroke Study.* Neurology, 1995. **45**(4): p. 659-63.
81. Fang, J., C. Ayala, and F. Loustalot, *Association of birthplace and self-reported hypertension by racial/ethnic groups among US adults--National Health Interview Survey, 2006-2010.* J Hypertens, 2012. **30**(12): p. 2285-92.
82. Poulter, N., et al., *Blood pressure and associated factors in a rural Kenyan community.* Hypertension, 1984. **6**(6 Pt 1): p. 810-3.
83. Lawes, C.M., et al., *Blood pressure and the global burden of disease 2000. Part II: estimates of attributable burden.* J Hypertens, 2006. **24**(3): p. 423-30.
84. Kannel, W.B., *Risk stratification in hypertension: new insights from the Framingham Study.* Am J Hypertens, 2000. **13**(1 Pt 2): p. 3S-10S.
85. Donahue, R.P., et al., *Alcohol and hemorrhagic stroke. The Honolulu Heart Program.* JAMA, 1986. **255**(17): p. 2311-4.
86. *Three million Ugandans are drunkards.*
87. Barrett and C. L., *Obesity, atherosclerosis and coronary artery disease.* Ann. Intern. Med, 1985. **103**: p. 1010-1019.
88. Mathew, B., *Africa's growing obesity problem.* 2010.
89. Ziraba, A.K., J.C. Fotso, and R. Ochako, *Overweight and obesity in urban Africa: A problem of the rich or the poor?* BMC Public Health, 2009. **9**: p. 465.
90. Steyn, K., et al., *Risk factors associated with myocardial infarction in Africa: the INTERHEART Africa study.* Circulation, 2005. **112**(23): p. 3554-61.
91. Wendel-Vos, G.C., et al., *Physical activity and stroke. A meta-analysis of observational data.* Int J Epidemiol, 2004. **33**(4): p. 787-98.
92. Regina, G. and J. Melanie, *Physical inactivity and sedentary behaviour among school children: A 24- country comparison department of chronic diseases and health promotion, World Health organisation.* 2008.
93. Rastenyte, D., J. Tuomilehto, and C. Sarti, *Genetics of stroke--a review.* J Neurol Sci, 1998. **153**(2): p. 132-45.
94. Wasswa, H., *Uganda struggles to cope with rise in diabetes incidence.* BMJ, 2006. **333**(7570): p. 672.
95. Almdal, T., et al., *The independent effect of type 2 diabetes mellitus on ischemic heart disease, stroke, and death: a population-based study of 13,000 men and women with 20 years of follow-up.* Arch Intern Med, 2004. **164**(13): p. 1422-6.
96. Karapanayiotides, T., et al., *Stroke patterns, etiology, and prognosis in patients with diabetes mellitus.* Neurology, 2004. **62**(9): p. 1558-62.

97. McGruder, H.F., et al., *Racial and ethnic disparities in cardiovascular risk factors among stroke survivors: United States 1999 to 2001.* Stroke, 2004. **35**(7): p. 1557-61.
98. Ditscherlein, G., *Renal histopathology in hypertensive diabetic patients.* Hypertension, 1985. **7**(6 Pt 2): p. II29-32.
99. Bateganya, M., *Prevaleence of hypertension among 100 diabetic patients at Mulago hospital in Uganda.* 2002.
100. Fogelholm, R. and K. Murros, *Cigarette smoking and subarachnoid haemorrhage: a population-based case-control study.* J Neurol Neurosurg Psychiatry, 1987. **50**(1): p. 78-80.
101. Bonita, R., et al., *Passive smoking as well as active smoking increases the risk of acute stroke.* Tob Control, 1999. **8**(2): p. 156-60.
102. Ferro, J.M., *Cardioembolic stroke: an update.* Lancet Neurol, 2003. **2**(3): p. 177-88.
103. Ellis, J., et al., *Echocardiographic, chest X-ray and electrocardiogram findings in children presenting with heart failure to a Ugandan paediatric ward.* Trop Doct, 2007. **37**(3): p. 149-50.
104. Beaton, A., et al., *Echocardiography screening for rheumatic heart disease in Ugandan schoolchildren.* Circulation, 2012. **125**(25): p. 3127-32.
105. Thorvaldsen, P., et al., *Stable stroke occurrence despite incidence reduction in an aging population: stroke trends in the danish monitoring trends and determinants in cardiovascular disease (MONICA) population.* Stroke, 1999. **30**(12): p. 2529-34.
106. Kannel, W.B., et al., *Epidemiologic features of chronic atrial fibrillation: the Framingham study.* N Engl J Med, 1982. **306**(17): p. 1018-22.
107. Flemming, H. and S. Baily, *Mitral valve disease, systematic embolisation and anticoagulants.* Postgrad.med.J, 1975. **47**: p. 599-604.
108. *Predictors of thromboembolism in atrial fibrillation: II. Echocardiographic features of patients at risk. The Stroke Prevention in Atrial Fibrillation Investigators.* Ann Intern Med, 1992. **116**(1): p. 6-12.
109. Weintraub, W.S., et al., *Value of primordial and primary prevention for cardiovascular disease: a policy statement from the American Heart Association.* Circulation, 2011. **124**(8): p. 967-90.
110. Yusuf, S., et al., *Effect of potentially modifiable risk factors associated with myocardial infarction in 52 countries (the INTERHEART study): case-control study.* Lancet, 2004. **364**(9438): p. 937-52.
111. Bowman, T.S., et al., *Cholesterol and the risk of ischemic stroke.* Stroke, 2003. **34**(12): p. 2930-4.
112. Ebrahim, S., et al., *Serum cholesterol, haemorrhagic stroke, ischaemic stroke, and myocardial infarction: Korean national health system prospective cohort study.* BMJ, 2006. **333**(7557): p. 22.
113. Popa-Wagner, A., et al., *Pathophysiology of the vascular wall and its relevance for cerebrovascular disorders in aged rodents.* Curr Neurovasc Res, 2010. **7**(3): p. 251-67.
114. Stern, B. and P. Pullicino, *Risk factors for stroke.* American Academy for neurology Continuum, 2003. **9**(2): p. 53-64.
115. *Risk factors for stroke and types of stroke in persons with isolated systolic hypertension (SHEP) study.* Stroke, 1998. **29**: p. 1333-1340.
116. Smith, D.B., et al., *Gender differences in the Colorado Stroke Registry.* Stroke, 2009. **40**(4): p. 1078-81.
117. Knottnerus, I.L., et al., *Family history of stroke is an independent risk factor for lacunar stroke subtype with asymptomatic lacunar infarcts at younger ages.* Stroke, 2011. **42**(5): p. 1196-200.
118. An, Y.C., et al., *[Risk factors on the recurrence of ischemic stroke and the establishment of a Cox's regression model].* Zhonghua Liu Xing Bing Xue Za Zhi, 2011. **32**(8): p. 816-20.

119. Connor, M.D., et al., *Cerebral infarction in adult AIDS patients: observations from the Edinburgh HIV Autopsy Cohort.* Stroke, 2000. **31**(9): p. 2117-26.
120. Hoffmann, M., et al., *Cerebrovascular disease in young, HIV-infected, black Africans in the KwaZulu Natal province of South Africa.* J Neurovirol, 2000. **6**(3): p. 229-36.
121. Lewis, W., *Cardiomyopathy in AIDS: a pathophysiological perspective.* Prog Cardiovasc Dis, 2000. **43**(2): p. 151-70.
122. Mochan, A., M. Modi, and G. Modi, *Stroke in black South African HIV-positive patients: a prospective analysis.* Stroke, 2003. **34**(1): p. 10-5.
123. *Release of key results of the 2011 Uganda AIDs indicator survey.* 2012.
124. Truelsen, T., et al., *Self-reported stress and risk of stroke: the Copenhagen City Heart Study.* Stroke, 2003. **34**(4): p. 856-62.
125. May, M., et al., *Does psychological distress predict the risk of ischemic stroke and transient ischemic attack? The Caerphilly Study.* Stroke, 2002. **33**(1): p. 7-12.
126. Bergen, D.C. and D. Silberberg, *Nervous system disorders: a global epidemic.* Arch Neurol, 2002. **59**(7): p. 1194-6.
127. Makani, J., T.N. Williams, and K. Marsh, *Sickle cell disease in Africa: burden and research priorities.* Ann Trop Med Parasitol, 2007. **101**(1): p. 3-14.
128. Serjeant, G.R. and C.M. Ndugwa, *Sickle cell disease in Uganda: a time for action.* East Afr Med J, 2003. **80**(7): p. 384-7.
129. Makani, J., *Stroke in sickle cell disease in Africa: case report.* East Afr Med J, 2004. **81**(12): p. 657-9.
130. Nantulya, F.N., *Neurological complications associated with sickle cell anaemia: an experience at the Aga Khan Hospital, Nairobi.* East Afr Med J, 1989. **66**(10): p. 669-77.
131. Baylet, R., et al., *[Survey on the use of tobacco in traditional areas and in urbanized areas in Senegal].* Bull Soc Med Afr Noire Lang Fr, 1974. **19**(1): p. 36-40.
132. Levine, S.R., *Hypercoagulable states and stroke: a selective review.* CNS Spectr, 2005. **10**(7): p. 567-78.
133. Wamala, J., et al., *Prevalence factors associated with Hypertension in Rukungiri District, Uganda - A Community-Based Study.* Afr Health Sci, 2009. **9**(3): p. 153-60.
134. *Primary prevention of ischaemic stroke.* Stroke, 2006. **37**: p. 1583-1633.
135. Kamran, S., et al., *The level of awareness of stroke risk factors and symptoms in the Gulf Cooperation Council countries: Gulf Cooperation Council stroke awareness study.* Neuroepidemiology, 2007. **29**(3-4): p. 235-42.
136. Bham, Z. and E. Ross, *Traditional and western medicine: cultural beliefs and practices of South African Indian Muslims with regard to stroke.* Ethn Dis, 2005. **15**(4): p. 548-54.
137. Hundt, G.L., M. Stuttaford, and B. Ngoma, *The social diagnostics of stroke-like symptoms: healers, doctors and prophets in Agincourt, Limpopo Province, South Africa.* J Biosoc Sci, 2004. **36**(4): p. 433-43.
138. Comoro, C., et al., *Local understanding, perceptions and reported practices of mothers/guardians and health workers on childhood malaria in a Tanzanian district--implications for malaria control.* Acta Trop, 2003. **87**(3): p. 305-13.
139. Kamat, V.R., *"I thought it was only ordinary fever!" cultural knowledge and the micropolitics of therapy seeking for childhood febrile illness in Tanzania.* Soc Sci Med, 2006. **62**(12): p. 2945-59.
140. Green, M., *Public reform and the privatisation of poverty: some institutional determinants of health seeking behaviour in southern Tanzania.* Cult Med Psychiatry, 2000. **24**(4): p. 403-30.
141. Tibandebage, P. and M. Mackintosh, *The market shaping of charges, trust and abuse: health care transactions in Tanzania.* Soc Sci Med, 2005. **61**(7): p. 1385-95.
142. Reeves, M.J., J.G. Hogan, and A.P. Rafferty, *Knowledge of stroke risk factors and warning signs among Michigan adults.* Neurology, 2002. **59**(10): p. 1547-52.

143. Sug Yoon, S., et al., *Knowledge and perception about stroke among an Australian urban population.* BMC Public Health, 2001. **1**: p. 14.
144. Beckerleg, S., *Medical pluralism and islam in swahili communities in Kenya. .* Medical Anthropology, 1994. **8**(3): p. 299-313.
145. Kusimba, J., et al., *Traditional healers and the management of sexually transmitted diseases in Nairobi, Kenya.* Int J STD AIDS, 2003. **14**(3): p. 197-201.
146. Borhani Haghighi, A., et al., *Knowledge and attitude towards stroke risk factors, warning symptoms and treatment in an Iranian population.* Med Princ Pract, 2010. **19**(6): p. 468-72.
147. Hickey, A., et al., *Stroke awareness in the general population: knowledge of stroke risk factors and warning signs in older adults.* BMC Geriatr, 2009. **9**: p. 35.
148. Sug Yoon, S., et al., *Knowledge of stroke risk factors, warning symptoms, and treatment among an Australian urban population.* Stroke, 2001. **32**(8): p. 1926-30.
149. Al Shafaee, M.A., S.S. Ganguly, and A.R. Al Asmi, *Perception of stroke and knowledge of potential risk factors among Omani patients at increased risk for stroke.* BMC Neurol, 2006. **6**: p. 38.
150. Wahab, K.W., et al., *Awareness of warning signs among suburban Nigerians at high risk for stroke is poor: a cross-sectional study.* BMC Neurol, 2008. **8**: p. 18.
151. Weltermann, B.M., et al., *Stroke knowledge among stroke support group members.* Stroke, 2000. **31**(6): p. 1230-3.
152. Parahoo, K., et al., *Stroke: awareness of the signs, symptoms and risk factors--a population-based survey.* Cerebrovasc Dis, 2003. **16**(2): p. 134-40.
153. Pancioli, A.M., et al., *Public perception of stroke warning signs and knowledge of potential risk factors.* JAMA, 1998. **279**(16): p. 1288-92.
154. Bonita, R., K. Strong, and M. de Courten, *From surveys to surveillance.* Rev Panam Salud Publica, 2001. **10**(4): p. 223-5.
155. Owolabi, M.O., S. Ugoya, and T. Platz, *Racial disparity in stroke risk factors: the Berlin-Ibadan experience; a retrospective study.* Acta Neurol Scand, 2009. **119**(2): p. 81-7.
156. Hu, F.B., et al., *Physical activity and risk of stroke in women.* JAMA, 2000. **283**(22): p. 2961-7.
157. Mayfield, D., G. McLeod, and P. Hall, *The CAGE questionnaire: validation of a new alcoholism screening instrument.* Am J Psychiatry, 1974. **131**(10): p. 1121-3.
158. NICE, *NICE clinical guideline 127; diagnosing hypertension.* 2011.
159. *Multicenter trial of hemodilution in ischemic stroke--background and study protocol. Scandinavian Stroke Study Group.* Stroke, 1985. **16**(5): p. 885-90.
160. Adams, H.P., Jr., et al., *Classification of subtype of acute ischemic stroke. Definitions for use in a multicenter clinical trial. TOAST. Trial of Org 10172 in Acute Stroke Treatment.* Stroke, 1993. **24**(1): p. 35-41.
161. *Medical disability guidelines for definition of haemorrhagic stroke on computerised tomography scan.*
162. Bonita, R. and R. Beaglehole, *"Modification of Rankin scale: Recovery of motor function after stroke".* Stroke, 1988. **19**(12): p. 1497-1500.
163. Collin, C., et al., *The Barthel ADL Index: a reliability study.* Int Disabil Stud, 1988. **10**(2): p. 61-3.
164. Teasdale, G. and B. Jennett, *Assessment of coma and impaired consciousness. A practical scale.* Lancet, 1974. **2**(7872): p. 81-4.
165. Somes, J. and D.L. Bergman, *ABCDs of acute stroke intervention.* J Emerg Nurs, 2007. **33**(3): p. 228-34.
166. Sitoh, Y., et al., *Bedside assessment of swallowing: A useful tool for dysphagia in an acute geriatric ward.* Singapore Med J, 2000. **41**(8): p. 376-381.
167. Grise, E.M. and O. Adeoye, *Blood pressure control for acute ischemic and hemorrhagic stroke.* Curr Opin Crit Care, 2012. **18**(2): p. 132-8.

168. Gonzalvo, J. and A. Zillich, *Accuracy of automated community pharmacy-based blood pressure devices.* J Am Pharm Assoc (2003), 2011. **51**(3): p. 408-11.
169. *The European food information council. The basics: Obesity and overweight.* 2006.
170. *Executive Summary of The Third Report of The National Cholesterol Education Program (NCEP) Expert Panel on Detection, Evaluation, And Treatment of High Blood Cholesterol In Adults (Adult Treatment Panel III).* JAMA, 2001. **285**(19): p. 2486-97.

APPENDIX 1: STROKE AND TRANSIENT ISCHEMIC ATTACK (TIA) CARE BUNDLE [73]

All people presenting to emergency departments with stroke like symptoms should receive

1. Rapid initial stroke screen- ROSIER SCALE

2. $ABCD^2$ assessment when TIA is suspected

3. Urgent CT scan of head or MRI (considered as soon as possible but certainly < 24 hours)

4. Nil by mouth until bedside swallow screen (within 24 hours for stroke)

5. Aspirin (150-300mg as loading dose unless contraindicated) as soon as possible if haemorrhage excluded (considered within 48 hours)

6. Physiological monitoring and management:

    - Neurological status- regular monitoring to identify baseline and identify change

    - Blood glucose- Cautious treatment of markedly elevated blood glucose levels; early, intensive maintenance of euglycemia is not recommended. Avoid hypoglycemia

    - Blood pressure- cautious lowering by no more than 10-20% if extremely high ≥220/120mm Hg; monitor for neurological deterioration. Avoid hypotension

    - Body temperature – maintain euthermia

    - Hydration status- maintain euvolemia

APPENDIX 2: AUDIT FORM

STROKE AND TRANSIENT ISCHEMIC ATTACK (TIA) CARE BUNDLE

Project lead...

Audit period ..../.........../.......... to ..../........../...........

Date	Patient ID	Assessment		Urgent CT scan	Nil oral prior to BSS	Immediate aspirin following CT scan	Physiological monitoring					ED LOS	ED discharge diagnosis	Bundle completed
		Stroke screen	$ABCD^2$ for TIA				Neuro	Temp	BP	Blood glucose	Hydration			
		Y/N	Y (or NA) /N	Y/N	Y (or NA) /N	Y/(or CI)/N	Y/N	Y/N	Y/N	Y/N	Y/N	Hrs	Stroke/TIA	Y/N
	#pts	#Y	#Y+NA	#Y	#Y+NA	#Y+CI	#Y	#Y	#Y	#Y	#Y	Median LOS	#stroke #TIA	%complete

APPENDIX 3: ROSIER SCALE

Recognition of stroke in the emergency room (ROSIER) [74]

Patient ID

Assessment Date Time

Symptom onset Date Time

GCS E=...... M=....... V=........ Total.......... BP......./...... B.glucose ....mmol/l

*if B.glucose <3.5mmol/L, treat urgently and reassess once blood glucose normal

Has there been loss of consciousness or syncope?	Y (-1) [ ]	N (0) [ ]
Has there been seizure activity?	Y (-1) [ ]	N (0) [ ]

Is there a NEW ACUTE onset (or awakening from sleep)

I.	Asymmetric facial weakness	Y (+1) [ ]	N (0) [ ]
II.	Asymmetric arm weakness	Y (+1) [ ]	N (0) [ ]
III.	Asymmetric leg weakness	Y (+1) [ ]	N (0) [ ]
IV.	Speech disturbance	Y (+1) [ ]	N (0) [ ]
V.	Visual field defect	Y (+1) [ ]	N (0) [ ]

Total Score -------------- (-2 to +5)

Provisional diagnosis

Stroke [ ] Non stroke [ ] (Specify) -------------------------------

Note: stroke is unlikely but not completely excluded if total scores are ≤ 0

APPENDIX 4: GLASGOW COMA SCALE

The Glasgow coma score is scored between 3 and 15, 3 being the worst and 15 being the best [164]

**Score each response**	**Best eye response (E)**	**Best verbal response (V)**	**Best motor response (M)**
1	No eye opening	No verbal	No motor response
2	Eye opening to pain	Incomprehensible sounds	Extension to pain
3	Eye opening to verbal command	Intelligible but inappropriate words	Flexion to pain
4	Eyes open spontaneously	Confused	Withdrawal from pain
5		Oriented	Localises pain
6			Obeys commands

APPENDIX 5: ABCD2 ASSESSMENT WHEN TIA SUSPECTED [165]

A Age: ≥ 60 years (1 point

B Blood pressure: >140/90 mmHg (1 point)

C Clinical features: Unilateral weakness (2 points), speech impairment without weakness (1 point)

D Duration: More than >60 min (2 points), more than 10-59 min (1 point)

D Diabetes (1 point)

Tool interpretation (REF)

>4 HIGH risk; ≤4 LOW risk Maximum score 7 points

APPENDIX 6: DYSPHAGIA SCREENING TOOL

Bed side assessment of swallowing is commenced when a patient has undergone a CT scan [166].

A patient should be able to meet the pre-feeding requirements:

- Ability to maintain alertness for at least 20 minutes
- Ability to sit upright (placed in high sitting, with hips flexed at 90° to trunk and shoulders positioned over hips)
- Head control

If the patient fails to meet these requirements, they are fed enterally via nasogastric tube

If the patient meets the pre-feeding requirements, they are made to swallow 30mls of water while seated in the upright position

Observations made

1. Delayed swallowing (if the oropharyngeal transit time exceeds 2 seconds)
2. Presence of drooling (inability to retain the drink within the oral cavity together with pooling within the mouth and spillage from the corner of the mouth)
3. Cough during or within 1 minute of swallowing
4. Dysphonia (presence of a post swallow wet or hoarse voice quality)

Interpretation of assessment

Category 1	Normal swallowing function if no abnormalities were found on assessment<br>(Regular diet regimes)
Category 2	Mild swallowing impairment if single abnormality on bedside assessment<br>If delayed swallowing with oropharyngeal transit time of 2-3 seconds<br>(Modified diet of blended consistency with thickened fluids)
Category 3	Severe swallowing impairment if 2 or more abnormalities on bed side Assessment<br>Or significant delay in initiation of swallowing (delay arbitrarily defined as $\geq$3 seconds)<br>(Enteral feeding)

Refer patients in category 2 and 3 to a speech pathologist for a comprehensive assessment

1. Has the patient undergone a head CT scan Yes [ ] No [ ]

Pre-feeding requirements:

2. Ability to maintain alertness for at least 20 minutes Yes [ ] No [ ]

3. Ability to sit upright (placed in high sitting, with hips flexed at 90° to trunk and shoulders positioned over hips) Yes [ ] No [ ]

4. Head control Yes [ ] No [ ]

5. Does the patient pass all the feeding requirements Yes [ ] No [ ]

If no, don't commence, If yes, give the patient 30mls of water to drink and go to 6

6. Observed delayed swallowing (if the oropharyngeal transit time exceeds 2 seconds) Yes [ ] No [ ]

7. Observed presence of drooling (inability to retain the drink within the oral cavity together with pooling within the mouth and spillage from the corner of the mouth) Yes [ ] No [ ]

8. Observed cough during or within 1 min of swallowing Yes [ ] No [ ]

9. Observed dysphonia (presence of a post swallow wet or hoarse voice quality) Yes [ ] No [ ]

Interpretation of assessment

Category 1 Normal swallowing function if no abnormalities were found on assessment
(Regular diet regimes)

Category 2 Mild swallowing impairment if single abnormality on bedside assessment
If delayed swallowing with oropharyngeal transit time of 2-3 seconds
(Modified diet of blended consistency with thickened fluids)

Category 3 Severe swallowing impairment if 2 or more abnormalities on bed side Assessment
Or significant delay in initiation of swallowing (delay arbitrarily defined as $\geq$3 seconds)
(Enteral feeding)

10. Patient's category 1 [ ] 2 [ ] 3 [ ]

11. Referred to speech therapist Yes [ ] No [ ]

APPENDIX 7(a): PHYSIOLOGIC MONITORING

1. NEUROLOGIC STATUS

Neuro status will be monitored using the Glasgow coma score (GCS) (Appendix 3)

- Record the baseline neurologic assessment on admission as a confirmed stroke patient and monitor for the first 72 hours following this diagnosis.
- Monitor and record the neuro status every 4 to 6 hours
- If the neuro status is deteriorating, assess for cause of deterioration

  (Blood glucose levels, seizures, high temperature, infection, progressing stroke with raised intracranial pressure **etc**)
- Manage the cause of the likely cause of deterioration
  - Blood glucose levels
  - Anti-convulsants
  - CXR
  - Bld culture
  - Mid-stream urine sample
  - Repeat CT scan
- Continue to monitor and record the neuro status 4 hourly

NB: The patient's general clinical condition should be taken into consideration

2. FEVER PROTOCOL

Target temperature is < 37.5^{o}C

- Record the baseline temperature on admission as a confirmed stroke patient and monitor for the first 72 hours following this diagnosis.
- Monitor and record temperature every 4 to 6 hours
- If temperature >37.5^{o}C, remove blankets, tepid sponge, cold packs, keep room cool if patient is in air conditioned unit
- Administer oral paracetamol 1 gm and reassess in an hour

- If the patient is nil by mouth, administer paracetamol via nasogastric tube (NGT) or per rectum (PR) then reassess in an hour
- Continue to monitor and record temperature 4 hourly
- If temperature more than 38°C
  - Inform medical team
  - Consider septic work up (as per hospital/unit policy)
    - I CXR
    - II Mid stream urine sample
    - III Blood cultures, tracheal aspirates etc
- Continue to monitor temperature 4 hourly

NB: The patient's general clinical condition should be taken into consideration

3. BLOOD GLUCOSE PROTOCOL

Target blood glucose level (BGL) is < 10mmol/L (6-10mmol/L)

- Record the baseline BGL on admission as a confirmed stroke patient and monitor for the first 72 hours following this diagnosis.
- Promptly treat BGL >10mmol/L in the first 48 hours (as per hospital/unit policy for stroke patients)

a) If initial finger stick BGL <10mmol/L in non diabetic patient

- Obtain fasting blood glucose by finger prick and from venous blood sample
- Obtain BGL 8 hourly (2 hours post prandial)
- Any BGL >10mmol/L within the first 48 hours should be treated (as per hospital/unit treatment algorithm for stroke patients)
  - If <13 mmmol/L, measure blood glucose again after 1 hour (if patient not yet started eating, irregular feeding)
  - If BGL >13mmol/L give 6 IU IV soluble insulin and repeat after 1 hour. Maintain good hydration status with normal saline.
- Continue monitoring BGL 8 hourly until 72 hours since admission.

b) If initial finger stick BGL >10mmol/L in non diabetic patient

- Treat as per hospital/unit treatment algorithm for stroke patients
- Obtain fasting blood glucose by finger prick and from venous blood sample
- Obtain BGL 8 hourly (2 hours post prandial)
- Any BGL >10mmol/L within the first 48 hours should be treated (as per hospital/unit treatment algorithm for stroke patients)
- Continue monitoring BGL 8 hourly until 72 hours since admission.

c) If initial finger stick BGL <10mmol/L in diabetic patient

- May need to withhold the oral diabetic medications especially if the patient's feeding is not yet regular, is not adequate or is not feeding (hospital/unit algorithm for diabetic stroke patients)
- Obtain BGL 4-6 hourly
- If eating, continue with routine diabetic medications
- Obtain BGL 8 hourly (2 hours post prandial)
- Any BGL >10mmol/L within the first 48 hours should be treated (as per hospital/unit treatment algorithm for stroke patients)
    - If BGL < 13mmol/L, give subcutaneous soluble insulin as per sliding scale. Maintain good hydration status with normal saline.

      If BGL >13mmol/L give 6 IU IV soluble insulin and repeat after 1 hour. Maintain good hydration status with normal saline. If blood glucose still high, you may repeat the IV bolus and measure BGL after an hour
- Continue monitoring BGL 8 hourly until 72 hours since admission.

NB: The patient's general clinical condition should be taken into consideration

4. **BLOOD PRESSURE PROTOCOL** [167]

Target MAP for ischemic stroke is 130mmHg, SBP 180mmHg

Target MAP for haemorrhagic stroke is ≤110mmHg, SBP ≤160mmHg

- Record the baseline BP on admission as a confirmed stroke patient and monitor for the first 72 hours following this diagnosis.

- Promptly treat high BP in the first 48 hours (as per hospital/unit policy for stroke patients)
- Lower BP with intra venous (IV) medication (Labetalol 10-20mg) for patients with SBP > 200mmHg (MAP > 150mmHg). Lower by 10-20% in 24 hours. You can repeat labetalol every 30 min while monitoring BP up to a maximum of 4 doses.
- Lower BP to a goal SBP of 160mmHg (MAP 110) for patients with SBP > 180mmHg (MAP >130)
- Maintain cerebral perfusion pressure (CPP) of >60mmHg if there is suspected intracranial pressure (ICP)
- Following IV medication use, reassess initially after 30 min and then hourly for 2 hours
- Observe for any deterioration
- Monitor BP 4-6 hourly.

a) known chronic hypertensive patient

- If presents with BP>180/105mmHg, lower to about 170/100mmHg
- If BP not high enough to require IV antihypertensive medications, restart anti hypertensive medications orally, NGT.
- If high BPs with deterioration
  - Inform the medical team
  - assess for possible cause (worsening brain oedema with raised intracranial pressure)
  - Consider repeat CT scan of the brain
- Monitor BP 4-6 hourly.

b) Patient without known hypertension

- If presents with BP> 160/95mmHg, lower to a goal of 150/90mmHg. May start IV antihypertensive medication (as per hospital/unit policy for stroke patients) with the aim of reducing BP by not more than 10-20% in 24 hours
- Reassess initially after 30 min and then hourly for 2 hours
- Observe for any deterioration
- If high BPs with deterioration
  - Inform the medical team
  - assess for possible cause (worsening brain oedema with raised intracranial pressure)

- Consider repeat CT scan of the brain

- Monitor BP 4-6 hourly.
- Continue monitoring until 72 hours since admission

NB: The patient's general clinical condition should be taken into consideration

5. HYDRATION STATUS

The aim is to maintain euvolemia

If the patient is receiving IV fluids- Isotonic fluid o.9% normal saline is recommended

- 75-125mls per hour or 2-3L in a day is recommended, adjusted for febrile patients
- If the patient is feeding via a nasogastric tube, provide adequate feeds/fluids (as per hospital/unit NGT feeding policy) to the tune of 2-3L in a day, adjusted for febrile patients
- Monitor hydration status 8 hourly
- Continue monitoring until 72 hours since admission

NB: The patient's general clinical condition should be taken into consideration

APPENDIX 7(b): PHYSIOLOGICAL MONITORING 24 HOUR CHART

Patient ID: ....

Date.. Time.............................

Monitoring day (circle as appropriate) If Day 1 fill in the Baseline (BL) values Day 1 Day 2 Day 3

	**TIME**																							
**Neurostatus (GCS)**																								
**Temp ^{o} C**																								
**BP (mmHg)**																								
**Bld glucose (mmol/L)**																								
**Hydration status**																								

Comments

Information collected by... **Date. ....**

APPENDIX 8: QUESTIONNAIRE (HOSPITAL STUDY-PRE-INTERVENTION)

Improving stroke prevention and outcomes in Uganda: Population survey and hospital based study

Date: …/……/…..   Time………………….   Study number: …………..

Tel contact NOK……………………………   Tel contact…………………………….

SECTION A

I. Demographic data.

1. Age: [ ] years   <55years [ ][1]   ≥ 55 years [ ][2]   [ ]

2. Sex: Male [ ][1]   Female [ ][2.]

3. Tribe: ……………………………..

4. District/county: …………………………

5. Parish ………………………..

6. Religion: Catholic [ ][1] Protestant [ ][2]   Moslem [ ][3] Other, [ ][4]   [ ]

7. Marital status: Single [ ][1] Married [ ][2]   Divorced/separated [ ][3]

Widowed [ ][4]   [ ]

8. Highest level of Education: Never attended [ ][1] Primary [ ][2]

Secondary [ ][3]   Tertiary [ ][4]   [ ]

9. Occupation ………………………….

II ROSIER SCALE (As part of audit form tick what was documented to have been done)

Patient ID

Assessment	Date	Time
10. Symptom onset	Date	Time

11. GCS E=...... M=....... V=........ Total..........

12. BP......./......., ……../…….. Average ………./……..

13. Blood glucose ....mmol/l

*if B.glucose <3.5mmol/L, treat urgently and reassess once blood glucose normal

14. Has there been loss of consciousness or syncope? Y (-1) [ ] N (0) [ ]

15. Has there been seizure activity? Y (-1) [ ] N (0) [ ]

16. Is there a NEW ACUTE onset (or awakening from sleep)

17. I. Asymmetric facial weakness Y (+1) [ ] N (0) [ ]

18. II. Asymmetric arm weakness Y (+1) [ ] N (0) [ ]

19. III. Asymmetric leg weakness Y (+1) [ ] N (0) [ ]

20. IV. Speech disturbance Y (+1) [ ] N (0) [ ]

21. V. Visual field defect Y (+1) [ ] N (0) [ ]

22. Total Score -------------- (-2 to +5)

23. Provisional diagnosis Stroke [ ] Non stroke [ ] (Specify) ---------------------- [ ]

III. ABCD2 ASSESSMENT WHEN TIA SUSPECTED (As part of audit form tick what was documented to have been done)

24. Time since symptom onset in hours………

<4.5 [ ]^{1}>4.5 to <24 [ ]^{2}>24[ ] [ ]

25. Is ABCD^{2} applicable? Yes [ ]^{1} NA [ ]^{2} [ ]

If yes, go to 25

26. **A** Age: ≥ 60 years (1 point) Yes [ ]^{1} No [ ]^{2}

27. **B** Blood pressure: >140/90 mmHg (1 point) Yes [ ]^{1} No [ ]^{2}

28. **C** Clinical features: Unilateral weakness (2 points), Yes [ ]^{1} No [ ]^{2}

29. Speech impairment without weakness (1 point) Yes [ ]^{1} No [ ]^{2}

30. **D** Duration: More than >60 min (2 points), Yes [ ]^{1} No [ ]^{2}

31. More than 10-59 min (1 point) Yes [ ]^{1} No [ ]^{2}

32. **D** Diabetes (1 point) Yes [ ]^{1} No [ ]^{2}

33. Total score -------, >4 HIGH risk [ ]^{1} ≤4 LOW risk [ ]^{2} [ ]

IV HEAD CT SCAN (As part of audit form tick what was documented to have been done)

34. Time when head CT scan done since presenting to Emergency unit …………hrs

<24 hours [ ] > 24 hours [ ] [ ]

35. CT scan findings Stroke [ ] Other pathology [ ] [ ]

If other pathology, stop at 35

If stroke, go to 36

36. If other pathology, specify ………………………………………

(Information so far obtained to be handed to attending team)

37. If CT scan shows stroke but ROSIER score ≤ 0 Yes [ ] No [ ] [ ]

38. Stroke subtype Ischemic [ ] Haemorrhagic [ ] [ ]

V BEDSIDE SWALLOW SCREEN (As part of audit form tick what was documented to have been done)

Pre-feeding requirements:

39. Ability to maintain alertness for at least 20 minutes Yes [ ] No [ ] [ ]

40. Ability to sit upright (placed in high sitting, with hips flexed at 90° to trunk and shoulders positioned over hips) Yes [ ] No [ ] [ ]

41. Head control Yes [ ] No [ ] [ ]

42. Does the patient pass all the feeding requirements Yes [ ] No [ ] [ ]

If no, don't commence with screen

If yes, give the patient 30mls of water to drink (per procedure) and go to 43

43. Observed delayed swallowing (if the oropharyngeal transit time exceeds 2 seconds)
Yes [ ] No [ ] [ ]

44. Observed presence of drooling (inability to retain the drink within the oral cavity together with pooling within the mouth and spillage from the corner of the mouth) Yes [ ] No [ ] [ ]

45. Observed cough during or within 1 min of swallowing Yes [ ]No [ ] [ ]

46. Observed dysphonia (presence of a post swallow wet or hoarse voice quality)
Yes [ ] No [ ] [ ]

Interpretation of assessment

Category 1 Normal swallowing function if no abnormalities were found on assessment (Regular diet regimes)

Category 2 Mild swallowing impairment if single abnormality on bedside assessment
If delayed swallowing with oropharyngeal transit time of 2-3 seconds
(Modified diet of blended consistency with thickened fluids)

Category 3 Severe swallowing impairment if 2 or more abnormalities on bed side Assessment
Or significant delay in initiation of swallowing (delay arbitrarily defined as ≥3 seconds)
(Enteral feeding)

47. Patient's category 1 [ ] 2 [ ] 3 [ ] [ ]

48. Referred to speech therapist Yes [ ] No [ ] [ ]

49. Aspirin 150-300mg given as loading dose within 48 hours (unless contraindicated) if haemorrhagic stroke excluded Yes [ ] No [ ] [ ]

VI PAST MEDICAL HISTORY

Risk factor assessment

(Medical)

50. Did a physician ever tell you that you had a stroke?

Yes [ ][1] No [ ]2 Don't known [ ][3] [ ]

If no to (52),

51. Have you ever suffered from any of the following in a manner that was sudden?

Painless weakness on one side of your body, numbness or a dead feeling on one side of your body, painless loss of vision in one or both eyes, inability to understand what people are saying or inability to express yourself verbally or in writing?

Yes [ ][1] No [ ][2] Don't known [ ][3] [ ]

If no or don't know go to 21

If yes, how long did the symptoms last? < 24 hrs [ ][1]> 24 hrs [ ][2] [ ]

Ever diagnosed or told that you had any of the following?

52. Hypertension: Yes [ ][1] No [ ][2] [ ]

53. Diabetes: Yes [ ][1] No [ ][2] [ ]

54. Have you ever had a blood test for your lipid levels? Yes [ ][1] No [ ][2]

Don't know [ ][3] [ ]

If yes, were they high [ ][1] Normal [ ][2] [ ]

If no go to 55

55. Have you ever had a heart attack: Yes [ ][1] No [ ][2] [ ]

56. Heart disease: Yes [ ][1] No [ ][2] [ ]

57. Heart surgery: Yes [ ][1] No [ ][2] [ ]

Lifestyle/social activities

58. Current smoking: Yes [ ][1] No [ ][2] [ ]

If yes how many cigarettes/day? 5-15 [ ][1] 16-20 [ ][2] ≥21 [ ][3] [ ]

If no go to 59

59. Former smoker: Yes [ ][1] No [ ][2] [ ]

60. Current alcohol consumption: Yes [ ][1] No [ ][2] [ ]

If yes go to 61

If no go to 66

61. (C) Have you ever felt you ought to cut down on your drinking?

Yes [ ][1] No [ ][2] [ ]

62. (A) Have people ever annoyed you by criticizing your drinking?

Yes [ ][1] No [ ][2] [ ]

63. (G) Have you felt guilty or bad about your drinking?

Yes [ ][1] No [ ][2] [ ]

64. (E) Have you ever had a drink first thing in the morning to steady your nerves?

Yes [ ][1] No [ ][2] [ ]

65. Interpretation: No suspected alcohol problem (all no) [ ][1]

Suspected alcohol problem (one yes) [ ][2] [ ]
Alcohol abuse/ dependency (more than one yes) [ ][3]

66. Are you engaged in any sport in the last 3 months? Yes [ ][1] No [ ][2] [ ]

If yes go to 67

If no go to 69

67. About how many times a week do you engage in the sport?

Rarely [ ][1] 1-2 times per week [ ][2] $\geq$ 3 times per week [ ][3] [ ]

68. Does this sport cause perspiration and breathlessness? Yes [ ][1] No [ ][2] [ ]

69. Any current engagement in active physical exercise that caused perspiration and breathlessness? Yes [ ][1] No [ ][2] [ ]

If yes, specify activity-----------------------------

If no go to 71

70. About how many times a week do you engage in this activity? ------------.

Rarely [ ]1 1-2 times per week [ ]2 $\geq$ 3 times per week [ ]3 [ ]

71. Means of transport? Walk [ ]1 Bicycle [ ] 2 Taxi [ ]3 Drive [ ]4
Motor cycle: Ride [ ]5 Ride on [ ]6

Family history

Do any of your relatives suffer from or have suffered from the following conditions?

72. Stroke Yes[ ]1 No[ ]2 Don't know [ ]3 [ ]

If yes, relationship: Paternal [ ]1 Maternal [ ]2 Sibling [ ]3 [ ]

If no go to 44

73. TIA Yes [ ]1 No [ ]2 Don't know[ ]3 [ ]

74. High blood pressure Yes [ ]1 No [ ]2 Don't know [ ]3 [ ]

75. Diabetes Mellitus Yes [ ]1 No [ ]2 Don't know [ ]3 [ ]

76. Heart attack Yes [ ]1 No [ ]2 Don't know [ ]3 [ ]

77. Heart disease Yes [ ]1 No [ ]2 Don't know [ ]3 [ ]

78. Heart surgery Yes [ ]1 No [ ]2 Don't know [ ]3 [ ]

79. High blood cholesterol. Yes[ ]1 No [ ]2 Don't know [ ]3 [ ]

Current medications

80. Anti-hypertensive drugs Yes [ ]1 No [ ]2 [ ]

If yes medication Regular [ ]1 Irregular [ ]2 [ ]

81. Antiplatelet drugs Yes [ ]1 No [ ]2 [ ]

82. Anticoagulants Yes [ ]1 No [ ]2 [ ]

83. Oral/ injectable contraceptives Yes [ ]1 No [ ]2 [ ]

84. Lipid lowering drugs Yes [ ]1 No [ ]2 [ ]

85. Anti-diabetic drugs Yes [ ]1 No [ ]2 [ ]

VII PHYSICAL EXAMINATION

Baseline physiologic parameters Time ---------------------

86. Temperature [ ]^0 C Febrile [ ][1] Afebrile [ ] [2] [ ]

87. Blood pressure ------/----- Systolic/ Diastolic, ----/---- Systolic/ Diastolic,

Average ----/---- Systolic/ Diastolic, >140/90mmHg Yes [ ][1] No [ ][2] [ ]

88. Level of consciousness (GCS) …….. Normal [ ] [1] Impaired [ ][2] [ ]

89. Hydration status Good [ ] Dehydrated [ ] [ ]

90. Finger stick blood glucose --------- <10mmol/L [ ] >10mmol/L [ ] [ ]

Cardiovascular examination

91. Pulse rate: [ ] bpm. Nature Regular [ ] [1] Irregularly Irreg [ ][2] [ ]

92. Carotid Bruits: Yes [ ][1] No [ ][2] [ ]

93. Evidence of valvular pathology Yes [ ][1] No [ ][2] [ ]

94. Apex displaced Yes [ ][1] No [ ][2] [ ]

Neurological exam:

95. Neuro deficit present Yes [ ][1] No [ ][2] [ ]

96. If yes, specify Motor [ ][1] Sensory [ ][2] Global [ ][3] [ ]

97. Dysphasia Yes [ ][1] No [ ][2] [ ]

If yes, specify Motor [ ][1] Sensory [ ][2] Global [ ][3] [ ]

98. Hemi- inattention Yes [ ][1] No [ ][2] Difficult to assess [ ][3] [ ]

99. Evidence of visual field defect Yes [ ][1] No [ ][2] Difficult to assess [ ][3] [ ]

Peripheral Nervous system:

Motor examination

100. Muscle bulk Normal [ ][1] Reduced [ ][2] [ ]

If reduced specify limb: RT upper [ ][1] RT lower [ ][2]

LT upper [ ][3] LT lower [ ][4] [ ]

101. Power 0/5 1/5 2/5 3/5 4/5 5/5

Difficult to assess Yes [ ][1] No [ ][2] [ ]

If difficult to assess go to 102, if not

Specify site/limb. RT upper [ ][1] RT lower [ ][2] [ ]

LT upper [ ][3] LT lower [ ][4] [ ]

102. Reflexes: Normal [ ][1] Hyporeflexia [ ][2] Hyper-reflexia [ ][3] Clonus [ ][4][ ]

Specify site/limb. RT upper [ ][1] RT lower [ ][2] [ ]

LT upper [ ][3] LT lower [ ][4] [ ]

103. Tone: Normal [ ][1] Hypotonia [ ][2] Hypertonia [ ][3] [ ]

Specify site/limb. RT upper [ ][1] RT lower [ ][2]

LT upper [ ][3] LT lower [ ][4] [ ]

Sensory examination

104. Sensory impairment: Yes [ ][1] No [ ][2] Difficult to assess [ ][3] [ ]

If no or difficult to assess go to 105

If yes, specify mode of impairment.

Light touch Yes [ ][1] No [ ][2] [ ]

Pain Yes [ ][1] No [ ][2]

105. Scandinavian stroke scale score …….. Mild [ ] Severe [ ] **[ ]**

VIII. LABORATORY RESULTS.

Complete blood count and ESR

106. Haemoglobin. ------- g/dl. Normal [ ][1] High [ ][2] Low [ ][3] [ ]

107. Platelet counts---------------- Normal [ ][1] High [ ][2] Low [ ][3] [ ]

108. Total WBC count: -------- Normal [ ][1] High [ ] [2] Low [ ][3] [ ]

109. ESR Normal [ ][1] High [ ][2] Low [ ][3] [ ]

110. RPR . Reactive [ ][1] Non-reactive [ ][2] [ ]

111. HIV serology Reactive [ ][1] Non-reactive [ ][2] [ ]

Lipid profile (mg/dl).

112. Total cholesterol……….. < 200 [ ]1 200-239 [ ]2 > 240 [ ]3 [ ]

113. LDL cholesterol……….. < 100 [ ]1 100-129 [ ]2 130-159 [ ]3 [ ]

160-189 [ ] 4 ≥190 [ ]5

114. HDL cholesterol………< 40 [ ]1≥ 60 [ ]2 [ ]

115. Triglycerides……. <150 [ ]1 150-199 [ ]2 200-499 [ ]3≥ 500 [ ]4 [ ]

Blood sugar

116. Fasting finger stick sugar: ---------- mg/dl < 126 [ ]1≥126 [ ]2 [ ]

117. Venous fasting blood sugar …………. mg/dl <126 [ ]1>126[ ]2 [ ]

IX. IMAGING INVESTIGATIONS

Echocardiography/ Doppler ultrasound of carotid arteries/ 12 Lead ECG

118. Evidence of cardioembolic source if any of these is present. Akinetic wall motion, cardiac thrombi, vegetations, VEF of ≤35%, multiple valvular involvement, severe valvular lesions (specify),

If yes state which one--------------- Yes [ ]1 No [ ]2 [ ]

119. Evidence of carotid artery stenosis, Atherosclerosis, Atheromatous plagues,

Yes [ ]1 No [ ]2 [ ]

If yes specify--------------------------

120. Atrial fibrillation on ECG Yes [ ] No [ ] [ ]

CT scan of brain, if hemorrhagic, go to 124

121. CXR finding: Pneumonia Yes [ ] No [ ] [ ]

122. CT scan if ischemic stroke Single lesion [ ] Multiple [ ] [ ]

123. CT scan if ischemic stroke, infarct size …………… (mm)

124. Subtype of ischemic stroke

Lacunar [ ]1 Embolic [ ]2 Atherosclerotic [ ]3 Unspecified [ ] [ ]

125. CT scan if haemorrhagic stroke

Subarachnoid haemorrhage [ ][1] Intracerebral haemorrhage [ ][2]

Both [ ][3] [ ]

X THIRTY DAY FOLLOW UP

126. Length of hospital stay ………..

127. Died during hospitalization Yes [ ] No [ ] [ ]

If yes go to 132

128. Date of discharge …………………………

129. Review date ………………………….

130. Date of notification a week to review date …………………….

131. Notification successfully done yes [ ] No [ ] [ ]

132. Thirty day outcome Alive [ ][1] Dead [ ][2] [ ]

If alive go to 134

133. When died Date …………………… N^{th} day ………

134**.** Barthel index score …… Independent if $\geq$95 [ ] Dependent <95 [ ] [ ]

135. Modified Rankin Score ……. Mild disability (0-2)[ ] Moderate (3-4) [ ]

Severe (5) [ ] [ ]

APPENDIX 9 (a): CONSENT FORM (HOSPITAL STUDY PRE-INTERVENTION)

Study title: Improving stroke prevention and outcomes in Uganda: Population based survey in urban and rural Wakiso and hospital study at Mulago hospital

Principal Investigator: Nakibuuka Jane, Department of Medicine, School of Medicine, Makerere University College of Health Sciences Tel: 0772618111

To be read by/read out to potential participants ***aged 18 years or older***

Before signing this form, read and consider the following information given to you regarding the reasons for the study, procedures, benefits and any other important information. Please ask the interviewer if some of the information is not clear.

1. Why is this study being done?

Stroke is becoming common and a major cause of poor outcomes such as death and disability in low income countries such as Uganda. The exact care given to patients suspected of having stroke and the factors associated with poor outcomes are not well studied in our setting, yet this is very crucial for planning health services. We plan to conduct a study to determine the outcomes after 30 days of stroke onset (living or not, independent or not, able to do activities of daily living such as dressing, combing hair or not) and the factors associated with poor outcomes in order to inform relevant stake holders on planning better health services to treat and prevent stroke.

2. Why have I been chosen to take part in the study?

You have been selected because you have a stroke and you have presented to Mulago hospital during a time when this study is taking place. We will be asking 127 other men and women to participate in this study. If this is your first time to participate in such a study, use this opportunity to familiarise yourself with the study and ask any questions that you may have.

3. What will happen to me if I decide to take part in the study?

If you choose to take part in the study, you will be expected to answer questions about your current and past medical illness in relation to stroke and its risk factors and you will be examined too. We will request you to stop feeding for at least 8 hours overnight so that we can obtain a 6ml blood sample from a vein on your arm the following morning. This will be done is a proper way using a sterile needle and syringe. The blood that we collect will be placed in a sample bottle and sent to our laboratory for testing. We will test for blood fat levels, complete blood count and syphilis. If your HIV status is not known, results of the blood test will be obtained from the nursing sister in-charge of the HIV routine counselling and testing programme on your ward. Imaging studies like echocardiography and Doppler ultrasound of the carotid arteries may also be done if examination of your heart is found to be abnormal. A chest xray will be done if you have a fever. All the results of the tests will be

returned and placed in your file. They will be used in deciding your care by the doctors caring for you on the ward. These results will be given to you too if you wish to know them.

4. Can I refuse to take part in the study or stop being in the study?

It is up to you to decide on participating in the study or not. You can also stop being in the study at any time you want without giving a reason.

5. What risks can I expect from being in the study?

If you take part in the study, the risks to you are very slight. The procedure of drawing blood from your veins may cause some pain and discomfort. You may bleed a little from the site, however the amount of blood drawn will be too small to pose any health hazard. By participating in this study, you may lose some privacy, but as the investigator, I assure you that all your medical records will be kept confidential. We will use a code number to identify you and all records will be kept under lock and key. Your identity will not be used in any reports or publications resulting from the study.

6. Are there any benefits to me from participating in the study?

You will be interviewed, examined and investigated for major risk factors for stroke as well as factors known to cause poor outcomes after stroke. This will be done at no cost to you. Results of tests will be placed in your ward file to help the doctors better manage your condition. From information obtained from this study, it may be possible to contribute towards improving stroke prevention and outcomes such as death.

7. For how long will I be followed up if I decide to participate in the study?

You will be followed up for a period of 30 days. We would however also like to follow you up for a period of up to 1 year to know how you are doing. If you agree to this additional follow up period, you will be required to sign another statement of consent at the end of this form.

8. Whom can I ask if I have any questions about the study?

We would like to answer any questions at any time about this research study. If you have any now, please ask. However, if you have any questions in future you can contact me at the Department of medicine, Mulago hospital. Tel: 0772618111

For any concerns about your rights in this study, please contact the chairman of Makerere School of Medicine Research and Ethics Committee on Tel: (+256) 0414-533541

9. Statement of consent

The nature and purpose of this study has been explained to me and I understand it. I therefore agree to participate in the study.

Name of patient…………………….. ……Signature……………………Date………...

Name of investigator……………………...Signature…………………....Date………....

Name of witness……………………….......Signature……………………Date………....

10. Statement of consent for a one year follow up period

The nature and purpose of this study has been explained to me and I understand it. I therefore agree to be followed up for a period of one year.

Name of patient…………………….. ……Signature……………………Date………...

Name of investigator……………………...Signature…………………....Date……….....

Name of witness……………………….......Signature……………………Date……….....

APPENDIX 9 (b): CONSENT FORM FOR IN-HOSPITAL STUDY (PRE-INTERVENTION) IN LUGANDA

Study title: Improving stroke prevention and outcomes in Uganda: Population based survey in urban and rural Wakiso and hospital study at Mulago hospital

Akulira okunoonyereza: Nakibuuka Jane, asangibwa mu Department of Medicine, School of Medicine, Makerere University College of Health Sciences ku ssimu: 0772618111

*To be read by/read out to potential participants **aged 18 years or older***

Nga tonaba kuteeka mukono ku kiwandiiko kino, ssoma era olowooze ku bwino ono akuweebwa akwata ku nsonga lwaki omusomo gukolebwa, emitendera gyagwo, ebiguganyurwamu ne bwino omulala omukulu. Tukusaba obuuze oyo muntu abuuza ebibuuzo bwekiba ng'omo ku bwino akuweebwa tategerekeka bulungi.

1. **Lwaki omusomo guno gukolebwa?**

Obulwadde bwa situlooko butandise okulabibwa ennyo era nga bukulu mu kuleetawo ebiva mu kulwala ebibi ng'okufa n'okulemala mu nsi zimufuna mpola nga Uganda. Okulabirirwa n'obujjanjabi obuweebwa abalwadde abateeberezebwa okuba n'obulwadde bwa situlooko era n'esonga ezivaako ebiva mu kulwala ebibi tebisomedwako bulungi mu mbeera z'ebitundu byaffe songa kikulu nnyo mu kutegeka empereza ez'ebyobulamu. Tutegeka okulaba okulabirirwa n'obujjanjabi obuweebwa abalwadde abalina obulwadde bwa situlooko n'ensonga ezekuusa ku biva mu kulwala ebibi okusobola okutegeeza bekikwatako ku kutegeka empereza ez'ebyobulamu ezisingako obulungi okujjanjaba era n'okuziyizaamu obulwadde bwa situlooko.

2. **Lwaki nondeddwa okwetaba mu musomo guno?**

Olondedwa kubanga olina obulwadde bwa situlooko era ng'oze mu dwaliro e mulago mu kiseera omusomo mwegukolebwa. Tujja kuba nga tusaba abaami n'abakyala kikumi mu nkaaga mu mwenda (169) okwetaba mu musomo guno. Bweguba nga gwe mulundigwo ogusooka okwetaba mu musomo nga guno, kozesa omukisa guno okutegeera ebikwata ku musomo era obuuze ebibuuzo byonna byoyinza okuba nabyo.

3. **Kiki ekinambaako singa nsalawo okwetaba mu musomo?**

Bwosalawo okwetaba mu musomo, ojja kuba osuubirwa okuddamu ebibuuzo ebikwata ku ndwadde zo zolina kati n'ezemabega ezekuusa ku bulwadde bwa situlooko n'ebyo ebiteeka omuntu mu kabegye k'okumufuna era okeberebwe. Tujja kukusaba oleme kulya okumala essaawa munaana okuyita mukiro tusobole okufuna ogumu ku musaayi/sampo y'omusaayi eweza miiru mukaaga okuva ku musuwa gw'okumukono enkeera kumakya. Kino kijja kukolebwa mu ngeri entuufu nga tukozesa empiso n'ebomba empya okutali buwuka. Omusaayi gwetukungaanya gujja kuteekebwa mu kaccupa omuteekwa sampo era gusindikibwe mu laabu awakeberebwa omusaayi gukeberebwe. Tujja kukebera okulaba

obungi bwa masavu g'omumusaayi, okulaba obungi bwe birungo ebisangibwa mu musaayi mubujuvu bwabyo era ne kabotoogo. Bwoba nga bwoyimiride ku byakawuka ka siriimu tebimanyidwa, ebinaava mu kukebera omusaayi bijja kufunibwa okuva ku akulira ba nansi ku waadiyo abali ku pulogulaamu y'okubudabuda n'okukebera akawuka ka ka siriimu okukolebwa ku buli ajja kuwaadi kw'ebifananyi by'enkola y'omutima eyitibwa echocardiography n'okukubibwa kwekifananyi ekiyitibwa Doppler ultrasound eky'obusuwa obuyitibwa carotid arteries biyinza okukolebwa singa ng'omutimagwo gusangibwa okuba nga ssimulamu bulungi. Byonna ebinaava mu kukeberebwa bijja kuddizibwa era biteekwe mu fayiro yo. Era bijja kukozesebwa mu kusalawo obujjanjabi bwo abasawo abakulabirira ku waadi. Ebinaava mukukebera bijja kukuweebwa bwoba ng'oyagala okubimanya.

4. **Nyinza okugaana okwetaba mu musomo oba nendekeraawo okwetaba mu musomo?**
   Kiri gyoli okusalawo okwetaba mu musomo oba nedda. Era osobola okulekeraawo okubeera mu musomo essaawa yonna woyagalira nga towadde nsonga yonna**.**

5. **Buzibu ki bweba nsuubira obuva mukwetaba mu musomo?**
   Bwewetaba mu musomo guno obuzibu bwoyolekede butono nyo. Omutendera ogw'okujjibwako omusaayi okuva ku misuwa gyo kiyinza okukuleetera obulumi obusaamusaamu n'okutawanyizibwa. Oyinza okufulumya omusaayi omutono okuva mukifo ekyo, naye ng'omusaayi ogunafunibwa gujja kuba mutononyo okubaako akabegye gweguteeka ku bulamubwo. Bwewetaba mu musomo guno, oyinza okufiirwa ebimu ku byamabyo, naye nze nga anoonyereza, nkukakasa nti likodizo/ebiwandiikobyo eby'ekisawo bijja kukuumibwa nga byakyama. Tujja kukozesa ennamba enekusifu/koodi okusobola okukumanya era nga likodizo/ebiwandiikobyo bijja kuterekebwa awantu awasibwa n'ekisumuluzo. Ebikumanyisa tebija kukozesebwa mu lipoota zonna oba ebitabo ebinakubibwa nga biva mu musomo

6. **Waliwo byeganyurwa mu kwetaba mu musomo?**

   Ojja kubuuzibwa ebibuuzo, wekebejjebwe era obe n'okeberebwa ku bintu ebisinga okuteeka abantu mu kabengye k'okufuna obulwadde bwa situlooko n'ensonga/ebintu ebimanyidwa ebileetawo okuba n'ebiva mu kulwala ebibi ng'omaze okufuna situlooko awatali kufulumya ssente zonna. Ebiva mu kukeberebwa bijja kuteekebwa mu fayiroyo ey'okuwaadi kiyambe abasawo okukukwata embeerayo mu ngeri esingako okuba ennungi. Okuva ku bwino afunidwa okuva mu musomo guno, kisoboka okubaako kyekiyamba mu kwongera okulongoosa mu ngeri obulwadde bwa situlooko gyebuziyizibwamu nebimuvaamung'okufa.

7. **Okwetaba mu musomo guno kinatwala banga ki?**
   Kijja kutwala ebanga lya naku asatu. Twandiyagadde okumanya bwoli natte okutusa ebanga lya mwaka gumu kasokedde nga ofuna obulwadde buno obwa situlooko. Singa okiriza okusaba kwaffe kuno, ojja kusabibwa okuteka omukono gwo ku sitaatimenti endala eya obweyamo buno kunkomelero ya form eno.

8. Aani gwenyiza okubuuza singa nina ebibuuzo ebikwata ku musomo?

Abali ku ttimu y'omusomo baagala okukuddamu ebibuuzo byonna byoyinza okuba nabyo essaawa yonna ebikwata ku musomo guno ogw'okunoonyereza. Bwoba n'ekyolina kyonna essaawa eno, tukusaba obuuze. Naye bwoba n'ebibuuzo gyebujja mumaaso oyinza okuntuukirira mu dipatimenti/kitongole kyabasawo eky'edwaliro ly'emulago (Department of medicine, Mulago hospital) ku ssimu: 0772618111 oba akulira okunoonyereza ku ndwadde z'omutima Dr. Mondo Charles, ku Uganda Heart Institute, Mulago Hospital awajjanjabirwa omutima ku ssimu: +256-774460496

Ku bikwata ku kyewebuuza ku ddembelyo mu musomo guno, tukusaba otuukirire ssentebe wa kakiiko ak'ettendekero ly'ekisawo eMakerere akavunanyizibwa okulaba nti eddembe ly'abanoonyerezebwako terityoboolwa mu misomo egikolebwa akayitibwa Makerere School of Medicine Research and Ethics Committee ku ssimu: (+256) 0414-533541

9. Sitaatimenti ey'obweyamwo

Ekika n'ekigendererwa ky'omusomo guno binyinyonyodwa gyendi era bitegede bulungi. Era kati nzikiriza okwetaba mu musomo.

Erinya ly'omulwadde…………………Omukono……….......Ennaku z'omwezi …........

Erinya ly'anoonyereza………………..Omukono……………....Ennaku z'omwezi …….

Erinya ly'omujjulizi…………………..Omukono………………Ennaku z'omwezi…......

10. Sitaatimenti ey'obweyamwo okwetaba mu musomo guno okumala omwaka gumu

Ekika n'ekigendererwa ky'omusomo guno binyinyonyodwa gyendi era bitegede bulungi. Era kati nzikiriza okwetaba mu musomo okumala omwaka gumu.

Erinya ly'omulwadde…………………Omukono……….......Ennaku z'omwezi …........

Erinya ly'anoonyereza………………..Omukono……………....Ennaku z'omwezi …….

Erinya ly'omujjulizi…………………..Omukono……………….Ennaku z'omwezi…......

APPENDIX 10: QUESTIONNAIRE (HOSPITAL STUDY –INTERVENTION)
Improving stroke prevention and outcomes in Uganda: Population survey and hospital based study

Date: …/……/….. Time…………………. Study number: …………..

Tel contact NOK…………………………… Tel contact…………………………….

SECTION A

I. DEMOGRAPHIC DATA.

1. Age: [ ] years <55years [ ][1] ≥ 55 years [ ][2] [ ]

2. Sex: Male [ ][1] Female [ ][2.]

3. Tribe: ……………………………..

4. District/county: …………………………

5. Parish ………………………..

6. Religion: Catholic [ ][1] Protestant [ ][2] Moslem [ ][3] Other, [ ][4] [ ]

7. Marital status: Single [ ][1] Married [ ][2] Divorced/separated [ ][3]

Widowed [ ][4] [ ]

8. Highest level of Education: Never attended [ ][1] Primary [ ][2]

Secondary [ ][3] Tertiary [ ][4] [ ]

11. Occupation ………………………….

II ROSIER SCALE

Patient ID

Assessment Date Time

10. Symptom onset Date Time

11. GCS E=...... M=....... V=........ Total..........

12. BP......./......., ………/…….. Average ………./……..

13. Blood glucose ....mmol/l

*if B.glucose <3.5mmol/L, treat urgently and reassess once blood glucose normal

14. Has there been loss of consciousness or syncope? Y (-1) [ ] N (0) [ ]

15. Has there been seizure activity? Y (-1) [ ] N (0) [ ]

16. Is there a NEW ACUTE onset (or awakening from sleep)

17. I. Asymmetric facial weakness Y (+1) [ ] N (0) [ ]

18. II. Asymmetric arm weakness Y (+1) [ ] N (0) [ ]

19. III. Asymmetric leg weakness Y (+1) [ ] N (0) [ ]

20. IV. Speech disturbance Y (+1) [ ] N (0) [ ]

21. V. Visual field defect Y (+1) [ ] N (0) [ ]

22. Total Score -------------- (-2 to +5)

23. Provisional diagnosis Stroke [ ] Non stroke [ ] (Specify) ---------------------- [ ]

III. ABCD2 ASSESSMENT WHEN TIA SUSPECTED

24. Time since symptom onset in hours………

<4.5 [ ][1]>4.5 to <24 [ ][2]>24[ ] [ ]

25. Is ABCD^2 applicable? Yes [ ][1] NA [ ][2] [ ]

If yes, go to 25

26. **A** Age: ≥ 60 years (1 point) Yes [ ][1] No [ ][2]

27. **B** Blood pressure: >140/90 mmHg (1 point) Yes [ ][1] No [ ][2]

28. **C** Clinical features: Unilateral weakness (2 points), Yes [ ][1] No [ ][2]

29. Speech impairment without weakness (1 point) Yes [ ][1] No [ ][2]

30. **D** Duration: More than >60 min (2 points), Yes [ ] [1] No [ ] [2]

31. More than 10-59 min (1 point) Yes [ ] [1] No [ ] [2]

32. **D** Diabetes (1 point) Yes [ ] [1] No [ ] [2]

33. Total score -------, >4 HIGH risk [ ] [1] ≤4 LOW risk [ ] [2] [ ]

IV HEAD CT SCAN

34. Time when head CT scan done since presenting to Emergency unit ………..hrs

<24 hours [ ] > 24 hours [ ] [ ]

35. CT scan findings Stroke [ ] Other pathology [ ] [ ]

If other pathology, stop at 35

If stroke, go to 36

36. If other pathology, specify ……………………………………

(Information so far obtained to be handed to attending team)

37. If CT scan shows stroke but ROSIER score ≤ 0 Yes [ ] No [ ] [ ]
38. Stroke subtype Ischemic [ ] Haemorrhagic [ ] [ ]

V BEDSIDE SWALLOW SCREEN

Pre-feeding requirements:

39. Ability to maintain alertness for at least 20 minutes Yes [ ] No [ ] [ ]

40. Ability to sit upright (placed in high sitting, with hips flexed at 90° to trunk and shoulders positioned over hips) Yes [ ] No [ ] [ ]
50. Head control Yes [ ] No [ ] [ ]

51. Does the patient pass all the feeding requirements Yes [ ] No [ ] [ ]
If no, don't commence with screen

If yes, give the patient 30mls of water to drink (per procedure) and go to 42

52. Observed delayed swallowing (if the oropharyngeal transit time exceeds 2 seconds) Yes [ ] No [ ] [ ]

53. Observed presence of drooling (inability to retain the drink within the oral cavity together with pooling within the mouth and spillage from the corner of the mouth) Yes [ ] No [ ] [ ]

54. Observed cough during or within 1 min of swallowing Yes [ ]No [ ] [ ]

55. Observed dysphonia (presence of a post swallow wet or hoarse voice quality) Yes [ ] No [ ] [ ]

Interpretation of assessment

Category 1 Normal swallowing function if no abnormalities were found on assessment (Regular diet regimes)

Category 2 Mild swallowing impairment if single abnormality on bedside assessment If delayed swallowing with oropharyngeal transit time of 2-3 seconds (Modified diet of blended consistency with thickened fluids)

Category 3 Severe swallowing impairment if 2 or more abnormalities on bed side Assessment
Or significant delay in initiation of swallowing (delay arbitrarily defined as $\geq$3 seconds)
(Enteral feeding)

56. Patient's category 1 [ ] 2 [ ] 3 [ ] [ ]

57. Referred to speech therapist Yes [ ] No [ ] [ ]

58. Aspirin 150-300mg given as loading dose within 48 hours (unless contraindicated) if haemorrhagic stroke excluded Yes [ ] No [ ] [ ]

VI PAST MEDICAL HISTORY

Risk factor assessment

(Medical)

50. Did a physician ever tell you that you had a stroke?

Yes [ ][1] No [ ]2 Don't known [ ][3] [ ]

If no to (52),

51. Have you ever suffered from any of the following in a manner that was sudden?

Painless weakness on one side of your body, numbness or a dead feeling on one side

of your body, painless loss of vision in one or both eyes, inability to understand what people are saying or inability to express yourself verbally or in writing?

Yes [ ][1] No [ ][2] Don't known [ ][3] [ ]

If no or don't know go to 21

If yes, how long did the symptoms last? < 24 hrs [ ][1]> 24 hrs [ ][2] [ ]

Ever diagnosed or told that you had any of the following?

52. Hypertension: Yes [ ][1] No [ ][2] [ ]

53. Diabetes: Yes [ ][1] No [ ][2] [ ]

54. Have you ever had a blood test for your lipid levels? Yes [ ][1] No [ ][2]

Don't know [ ][3] [ ]

If yes, were they high [ ][1] Normal [ ][2] [ ]

If no go to 55

55. Have you ever had a heart attack: Yes [ ][1] No [ ][2] [ ]

56. Heart disease: Yes [ ][1] No [ ][2] [ ]

57. Heart surgery: Yes [ ][1] No [ ][2] [ ]

Lifestyle/social activities

58. Current smoking: Yes [ ][1] No [ ][2] [ ]

If yes how many cigarettes/day? 5-15 [ ][1] 16-20 [ ][2] $\geq$21 [ ][3] [ ]

If no go to 59

59. Former smoker: Yes [ ][1] No [ ][2] [ ]

60. Current alcohol consumption: Yes [ ][1] No [ ][2] [ ]

If yes go to 61

If no go to 66

61. (C) Have you ever felt you ought to cut down on your drinking?

Yes [ ]1 No [ ]2 [ ]

62. (A) Have people ever annoyed you by criticizing your drinking?

Yes [ ]1 No [ ]2 [ ]

63. (G) Have you felt guilty or bad about your drinking?

Yes [ ]1 No [ ]2 [ ]

64. (E) Have you ever had a drink first thing in the morning to steady your nerves?

Yes [ ]1 No [ ]2 [ ]

65. Interpretation: No suspected alcohol problem (all no) [ ]1

Suspected alcohol problem (one yes) [ ]2 [ ]

Alcohol abuse/ dependency (more than one yes) [ ]3

66. Are you engaged in any sport in the last 3 months? Yes [ ]1 No [ ] 2 [ ]

If yes go to 67

If no go to 69

67. About how many times a week do you engage in the sport?

Rarely [ ]1 1-2 times per week [ ]2 ≥ 3 times per week [ ]3 [ ]

68. Does this sport cause perspiration and breathlessness? Yes [ ]1 No [ ]2 [ ]

69. Any current engagement in active physical exercise that caused

perspiration and breathlessness? Yes [ ]1 No [ ]2 [ ]

If yes, specify activity-----------------------------

If no go to 71

70. About how many times a week do you engage in this activity? ------------.

Rarely [ ]1 1-2 times per week [ ]2 ≥ 3 times per week [ ]3 [ ]

71. Means of transport? Walk [ ]^{1} Bicycle [ ] ^{2} Taxi [ ]^{3} Drive [ ]^{4}

Motor cycle: Ride [ ]^{5} Ride on [ ]^{6} [ ]

Family history

Do any of your relatives suffer from or have suffered from the following conditions?

72. Stroke Yes[ ]^{1} No[ ]^{2} Don't know [ ]^{3} [ ]

If yes, relationship: Paternal [ ]^{1} Maternal [ ]^{2} Sibling [ ]^{3} [ ]

If no go to 44

73. TIA Yes [ ]^{1} No [ ]^{2} Don't know[ ]^{3} [ ]

74. High blood pressure Yes [ ]^{1} No [ ]^{2} Don't know [ ]^{3} [ ]

75. Diabetes Mellitus Yes [ ]^{1} No [ ]^{2} Don't know [ ]^{3} [ ]

76. Heart attack Yes [ ]^{1} No [ ]^{2} Don't know [ ]^{3} [ ]

77. Heart disease Yes [ ]^{1} No [ ]^{2} Don't know [ ]^{3} [ ]

78. Heart surgery Yes [ ]^{1} No [ ]^{2} Don't know [ ]^{3} [ ]

79. High blood cholesterol. Yes[ ]^{1} No [ ]^{2} Don't know [ ]^{3} [ ]

Current medications.

80. Anti-hypertensive drugs Yes [ ]^{1} No [ ]^{2} [ ]

If yes medication Regular [ ]^{1} Irregular [ ]^{2} [ ]

81. Antiplatelet drugs Yes [ ]^{1} No [ ]^{2} [ ]

82. Anticoagulants Yes [ ]^{1} No [ ]^{2} [ ]

83. Oral/ injectable contraceptives Yes [ ]^{1} No [ ]^{2} [ ]

84. Lipid lowering drugs Yes [ ]^{1} No [ ]^{2} [ ]

85. Anti-diabetic drugs Yes [ ]^{1} No [ ]^{2} [ ]

VII PHYSICAL EXAMINATION

Baseline physiologic parameters Time ---------------------

86. Temperature [ ]^{0} C Febrile [ ][1] Afebrile [ ] [2] [ ]

87. Blood pressure ------/----- Systolic/ Diastolic, ----/---- Systolic/ Diastolic,

Average ----/---- Systolic/ Diastolic, >140/90mmHg Yes [ ][1] No [ ][2] [ ]

88. Level of consciousness (GCS) …….. Normal [ ] [1] Impaired [ ][2] [ ]

89. Hydration status Good [ ] Dehydrated [ ] [ ]

90. Finger stick blood glucose --------- <10mmol/L [ ] >10mmol/L [ ] [ ]

Cardiovascular examination

91. Pulse rate: [ ] bpm. Nature Regular [ ] [1] Irregularly Irreg [ ][2] [ ]

92. Carotid Bruits: Yes [ ][1] No [ ][2] [ ]

93. Evidence of valvular pathology Yes [ ][1] No [ ][2] [ ]

94. Apex displaced Yes [ ][1] No [ ][2] [ ]

Neurological exam:

95. Neuro deficit present Yes [ ][1] No [ ][2] [ ]

96. If yes, specify Motor [ ][1] Sensory [ ][2] Global [ ][3] [ ]

97. Dysphasia Yes [ ][1] No [ ][2] [ ]

If yes, specify Motor [ ][1] Sensory [ ][2] Global [ ][3] [ ]

98. Hemi- inattention Yes [ ][1] No [ ][2] Difficult to assess [ ][3] [ ]

99. Evidence of visual field defect Yes [ ][1] No [ ][2] Difficult to assess [ ][3] [ ]

Peripheral Nervous system:

Motor examination

100. Muscle bulk Normal [ ][1] Reduced [ ][2] [ ]

If reduced specify limb: RT upper [ ][1] RT lower [ ][2]

LT upper [ ][3] LT lower [ ][4] [ ]

101. Power 0/5 1/5 2/5 3/5 4/5 5/5

Difficult to assess Yes [ ][1] No [ ][2] [ ]

If difficult to assess go to 102, if not

Specify site/limb. RT upper [ ][1] RT lower [ ][2] [ ]

LT upper [ ][3] LT lower [ ][4] [ ]

102. Reflexes: Normal [ ][1] Hyporeflexia [ ][2] Hyper-reflexia [ ][3] Clonus [ ][4] [ ]

Specify site/limb. RT upper [ ][1] RT lower [ ][2] [ ]

LT upper [ ][3] LT lower [ ][4] [ ]

103. Tone: Normal [ ][1] Hypotonia [ ][2] Hypertonia [ ][3] [ ]

Specify site/limb. RT upper [ ][1] RT lower [ ][2]

LT upper [ ][3] LT lower [ ][4] [ ]

Sensory examination

104. Sensory impairment: Yes [ ][1] No [ ][2] Difficult to assess [ ][3] [ ]

If no or difficult to assess go to 105

If yes, specify mode of impairment.

Light touch Yes [ ][1] No [ ][2] [ ]

Pain Yes [ ][1] No [ ][2]

105. Scandinavian stroke scale score …….. Mild [ ] Severe [ ] [ ]

VIII. LABORATORY RESULTS.

Complete blood count and ESR

106. Haemoglobin. ------- g/dl. Normal [ ][1] High [ ][2] Low [ ][3] [ ]

107. Platelet counts---------------- Normal [ ][1] High [ ][2] Low [ ][3] [ ]

108. Total WBC count: -------- Normal [ ][1] High [ ][2] Low [ ][3] [ ]

109. ESR Normal [ ][1] High [ ][2] Low [ ][3] [ ]

110. RPR . Reactive [ ][1] Non-reactive [ ][2] [ ]

111. HIV serology Reactive [ ][1] Non-reactive [ ][2] [ ]

Lipid profile (mg/dl).

112. Total cholesterol……….. < 200 [ ][1] 200-239 [ ][2] > 240 [ ][3] [ ]

113. LDL cholesterol……….. < 100 [ ][1] 100-129 [ ][2] 130-159 [ ][3] [ ]

160-189 [ ] [4] ≥190 [ ][5]

114. HDL cholesterol………< 40 [ ][1]≥ 60 [ ][2] [ ]

115. Triglycerides……. <150 [ ][1] 150-199 [ ][2] 200-499 [ ][3]≥ 500 [ ][4] [ ]

Blood sugar

116. Fasting finger stick sugar: ---------- mg/dl < 126 [ ][1]≥126 [ ][2] [ ]

117. Venous fasting blood sugar ………… mg/dl <126 [ ][1]>126[ ][2] [ ]

IX. IMAGING INVESTIGATIONS

Echocardiography/ Doppler ultrasound of carotid arteries/ 12 Lead ECG

118. Evidence of cardioembolic source if any of these is present. Akinetic wall motion, cardiac thrombi, vegetations, VEF of ≤35%, multiple valvular involvement, severe valvular lesions (specify),

If yes state which one-------------- Yes [ ][1] No [ ][2] [ ]

119. Evidence of carotid artery stenosis, Atherosclerosis, Atheromatous plagues,

Yes [ ][1] No [ ][2] [ ]

If yes specify--------------------------

120. Atrial fibrillation on ECG Yes [ ] No [ ] [ ]

CT scan of brain, if hemorrhagic, go to 124

121. CT scan if ischemic stroke Single lesion [ ] Multiple [ ] [ ]

122. CT scan if ischemic stroke, infarct size …………… (mm)

123. Subtype of ischemic stroke

Lacunar [ ][1] Embolic [ ][2] Atherosclerotic [ ][3] Unspecified [ ] [ ]

124. CT scan if haemorrhagic stroke

Subarachnoid haemorrhage [ ][1] Intracerebral haemorrhage [ ][2]

Both [ ][3] [ ]

X THIRTY DAY FOLLOW UP

125. Length of hospital stay ………..

126. Died during hospitalization Yes [ ] No [ ] [ ]

If yes go to 131

127. Date of discharge …………………………

128. Review date ………………………….

129. Date of notification a week to review date …………………….

130. Notification successfully done yes [ ] No [ ] [ ]

131. Thirty day outcome Alive [ ][1] Dead [ ][2] [ ]

If alive go to 133

132. When died Date …………………… N^{th} day ………

133**.** Barthel index score …… Independent if $\geq$95 [ ] Dependent <95 [ ] [ ]

134. Modified Rankin Score ……. Mild disability (0-2)[ ] Moderate (3-4) [ ]

Severe (5) [ ] [ ]

APPENDIX 11 (a): CONSENT FORM (HOSPITAL STUDY-INTERVENTION)

Study title: Improving stroke prevention and outcomes in Uganda: Population based survey in urban and rural Wakiso and hospital study at Mulago hospital

Principal Investigator: Nakibuuka Jane, Department of Medicine, School of Medicine, Makerere University College of Health Sciences Tel: 0772618111

To be read by/read out to potential participants ***aged 18 years or older***

Before signing this form, read and consider the following information given to you regarding the reasons for the study, procedures, benefits and any other important information. Please ask the interviewer if some of the information is not clear.

1. Why is this study being done?

Stroke is becoming common and a major cause of poor outcomes such as death and disability in low income countries such as Uganda. The exact care given to patients suspected of having stroke and the factors associated with poor outcomes are not well studied in our setting, yet this is very crucial for planning health services. We plan to conduct a study to determine the effect of implementing a set stroke management plan in addition to whatever clinical or neurological care that is usually received by patients with stroke and the factors associated with poor outcomes in order to inform relevant stake holders on planning better health services to treat and prevent stroke.

2. Why have I been chosen to take part in the study?

You have been selected because you have been found to have a stroke and you have presented to Mulago hospital during a time when this study is taking place. We will be asking 169 other men and women to participate in this study. If this is your first time to participate in such a study, use this opportunity to familiarise yourself with the study and ask any questions that you may have.

3. What will happen to me if I decide to take part in the study?

If you choose to take part in the study, you will be expected to answer questions about your current and past medical illness in relation to stroke and its risk factors. You will be examined too. We will request you to stop feeding for at least 8 hours overnight so that we can obtain a 6ml blood sample from a vein on your arm the following morning. This will be done is a proper way using a sterile needle and syringe. The blood that we collect will be placed in a sample bottle and sent to our laboratory for testing. We will test for blood fat levels, complete blood count and syphilis. If your HIV status is not known, results of the blood test will be obtained from the nursing sister in-charge of the HIV routine counselling and testing programme on your ward. Imaging studies like chest x-ray, echocardiography and doppler ultrasound of the carotid arteries may also be done if you are found to have a body temperature higher than normal or if examination of your heart is found to be abnormal, respectively. All the results of the tests will be returned and placed in your file. They will be

used in deciding your care by the doctors caring for you on the ward. These results will be given to you too if you wish to know them.

4. Can I refuse to take part in the study or stop being in the study?

It is up to you to decide on participating in the study or not. You can also stop being in the study at any time you want without giving a reason. Your refusal to participate in the study will not in any way impact regular clinical care given to you.

5. What risks can I expect from being in the study?

If you take part in the study, the risks to you are very slight. The procedure of drawing blood from your veins may cause some pain and discomfort. You may bleed a little from the site, however the amount of blood drawn will be too small to pose any health hazard. By participating in this study, you may lose some privacy, but as the investigator, I assure you that all members of the study team will do their utmost to make sure that all records are kept confidential. We will use a code number to identify you and all records will be kept in a locked secure setting. Your identity will not be used in any reports, presentations at scientific meetings or publications resulting from the study.

6. Are there any benefits to me from participating in the study?

You will be interviewed, examined and investigated for major risk factors for stroke as well as factors known to cause poor outcomes after stroke. Just as CT scan of the brain was done at no cost to you, like wise all these investigations will be done at no cost to you. Results of physical measurements and tests will be placed in your ward file to help us manage your condition well. From information obtained from this study, it may be possible to contribute towards improving stroke prevention and poor outcomes such as death.

7. For how long will I be followed up if I decide to participate in the study?

You will be followed up for a period of 30 days. We would however also like to follow you up for a period of up to 1 year to know how you are doing. If you agree to this additional follow up period, you will be required to sign another statement of consent at the end of this form.

8. Whom can I ask if I have any questions about the study?

I would like to answer any questions at any time about this research study. If you have any now, please ask. However, if you have any questions in future you can contact me at the Department of medicine, Mulago hospital. Tel: 0772618111

For any concerns about your rights in this study, please contact the chairman of Makerere School of Medicine Research and Ethics Committee on Tel: (+256) 0414-533541

9. Statement of consent

The nature and purpose of this study has been explained to me and I understand it. I therefore agree to participate in the study.

Name of patient…………………….. ……Signature……………………Date………...

Name of investigator……………………...Signature…………………....Date……….....

Name of witness……………………….......Signature……………………Date……….....

10. Statement of consent for a one year follow up period

The nature and purpose of this study has been explained to me and I understand it. I therefore agree to be followed up for a period of one year.

Name of patient…………………….. ……Signature……………………Date………...

Name of investigator……………………...Signature…………………....Date……….....

Name of witness……………………….......Signature……………………Date……….....

APPENDIX 11 (b): CONSENT FORM FOR HOSPITAL STUDY (INTERVENTION STAGE) IN LUGANDA

Study title: Improving stroke prevention and outcomes in Uganda: Population based survey in urban and rural Wakiso and hospital study at Mulago hospital

Akulira okunoonyereza: Nakibuuka Jane, asangibwa mu Department of Medicine, School of Medicine, Makerere University College of Health Sciences ku ssimu: 0772618111

To be read by/read out to potential participants ***aged 18 years or older***

Nga tonaba kuteeka mukono ku kiwandiiko kino, ssoma era olowooze ku bwino ono akuweebwa akwata ku nsonga lwaki omusomo gukolebwa, emitendera gyagwo, ebiguganyurwamu ne bwino omulala omukulu. Tukusaba obuuze oyo muntu abuuza ebibuuzo bwekiba ng'omo ku bwino akuweebwa ategerekeka bulungi..

1. Lwaki omusomo guno gukolebwa?

Obulwadde bwa situlooko butandise okulabibwa ennyo era nga bukulu mu kuleetawo ebiva mu kulwala ebibi ng'okufa n'okulemala mu nsi zimufuna mpola nga Uganda. Okulabirirwa n'obujjanjabi obuweebwa abalwadde abateeberezebwa okuba n'obulwadde bwa situlooko era n'esonga ezivaako ebiva mu kulwala ebibi tebisomedwako bulungi mu mbeera z'ebitundu byaffe songa kikulu nnyo mu kutegeka empereza ez'ebyobulamu. Tutegeka okukola omusomo okulaba biki ebiva mu kuteeka mu nkola pulaani entegekere dala ey'okukwatamu obulwadde bwa situlooko ng'ogaseeko n'okulabirirwa n'obujjanjabi mu kiriniki n'obw'enkola y'obusimu obuyamba omuntu okutambuza ebitundu by'omubiri ebyenjawulo n'obwongo okukola obulungi (clinical or neurological care) obufunibwa bulijjo abalwadde ba situlooko era n'ensonga ezekuusa ku biva mu kulwala ebibi kisobozese okubuulira beekikwatako ku kutegeka empereza ez'ebyobulamu ezisingako obulungi mu kujjanjaba era n'okuziyizaamu obulwadde bwa situlooko.

2. Lwaki nondeddwa okwetaba mu musomo guno?

Olondedwa kubanga olina obulwadde bwa situlooko era ng'oze mu dwaliro e mulago mu kiseera omusomo mwegukolebwa. Tujja kuba nga tusaba abaami n'abakyala kikumi mu nkaaga mu mwenda (169) okwetaba mu musomo guno. Bweguba nga gwe mulundigwo ogusooka okwetaba mu musomo nga guno, kozesa omukisa guno okutegeera ebikwata ku musomo era obuuze ebibuuzo byonna byoyinza okuba nabyo.

3. Kiki ekinambaako singa nsalawo okwetaba mu musomo?

Bwosalawo okwetaba mu musomo, ojja kuba osuubirwa okuddamu ebibuuzo ebikwata ku ndwadde zo zolina kati n'ezemabega ezekuusa ku bulwadde bwa situlooko n'ebyo ebiteeka omuntu mu kabegye k'okumufuna era okeberebwe. Tujja kukusaba oleme kulya okumala essaawa munaana okuyita mukiro tusobole okufuna ogumu ku musaayi/sampo y'omusaayi eweza miiru mukaaga okuva ku musuwa gw'okumukono enkeera kumakya. Kino kijja kukolebwa mu ngeri entuufu nga tukozesa empiso n'ebomba empya okutali buwuka.

Omusaayi gwetukungaanya gujja kuteekebwa mu kaccupa omuteekwa sampo era gusindikibwe mu laabu awakeberebwa omusaayi gukeberebwe. Tujja kukebera okulaba obungi bwa masavu g'omumusaayi, okulaba obungi bwe birungo ebisangibwa mu musaayi mubujuvu bwabyo era ne kabotoogo. Bwoba nga bwoyimiride ku byakawuka ka siriimu tebimanyidwa, ebinaava mu kukebera omusaayi bijja kufunibwa okuva ku akulira ba nansi ku waadiyo abali ku pulogulaamu y'okubudabuda n'okukebera kwa kawuka ka siriimu okukolebwa ku buli ajja kuwaadi.Okusoma kw'ebifanaanyi okugeza ng'eby'enkola y'omutima ebikozesa ejjengo lya masanyalaze kyetuyita echocardiography n'okukubibwa kwekifananyi ekiraga entambula y'omusaayi mu misuwa ekiyitibwa Doppler ultrasound eneekolwa ku busuwa obuyitibwa carotid arteries ebiyinza okukolebwa singa ebbugumu mu mubirigwo lyeyongera okussuka ku lya bulijjo ne singa omutima gwo gusangibwa okuba nga ssimulamu bulungi. Byonna ebinaava mu kukeberebwa bijja kuddizibwa era biteekwe mu fayiro yo. Era bijja kukozesebwa mu kusalawo ku bujjanjabi bwo abasawo abakulabirira ku waadi. Ebinaava mu kukebera bijja kukuweebwa bwoba ng'oyagala okubimanya

4. Nyinza okugaana okwetaba mu musomo oba nendekeraawo okwetaba mu musomo?
Kiri gyoli okusalawo okwetaba mu musomo oba nedda. Era osobola okulekeraawo okubeera mu musomo essaawa yonna woyagalira nga towadde nsonga yonna**.** Okugaana kwo okwetaba mu musomo tekujja kukossa kulabirirwako okwabulijjo okukuweebwa mu byobujjanjabi

5. Buzibu ki bweba nsuubira obuva mukwetaba mu musomo?
Bwewetaba mu musomo guno obuzibu bwoyolekede butono nyo. Omutendera ogw'okujjibwako omusaayi okuva ku misuwa gyo kiyinza okukuleetera obulumi obusaamusaamu n'okutawanyizibwa. Oyinza okufulumya omusaayi omutono okuva mukifo ekyo, naye ng'omusaayi ogunafunibwa gujja kuba mutononyo okubaako akabegye gweguteeka ku bulamubwo. Bwewetaba mu musomo guno, oyinza okufiirwa ebimu ku byamabyo, naye nze nga anoonyereza, nkukakasa nti bonna abali ku tiimu y'omusomo bajja kukola kyona ekisoboka okulaba nti likodizo zonna/ebiwandiikobyo byonna eby'ekisawo bijja kukuumibwa nga byakyama. Tujja kukozesa ennamba enekusifu/koodi okusobola okukumanya era nga likodizo/ebiwandiikobyo bijja kuterekebwa awantu awesigika awasibwa n'ekisumuluzo. Ebikumanyisa tebijja kukozesebwa mu lipoota zonna, bunayanjurwa oba ebinafulumizibwa mu bitabo nga biva mu musomo

6. Waliwo byeganyurwa mu kwetaba mu musomo?

Ojja kubuuzibwa ebibuuzo, wekebejjebwe era obe n'okeberebwa ku bintu ebisinga okuteeka abantu mu kabegye k'okufuna obulwadde bwa situlooko n'ensonga/ebintu ebimanyidwa ebileetawo okuba n'ebiva mu kulwala ebibi ng'omaze okufuna situlooko. Nga okukubibwa ekifananyi/sikaani eyitibwa CT scan ey'obwongo eyakolebwa awatali kufulumya ssente gyoli, nabwekityo byonna bino ebinakeberebwa bijja kukolebwa awatali kufulumya ssente yonna gyoli. Ebinaava mu kupimibwa ku mubiri n'ebikeberebwa bijja kuteekebwa mu fayiroyo ey'okuwaadi kiyambe abasawo okukukwata embeerayo mu ngeri esingako okuba ennungi. Okuva mu bwino afunidwa okuva mu musomo guno, kisoboka okubaako kyekiyamba mu kwongera okulongoosa mu ngeri obulwadde bwa situlooko gyebuziyizibwamu n'ebibuvaamung'okufa.

7. Okwetaba mu musomo guno kinatwala banga ki?

Kijja kutwala ebanga lya naku asatu. Twandiyagadde okumanya bwoli natte okutusa ebanga lya mwaka gumu kasokedde nga ofuna obulwadde buno obwa situlooko. Singa okiriza okusaba kwaffe kuno, ojja kusabibwa okuteka omukono gwo ku sitaatimenti endala eya obweyamo buno kunkomelero ya form eno.

8. Aani gwenyiza okubuuza singa nina ebibuuzo ebikwata ku musomo?

njagala okukuddamu ebibuuzo byonna byoyinza okuba nabyo essaawa yonna ebikwata ku musomo guno ogw'okunoonyereza. Bwoba n'ekyolina kyonna essaawa eno, tukusaba obuuze. Naye bwoba n'ebibuuzo gyebujja mumaaso oyinza okuntuukirira mu dipatimenti/kitongole kyabasawo eky'edwaliro ly'emulago (Department of medicine, Mulago hospital) ku ssimu: 0772618111

Ku bikwata ku kyewebuuza ku ddembelyo mu musomo guno, tukusaba otuukirire ssentebe wa kakiiko ak'ettendekero ly'ekisawo eMakerere akavunanyizibwa okulaba nti eddembe ly'abanoonyerezebwako terityoboolwa mu misomo egikolebwa akayitibwa Makerere School of Medicine Research and Ethics Committee ku ssimu: (+256) 0414-533541

9. Sitaatimenti ey'obweyamwo

Ekika n'ekigendererwa ky'omusomo guno binyinyonyodwa gyendi era bitegede bulungi. Era kati nzikiriza okwetaba mu musomo.

Erinya ly'omulwadde…………………Omukono……….......Ennaku z'omwezi …........

Erinya ly'anoonyereza…………………….Omukono……………Ennaku z'omwezi …….

Erinya ly'omujjulizi……………………Omukono………………Ennaku z'omwezi….....

10. Sitaatimenti ey'obweyamwo okwetaba mu musomo guno okumala omwaka gumu

Ekika n'ekigendererwa ky'omusomo guno binyinyonyodwa gyendi era bitegede bulungi. Era kati nzikiriza okwetaba mu musomo okumala omwaka gumu.

Erinya ly'omulwadde…………………Omukono……….......Ennaku z'omwezi …........

Erinya ly'anoonyereza………………..Omukono……………....Ennaku z'omwezi …….

Erinya ly'omujjulizi…………………..Omukono………………Ennaku z'omwezi….....

APPENDIX 12: SURVEY QUESTIONNAIRE

Improving stroke prevention and outcomes in Uganda: Population survey and hospital based study

Participant ID Initials

				-		

Section 1: Demographics

1.1 Interviewer name & code no.…………………………………|__|__| INTCODE

1.2 Date of interview: ☐☐ / ☐☐ / ☐☐☐☐
dd mm yyyy

1.3 Residence codes: |__|__| VNO |__|__|__| HNO

1.4 Was written Informed Consent obtained? ☐ No ☐ Yes *If no, please do not proceed.*

1.5 Was consent for sample storage obtained? ☐ No ☐ Yes

1.6 Gender: ☐ Male ☐ Female

1.7 Date of birth: ☐☐ / ☐☐ / ☐☐☐☐
dd mm yyyy

If year of birth not known ask or estimate age (years) |__|__| AGE

1.8 Marital status: ☐ Married ☐ Single- never married ☐ Divorced ☐ Separated ☐ Widowed

1.9 Religion:☐ Catholic ☐ Protestant ☐ Muslim ☐ Pentecostal ☐ Traditional ☐ Other………..

1.10 Highest level of education attained: ☐ Non ☐ Primary (P1-7) ☐ Secondary (S1-6) ☐ Tertiary

Codes: 1=Yes, 2=No, 7=Refused, 8/88/888= Don't know, 9/99/999=No information/Missing Unless otherwise specified

Section 2: Perceptions of stroke

Section 2a: What do you believe causes a stroke?

Please tick all that applies

☐ Demons	☐ hypertension	☐ don't know
☐ Witch craft	☐ cigarette smoking	☐ Bad diet
☐ God's will	☐ Fatty foods	☐ alcohol
☐ Atherosclerosis	☐ high cholesterol	☐ Stress
☐ Angry ancestral spirits	☐ genetics (hereditary)	☐ Obesity
☐ Oral contraceptives	☐ lack of exercise	☐ Inheritance

☐ Others (please specify)……………………………………………………

Section 2b: What would be your planned response to an event of stroke?

☐ Call general practitioner or family doctor
☐ Ask family members or relatives to help
☐ Go to chemist for advice or medication
☐ Self-medication
☐ Ask friend or neighbours for help
☐ Go to hospital
☐ Visit community health centre
☐ Visit alternative health care providers (herbal med, traditional healers),
☐ Seek spiritual healing (prayer)
☐ Combination of hospital and tradition
☐ Combination of hospital and faith
☐ Invite a Physiotherapist
☐ Others (please specify)

Section 2c: Concern about having a stroke

2c.1 What is the possibility of you having a stroke in the next 12 months?
☐ No chance ☐Low chance ☐ Moderate chance ☐ High chance ☐ Never thought about stroke before

2c.2 What is the possibility of you having a stroke in your life time?
☐ No chance ☐Low chance ☐ Moderate chance ☐ High chance ☐ Never thought about stroke before

Section 3: Knowledge of stroke

Section 3a: Basic knowledge of stroke

3a.1 What organ of the body is affected by stroke: ☐ Brain ☐ Heart
☐ Kidney ☐ Liver
☐ Lungs ☐ Don't know ☐ Other…………………

3a.2 Is stroke preventable? :☐ Yes ☐ No

3a.3 Can a person have stroke more than once? :☐ Yes ☐ No

3a.4 Does stroke have an effect on daily activities like driving a car, dressing, use of the toilet and having a job? : ☐ Yes ☐ No

3a.5 Can stroke be prevented if treated early? : ☐ Yes ☐ No

Section 3b: Knowledge of stroke risk factors

3b.1 Do you know any risk factors for stroke? ☐ Yes ☐ No

3b.2 If Yes, what are the risk factors for stroke that you know of? Please tick all that applies

☐ Old age ☐ hypertension
☐ Diabetes ☐ cigarette smoking
☐ Heart disease ☐ alcohol
☐ Atherosclerosis ☐ high cholesterol
☐ Obesity ☐ genetics (hereditary)
☐ Stress ☐ lack of exercise
☐ Poor hygiene ☐ headache or migraine
☐ Cancer ☐ oral contraceptives
☐ Bad diet ☐ tremors
☐ Other (please specify)………………………………………

Section 3c: Knowledge of stroke warning signs

3c.1 Do you know any warning signs of stroke? ☐ Yes ☐ No

3c.2 If Yes, what is the warning sign of stroke that you know of? Please tick all that applies

☐ Dizziness ☐ blurred or double vision or loss of vision
☐ Headache ☐ sudden difficulty in speaking or understanding or reading
☐ Tiredness ☐ fever/sweating
☐ Shortness of breath ☐ Chest pain or chest tightness
☐ Nausea/vomiting ☐ weakness of any part of the body
☐ Weakness of one side of the body ☐ paralysis of any part of the body
☐ Paralysis of one side of the body ☐fainting black out collapse
☐ Numbness tingling sensation or dead sensation of any body part
☐ Numbness tingling sensation or dead sensation of one side of the body
☐Others (please specify)…………...

3d: Sources of information about stroke

What are your sources of information about stroke? Please tick all that applies
☐ Health care providers ☐ Friends and relatives
☐ Radio ☐ TV ☐ News papers
☐ Electronic media ☐ Others (please specify)……………

Section 4: Prevalence of risk factors of stroke

4a: Does the participant have present medical history for symptoms suggestive of a stroke?

		Yes	No	If yes, Duration
1	Sudden trouble walking, dizziness, loss of balance or coordination	☐	☐	
2	Sudden weakness of the face, arm or leg especially on one side of the body<br>If yes, specify which side Right ☐ Left ☐<br>If no, go to 3	☐	☐	
3	Sudden numbness of the face, arm or leg especially on one side of the body<br>If yes, specify which side Right ☐ Left ☐<br>If no, go to 4	☐	☐	
4	Sudden confusion	☐	☐	
5	Sudden trouble speaking or understanding	☐	☐	
6	Sudden severe headache with no known cause	☐	☐	
7	Sudden onset of vomiting with no associated nausea	☐	☐	
8	If yes to any of the above<br>Did you lose consciousness	☐	☐	
9	If yes to any of the above<br>Did you have convulsions	☐	☐	

4b; Does the participant have past medical history for risk factors of a stroke? Please review the participant's medical records for more information.

		Yes	No	Unknown
1	Did a physician ever tell you that you have a stroke<br>If yes, go to 3	☐	☐	☐
2	Have you ever suffered from any of the following in a manner that was sudden? Painless weakness on one side of your body, numbness or a dead feeling on one side of your body, painless loss of vision in one or both eyes, inability to understand what people are saying or inability to express yourself verbally or in writing?<br>If yes, how long did the symptoms last	☐<br><24hrs<br>☐☐	☐<br>>24hrs	☐
3	Ever diagnosed or told that you have diabetes	☐	☐	
4	Ever diagnosed or told that you have hypertension	☐	☐	
5	Have you ever had a blood test for you lipid levels (Body fat)<br>If yes, were they high	☐<br>☐	☐<br>☐☐	☐
6	Have you ever had a heart attack	☐	☐	☐
7	Ever been diagnosed or told that you have heart disease	☐	☐	☐
8	Ever had heart surgery	☐	☐	☐
9	sickle cell disease	☐	☐	☐
10	HIV/AIDS	☐	☐	☐

4c: Lifestyle/social activities

1 Tobacco use

Current use		☐ Yes	☐ No
If yes, what type of tobacco	Cigarettes	☐	Other ☐
How many cigarettes per day	5-15 ☐	16-20 ☐	≥21 ☐
Former smoker		☐ Yes	☐ No

2 Alcohol use

Current alcohol consumption ☐ Yes ☐ No

<u>CAGE screening questions</u>

(1) Have you ever felt you ought to cut down on your drinking? ☐ Yes ☐ No

(2) Have people ever annoyed you by criticising your drinking? ☐Yes ☐ No

(3) Have you ever felt guilty or bad about your drinking? ☐ Yes ☐ No

(4) Have you ever had a drink first thing in the morning to steady your nerves?

☐ Yes ☐ No

Interpretation; No suspected alcohol problem (if answer to all is no) ☐

Suspected alcohol problem (if one answer is yes) ☐

Alcohol abuse/dependency (if more than one answer is yes) ☐

3 physical activity assessments

Are you engaged in any sport in the last 3 months? ☐ Yes ☐ No

If yes,

About how many times a week do you engage in the sport?

Rarely ☐ 1-2 times per week ☐ ≥ 3 times per week ☐

Does this sport cause perspiration and breathlessness? ☐Yes ☐ No

Any current engagement in active physical exercise that caused

perspiration and breathlessness? ☐Yes ☐No

If yes, specify activity …………………………………………………..

If no
About how many times a week do you engage in this activity?
Rarely ☐ 1-2 times per week ☐ ≥ 3 times per week ☐
Means of transport? Walk ☐ Bicycle ☐ Taxi ☐Drive☐
Motorcycle Ride☐ Ride on☐

4 Diet assessments

1. In a typical week, on how many days do you eat fruit, such as pineapple, mango, jackfruit, and passion fruit?
(Give number of **days**: 88 = don't know, 99 = refused to answer) |__|__| DIET1
If 0 days, go to

2. How many servings of fruit do you eat on one of those days?
(Give number of **servings**: 88 = don't know, 99 = refused to answer) |__|__| DIET2

3. In a typical week, on how many days do you eat vegetables such as tomato, cabbage, greens, carrots, and eggplant?
(Give number of **days**:88= don't know, 99= refused to answer) ....|__|__| DIET3

If 0 days, go to question 5

4. How many servings of vegetables do you eat on one of those days?
(Give number of **servings**:88=don't know,99= refused to answer) ...|__|__| DIET4

5. In a typical week, on how many days do you eat starchy staples, such as posho, cassava, sweet potato and rice?
(Give number of **days**: 88 = don't know, 99 = refused to answer) |__|__| DIET5

If 0 days, go to question 7

6. How many servings of starchy staples do you eat on one of those days?
(Give number of **servings**:,88 = don't know, 99 = refused to answer) |__|__| DIET6

7. In a typical week, on how many days do you eat matooke?
(Give number of **days**: 88 = don't know, 99 = refused to answer) |__|__| DIET10

If 0 days, go to question 9

8. How many servings of matooke do you eat on one of those days?
(Give number of **servings**: 88 = don't know, 99= refused to answer) |__|__| DIET11

9. What type of oil or fat is most often used for food preparation in the household?
1 = Vegetable oil 2 = Animal fat
3 = Butter 4 = Margarine,
5 = Other 6 = None in particular
7 = None used 88=Don'tknow
|__| DIET12
If other type of fat or oil, specify________________________________ DIET12SP

10. When did you last eat anything? |__|__|: |__|__| DIET7

HR MIN (Give duration in **hours** and **minutes**)

11. When did you last drink anything, except water? HR MIN (Give duration in **hours** and **minutes**) |__|__|: |__|__| DIET8

12. Can you estimate how much salt is added to your food during cooking?
Unit: 1= Palm, 2=Teaspoon, 3=Pinch |__| DIET9U
Quantity: 00 = none 88 = don't know |__|__| DIET9

4d: Family history assessment

Is the participant aware of presence of the following conditions among family members?

	**Absent Present Unknown**		
Stroke	☐	☐	☐
TIA	☐	☐	☐
Diabetes	☐	☐	☐
Hypertension	☐	☐	☐
Heart attack	☐	☐	☐
Other heart diseases	☐	☐	☐
High body fat	☐	☐	☐

4e: Medication history

Record all the drugs the participant is currently using

Antihypertensives: ☐ captopril ☐ atenolol ☐nifedipine ☐aprinox ☐ Others…

Antiplatelet agents: ☐ aspirin ☐clopidogrel ☐ Others…………..

Anticoagulants: ☐ warfarin ☐heparin ☐ Others…………..

Antithrombotic: ☐streptokinase ☐ Others…………..

Lipid lowering drugs: ☐ atovastatin ☐ fenfibrate ☐ simvastatin ☐ Others…………..
Past and /or current oral contraceptives (women only):
☐Oral contraceptives ☐ Injectable contraceptives

4f: Physical assessment

1 Height:cm ☐☐☐

2 Weight: ☐☐☐.☐ kg

3 Waist circumference: ☐☐☐

4 Hip circumference: ☐☐☐

5 Heart rate: ☐☐☐ bpm

6 Blood pressure (systolic/diastolic): ☐☐☐ / ☐☐☐ mm/Hg

4g Laboratory tests and results

Sample date: ☐☐ / ☐☐ / ☐☐☐☐ ☐ Not done

dd mm yyyy

CHEMISTRY			
	Value	Unit	
LDL-cholesterol		☐ mg/dL	
HDL-cholesterol		☐ mg/dL	
Triglycerides		☐ mg/dL	
Total Cholesterol		☐ mg/dL	
Blood sugar		☐ mg/dL	☐ mmol/l

SYPHILIS TESTING	
RPRG	☐ neg ☐ pos

HIV TESTING	
HIV test	☐ neg ☐ pos

APPENDIX 13 (a): CONSENT FORM FOR SURVEY

Study title: Improving stroke prevention and outcomes in Uganda: Population survey and hospital study

Principal Investigator: Nakibuuka Jane, Department of Medicine, School of Medicine, Makerere University College of Health Sciences Tel: 0772618111

*To be read by/read out to potential participants **aged 18 years or older***

Before signing this form, read and consider the following information given to you regarding the reasons for the study, procedures, benefits and any other important information. Please ask the interviewer if some of the information is not clear.

1. Why is this study being done?

Risk factors for stroke such as hypertension and diabetes are reported to be common and a major cause of stroke. Stroke is becoming common and a major cause of poor outcomes such as death and disability in low income countries such as Uganda. The exact burden of stroke and its risk factors is not well studied in our setting, yet this is very crucial for planning health services. What the public understands or knows about stroke and its risk factors and what they do in the event of a stroke is also very important in preventing stroke and the poor outcomes of death and disability. We plan to conduct a study to determine the burden and risk factors for stroke and what the communities know and understand about stroke in order to inform relevant stake holders such as the government on planning better health services to prevent and treat these diseases.

2. Why have I been chosen to take part in the study?

You live in urban or rural area of Wakiso district during the time when this study is being conducted and your household has been selected. We will be asking 3802 men and women to participate in this study. If this is your first time to participate in such a study, use this opportunity to familiarise yourself with the study and ask any questions that you may have.

3. What will happen to me if I decide to take part in the study?

If you choose to take part in the study, you will be expected to answer questions about your current and past medical illness in relation to stroke and its risk factors such as if you have suffered from a stroke before, if you have hypertension, if you smoke, exercise among others. You will be examined too and we will among others measure your blood pressure, how much you weigh and how tall you are.

We will request you to stop feeding for at least 8 hours overnight so that we can obtain a blood sample from a vein on your arm the following morning. This will be done is a proper way using new sterile needles and syringes. The blood that we collect will be placed in a sample bottle and sent to our laboratory for testing. We will test for blood fat levels, complete blood count and syphilis.

The results of the tests will be given to you if you wish to know them and the study staff will explain what your results mean. If you do not collect your results, we would like to contact

you if your results are abnormal. Depending on your results, they will advise you on whether further testing is needed as well as recommend changes to your lifestyle or treatment to help keep you and your family prevent stroke and diseases that put you at high risk for stroke.

4. Can I refuse to take part in the study or stop being in the study?

It is up to you to decide on participating in the study or not. You can also stop being in the study at any time you want without giving a reason.

5. What risks can I expect from being in the study?

If you take part in the study, the risks to you are very slight. The procedure of drawing blood from your veins may cause some pain and discomfort. You may bleed a little from the site, however the amount of blood drawn will be too small to pose any health hazard. By participating in this study, you may lose some privacy, but as the investigator, I assure you that all your medical records will be kept confidential. We will use a code number to identify you and all records will be kept under lock and key. Your identity will not be used in any reports or publications resulting from the study.

6. Are there any benefits to me from participating in the study?

You will be interviewed, examined and investigated for major risk factors for stroke as well as factors known to cause poor outcomes after stroke at no cost to you. When your results are returned to you, you will receive advice of behaviours of lifestyle and treatment which will improve your health. Information obtained from you and other participants will help the researchers to lobby government for better health services to prevent and treat stroke.

7. Whom can I ask if I have any questions about the study?

The research team members would like to answer any questions that you may have at any time about this research study. If you have any now, please ask. However, if you have any questions in future you can contact me at the Department of medicine, Mulago hospital. Tel: 0772618111 or the Principal investigator of the Cardio-vascular disease survey Dr. Mondo Charles, Uganda Heart Institute, Mulago Hospital Tel: +256-774460496

For any concerns about your rights in this study, please contact the chairman of Makerere School of Medicine Research and Ethics Committee on Tel: (+256) 0414-533541

8. Statement of consent

The nature and purpose of this study has been explained to me and I understand it. I therefore agree to participate in the study.

Name of patient…………………….. ……Signature……………………Date………...

Name of investigator……………………...Signature…………………....Date………....

Name of witness………………………......Signature……………………Date………....

APPENDIX 13 (b): CONSENT FORM FOR SURVEY IN LUGANDA

Study title: Improving stroke prevention and outcomes in Uganda: Population survey and hospital based study

Akulira okunoonyereza: Nakibuuka Jane, asangibwa mu Department of Medicine, School of Medicine, Makerere University College of Health Sciences ku ssimu: 0772618111

To be read by/read out to potential participants ***aged 18 years or older***

Nga tonaba kuteeka mukono ku kiwandiiko kino, ssoma era olowooze ku bwino ono akuweebwa akwata ku nsonga lwaki omusomo gukolebwa, emitendera gyagwo, ebiguganyurwamu ne bwino omulala omukulu. Tukusaba obuuze oyo muntu abuuza ebibuuzo bwekiba ng'omo ku bwino akuweebwa tategerekeka bulungi.

1. Lwaki omusomo guno gukolebwa?

Ebintu ebiteeka omuntu mu kabegye k'okufuna obulwadde bwa situlooko nga obulwadde bwa puleesa n'obwasukaali biropeddwa ng'ebyo ebitera okulabibwa era nga byebivaako okufuna obulwadde bwa situlooko. Obulwadde bwa situlooko butandise okulabibwa ennyo era nga bukulu mu kuleetawo ebiva mu kulwala ebibi ng'okufa n'okulemala mu nsi zimufuna mpola nga Uganda. Obungi/omugugu gwa situlooko n'ebintu ebiteeka abantu mukabegye k'okumufuna tebinasomwako bulungi mu kitundu kyaffe, songa kyetaagisa mu kutegeka empereza ez'obyobulamu. Omuntu wabuligyo kyategeera oba kyamanyi ku bulwadde bwa situlooko n'ebiteeka omuntu mu kabegye k'okumufuna era nebyebakola singa obulwadde bwa situlooko buzze byamugaso nyo mu kuziyizaamu obulwadde bwa situlooko n'ebimuvaamu ebibi ng'okufa n'okulemala. Tutegeka okukola omusomo okulaba obungi n'ebyo ebiteeka abantu mu kabegye k'okufuna obulwadde bwa situlooko n'abiki abantu mu bitundu gyebali byebamanyi era byebategeera ku bulwadde bwa situlooko. kisobozese okutegeeza beekikwatako nga gavumenti ku kukola entegeka ezisingako obulungi mu by'empereza ez'ebyobulamu okusobola okuziyiza n'okujjanjaba endwadde.

2. Lwaki nondeddwa okwetaba mu musomo guno?

Obeera mu kibuga oba mukyalo ekisangibwa mu disitulikiti y'eWakiso mu kiseera omusomo wegukolebwa era ng'enyumbayo erondedwamu. Tujja kusaba abaami n'abakyala enkumi ssatu mu lunaana mu babiri (3802) okwetaba mu musomo guno. Bweguba nga gwe mulundigwo ogusooka okwetaba mu musomo nga guno, kozesa omukisa guno okutegeera ebikwata ku musomo era obuuze ebibuuzo byonna byoyinza okuba nabyo

3. Kiki ekinambaako singa nsalawo okwetaba mu musomo?

Bwosalawo okwetaba mu musomo, ojja kuba osuubirwa okuddamu ebibuuzo ebikwata ku ndwadde zo zolina kati n'ezemabega ezekuusa ku bulwadde bwa situlooko n'ebyo ebiteeka omuntu mu kabegye k'okumufuna okugeza singa wafunako obulwadde bwa situlooko emabega, bwoba ng'olina obulwadde bwa puleesa, bwoba ng'onywa sigala, okola ekisasayizi n'ebirala. Era ojja kukeberebwa era tukupime puleesa yo ey'omusaayi, obuzitobwo, n'obuwanvu bwo nga bino bimu kwebyo ebinakolebwa. Tujja kukusaba oleme kulya

okumala essaawa munaana okuyita mukiro tusobole okufuna ogumu ku musaayi/sampo y'omusaayi okuva ku musuwa gw'okumukono enkeera. Kino kijja kukolebwa mu ngeri entuufu nga tukozesa empiso n'ebomba empya okutali buwuka. Omusaayi gwetukungaanya gujja kuteekebwa mu kaccupa omuteekwa sampo era gusindikibwe mu laabu awakeberebwa omusaayi gukeberebwe. Tujja kukebera okulaba obungi bwa masavu g'omumusaayi, okulaba obungi bwe birungo ebisangibwa mu musaayi mubujuvu bwabyo era ne kabotoogo.

Ebinaava mu kukeberebwa bijja kukuweebwa bwoba ng'oyagala okubimanya era n'abakozi b'omusomo bajja kukunyonyola ebiva mu kukeberebwa kyebitegeeza. Bwotakima biva mu kukeberebwa kwo, twagala tukutuukirire singa ebiva mu kukeberebwa biraga nti waliwo ekitali kituufu/ssibyebyabulijjo. Okusinzira ku binaava mu byakeberebwa, bajja kukuwa amagezi obanga wetaaga okukeberebwa okusingako awo era bakubuulire n'enkyuukakyuuka zolina okukola mu ngeri gyokwatamu obulamubwo obwabulijjo oba obujjanjabi okukuyamba gwe ne famileyo okwewalamu obulwadde bwa situlooko n'edwadde ezibateeka ku kabegye akawagulu okufuna obulwadde bwa situlooko.

4. Nyinza okugaana okwetaba mu musomo oba nendekeraawo okwetaba mu musomo?

Kiri gyoli okusalawo okwetaba mu musomo oba nedda. Era osobola okulekeraawo okubeera mu musomo essaawa yonna woyagalira nga towadde nsonga yonna.

5. Buzibu ki bweba nsuubira obuva mukwetaba mu musomo?

Bwewetaba mu musomo guno obuzibu bwoyolekede butono nyo. Omutendera ogw'okujjibwako omusaayi okuva ku misuwa gyo kiyinza okukuleetera obulumi obusaamusaamu n'okutawanyizibwa. Oyinza okufulumya omusaayi omutono okuva mukifo ekyo, naye ng'omusaayi ogunafunibwa gujja kuba mutononyo okubaako akabegye gweguteeka ku bulamubwo. Bwewetaba mu musomo guno, oyinza okufiirwa ebimu ku byamabyo, naye nze nga anoonyereza, nkukakasa nti likodizo/ebiwandiikobyo eby'ekisawo bijja kukuumibwa nga byakyama. Tujja kukozesa ennamba enekusifu/koodi okusobola okukumanya era nga likodizo/ebiwandiikobyo bijja kuterekebwa awantu awasibwa n'ekisumuluzo. Ebikumanyisa tebija kukozesebwa mu lipoota zonna oba ebitabo ebinakubibwa nga biva mu musomo.

6. Waliwo byeganyurwa mu kwetaba mu musomo?

Ojja kubuuzibwa ebibuuzo, wekebejjebwe era obe n'okeberebwa ku bintu ebiteeka abantu mu kabegye k'okufuna obulwadde bwa situlooko n'ensonga/ebintu ebimanyidwa ebivaako ebiva mu kulwala ebibi ng'omaze okufuna situlooko awatali kufulumya ssente zonna. Ng'ebiva mu kukeberebwa bikomezebwawo gyoli, ojja kufuna okuweebwa amagezi ku ngeri ey'okweyisaamu mu mbeerazo ez'obulamu obwabulijjo n'enzijjanjabi ebijja okwongera okulongoosa mu mbeera z'obulamu bwo. Bwino anafunibwa okuva kugwe n'abeetabye abalala ajja kuyamba abanoonyereza okumatiza gavumenti okuteekawo empereza ezisingako obulungi mu by'obulamu okusobola okuziyiza n'okujjanjaba obulwadde bwa situlooko.

7. Aani gwenyiza okubuuza singa nina ebibuuzo ebikwata ku musomo?
Abali ku ttimu y'omusomo baagala okukuddamu ebibuuzo byonna byoyinza okuba nabyo essaawa yonna ebikwata ku musomo guno ogw'okunoonyereza. Bwoba n'ekyolina kyonna essaawa eno, tukusaba obuuze. Naye bwoba n'ebibuuzo gyebujja mumaaso oyinza okuntuukirira mu dipatimenti/kitongole kyabasawo eky'edwaliro ly'emulago (Department of medicine, Mulago hospital) ku ssimu: 0772618111 oba akulira okunoonyereza ku ndwadde z'omutima Dr. Mondo Charles, ku Uganda Heart Institute, Mulago Hospital awajjanjabirwa omutima ku ssimu: +256-774460496

Ku bikwata ku kyewebuuza ku ddembelyo mu musomo guno, tukusaba otuukirire ssentebe wa kakiiko ak'ettendekero ly'ekisawo eMakerere akavunanyizibwa okulaba nti eddembe ly'abanoonyerezebwako terityoboolwa mu misomo egikolebwa akayitibwa Makerere School of Medicine Research and Ethics Committee ku ssimu: (+256) 0414-533541

8. Sitaatimenti ey'obweyamwo
Ekika n'ekigendererwa ky'omusomo guno binyinyonyodwa gyendi era bitegede bulungi. Era kati nzikiriza okwetaba mu musomo.

Erinya ly'onoonyerezebwako…………………Omukono…………Ennaku z'omwezi …...

Erinya ly'anoonyereza……………………..Omukono……………Ennakuz'omwezi …….

Erinya ly'omujjulizi……………………Omukono……………….Ennaku z'omwezi…......

APPENDIX 14: THE INTERNATIONAL PHYSICAL ACTIVITY (IPA) SCORE[156]

Sporting activity in last 3 months<br><br>• Rarely<br>• 1-2 times/ week<br>• ≥ 3 times / week
Sporting activity causes perspiration and breathlessness
Physical activity that causes perspiration and breathlessness<br><br>• Rarely<br>• 1-2 times/ week<br>• ≥3 times/ week
Means of transport<br><br>• Walking<br>• Bicycle<br>• Taxi<br>• Drive self<br>• Motor cycle
Distance walk in a day<br><br>• < 100m<br>• 100m- ½ KM<br>• > ½ Km- 1KM

APPENDIX 15: CAGE ALCOHOL DEPENDENCY GRADING SYSTEM [157]

The CAGE will be used to determine suspected alcohol problem or abuse/dependency
.
Have you ever felt you ought to **c**ut down on your drinking?

Have people ever **a**nnoyed you by criticizing your drinking?

Have you felt **g**uilty or bad about your drinking?

Have you **e**ver had a drink first thing in the morning to steady your nerves?

Interpretation:
1) No suspected alcohol problem (all no)

2) Suspected alcohol problem (one yes)

3) Alcohol abuse/ dependency (more than one yes)

APPENDIX 16: DIAGNOSTIC CRITERIA FOR CEREBRAL INFARCTION

Trial of ORG 10172 in Acute Stroke Treatment[160]

1. Cerebral infarction:

A) Lacunar stroke:

- CT scan showed a low-density lesion <2cm in maximum length that conformed to a vascular territory compatible with the clinical presentation

B) Cardioembolic stroke:

- Cerebral infarct with a recognised source for emboli or systemic emboli originating from the heart and no lacune by CT compatible with the clinical presentation. Sources for cardioemboli included atrial fibrillation, endocarditis, mitral valve disease, clot in the heart by echocardiogram, recent cardiac surgery or trauma, or myocardial infarction.
- Hemorrhagic infarction (mottled) by CT scan

C) Atherosclerotic stroke

- Focal infarct in the setting of evidence for large-vessel disease, consisting of preceding TIAs in the same vascular territory or carotid artery bruit over the proximate artery or internal carotid occlusion or severe stenosis (> 70%) by carotid duplex sonography at the carotid bifurcation if compatible, with no evidence of lacunar, mottled infarction, or small cortical infarct by CT and no sources of emboli.

D) Unspecified stroke

- All cases not classified by the above rules for lacunar, embolic, or atherosclerotic infarction.

APPENDIX 17: PROCEDURE FOR MEASURING BLOOD PRESSURE

The study participants will be requested before hand to refrain from smoking and drinking alcohol or caffeinated beverage, eating, drinking (except water) and taking drugs that affect the BP, half an hour before the measurement of BP. Blood pressure will be measured with an Omron M 6 automatic BP monitor whose accuracy has been clinically validated[168].

Survey participants will be allowed to sit up right with legs uncrossed. They will be requested to roll up the sleeves of the shirts or blouses so that the upper left arm is bare. This will be rested on a table with the ante-cubital fossa at the level of the lower sternum. After sitting for five minutes, measurements will be taken. We will ensure adequate cuff size if the the cuff with the bladder over the brachial artery encircles and covers 2/3 of length of arm, with its lower border at least 1 inch (2-3 cm) above the ante-cubital space (arm cuffs 9-13 inches and 13-17 inches will be used). The bladder will be deflated slowly to record exact values. Systolic pressure will be determined by the first heard sound (Korotkoff phase I). Diastolic pressure will be recorded at the level when the sound just disappears (Korotkoff phase V).Three measurements will be taken 3 minutes apart. The average values of the closest two readings will be obtained.

Participants in the hospital study will have the BP measured while in the lying position and an average of two readings taken five minutes apart will be taken. The value of the BP obtained will be described according to the National Clinical Guideline Centre (NCGC) clinical guideline 127 classification[158]. (Appendix 24)

APPENDIX 18: NATIONAL CLINICAL GUIDELINE 127 [158]

Classification of blood pressure levels (Millimeters of mercury)

Systolic blood pressure (mmHg)	Diastolic blood pressure (mmHg)	Classification
<130	<85	Normal
130-139	85-89	High normal
140-159	90-99	Hypertension (Stage 1)
160-179	100-109	Moderate hypertension (Stage 2)
>180	>110	Severe hypertension (Stage 3)

APPENDIX 19: PROCEDURES FOR ARTHROPOMETRIC MEASUREMENTS

The Body Mass Index (BMI) and the Waist-hip ratio (WR) will be described according to the European food information council[169]

Survey participants will have their height (m) and weight (Kg) measured in order to calculate the BMI

Height: This will be described as the perpendicular distance between the top of the head (the vertex) and the bottom of the feet. It will be measured with a SECA 214 portable stadiometer. The subjects will be requested to stand on the centre of the base with their back to the stadiometer. The subjects will be barefoot, standing straight with their arms hanging by their side and the back of the head, back calves and heels touching the upright. They will then be requested to put their feet together and move back until their heels touch the bottom of the stadiometer upright. Their buttocks and upper part of their back will also be touching the stadiometer upright. The subject's head will be in the Frankfort plane. This will be achieved by making sure that the lower edge of the eye socket (the Orbitale) is horizontal with the Tragion (Figure 4). The vertex will be the highest point on their head. If their head is not aligned properly, the subject will be requested to raise or lower their chin until it is in the Frankfort Plane. The subject will then be asked to take a deep breath and hold it and the headboard until it is in contact with the head. The reading will then be taken the nearest 0.1 cm and recorded.

Figure 7 demonstrating the Frankfort plane

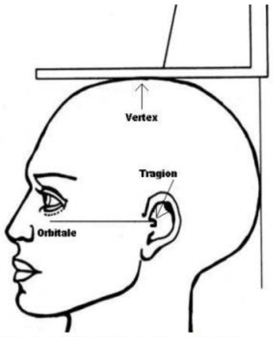

Source: Adapted from the ISAK Manual, 2001.

Weight: A SECA 762 weighing scale will be used to measure weight. The scale will be placed on a hard surface and they will be turned on. Once set to zero, the subject, who will be putting on loose clothing, will be asked to stand on the centre of the scales without support, with their arms loosely by their sides, head facing forward and their feet 12-15 cm apart so

that their weight would be equally distributed on each leg. The reading will be taken to the nearest 0.1 kg.

BODY MASS INDEX
<18.5 Underweight
18.5-24.9 Healthy weight
25-29.9 Overweight
≥30 Obese

Participants will have their waist and hip circumference measured in order to calculate the Waist-hip ratio.

Waist circumference: This will be defined as thecircumference of the abdomen at its narrowest point between the lower costal (10th rib) border and the top of the iliac crest, perpendicular to the long axis of the trunk. We shall use an anthropometric measuring tape. The cross-hand technique will be used to measure the waist girth. This will assist to minimize gaps between the tape and the body surface, and to minimise indentations of the body surface wherever possible. The subjects will be requested to stand upright in a relaxed manner with their arms slightly away from the sides, feet comfortably apart at about 15 cm, weight evenly balanced on both feet. This measurement will then be taken at the level of the midpoint between the inferior margin of the last rib and the iliac crest in the mid-axillary plane. The landmarks will be located by palpation and marked and then the midpoint found using a tape measure. Measurements will be to the nearest 0.1cm after normal expiration.

Hip circumference: This will also be measured to the nearest centimetre using an unstretchable tape measure at the level of the anterior superior iliac spine.Waist to hip ratio will be obtained by dividing waist by hip circumference. The value obtained will be used to describe abdominal obesity according to the Adult treatment panel-ATP III (ref)

Waist-hip ratio= Waist circumference

Hip circumference

Waist circumference, >102cm for men and >88cm for women

Waist-hip ratio, >0.90 for men and >0.85 for women

APPENDIX 20: SCANDINAVIAN STROKE SCALE [159]

Patient Name: ____________________________

Rater Name: ____________________________

Date: ____________________________

Function	Score	Prognostic Score	Long Term Score
**Consciousness**:			
-fully conscious	**6**	____	
-somnolent, can be awaked to full consciousness	**4**		
-reacts to verbal command, but is not fully conscious	**2**		
**Eye movement**:			
-no gaze palsy	**4**	____	
-gaze palsy present	**2**		
-conjugate eye deviation	**0**		
**Arm, motor power *:**			
-raises arm with normal strength	**6**	____	____
-raises arm with reduced strength	**5**		
-raises arm with flexion in elbow	**4**		
-can move, but not against gravity	**2**		
-paralysis	**0**		
**Hand, motor power *:**			
-normal strength	**6**	____	
-reduced strength in full range	**4**		
-some movement, fingertips do not reach palm	**2**		
-paralysis	**0**		
**Leg, motor power *:**			
-normal strength	**6**	____	____
-raises straight leg with reduced strength	**5**		
-raises leg with flexion of knee	**4**		
-can move, but not against gravity	**2**		
-paralysis	**0**		

Orientation:

-correct for time, place and person	**6 ____**
-two of these	**4**
-one of these	**2**
-completely disorientated	**0**

Speech:

-no aphasia	**10 ____**
-limited vocabulary or incoherent speech	**6**
-more than yes/no, but not longer sentences	**3**
-only yes/no or less	**0**

Facial palsy:

-none/dubious	**2 ____**
-present	**0**

Gait:

-walks 5 m without aids	**12 ____**
-walks with aids	**9**
-walks with help of another person	**6**
-sits without support	**3**
-bedridden/wheelchair	**0**

**Maximal Score ____**	**22**	**48**

* Motor power is assessed only on the affected side.

APPENDIX 21: MODIFIED RANKIN SCALE [162]

Patient Name: ___________________________

Rater Name: ___________________________

Date: ___________________________

Score Description

0 No symptoms at all

1 No significant disability despite symptoms; able to carry out all usual duties and activities

2 Slight disability; unable to carry out all previous activities, but able to look after own affairs without assistance

3 Moderate disability; requiring some help, but able to walk without assistance

4 Moderately severe disability; unable to walk without assistance and unable to attend to own bodily needs without assistance

5 Severe disability; bedridden, incontinent and requiring constant nursing care and attention

6 Dead

TOTAL (0–6): _______

APPENDIX 22: THE BARTHEL INDEX [163]

Patient Name: ___________________________

Rater Name: ___________________________

Date: ___________________________

Activity Score

FEEDING
0 = unable
5 = needs help cutting, spreading butter, etc., or requires modified diet
10 = independent ______

BATHING
0 = dependent
5 = independent (or in shower) ______

GROOMING
0 = needs to help with personal care
5 = independent face/hair/teeth/shaving (implements provided) ______

DRESSING
0 = dependent
5 = needs help but can do about half unaided
10 = independent (including buttons, zips, laces, etc.) ______

BOWELS
0 = incontinent (or needs to be given enemas)
5 = occasional accident
10 = continent ______

BLADDER
0 = incontinent, or catheterized and unable to manage alone
5 = occasional accident
10 = continent ______

TOILET USE
0 = dependent
5 = needs some help, but can do something alone
10 = independent (on and off, dressing, wiping) ______

TRANSFERS (BED TO CHAIR AND BACK)
0 = unable, no sitting balance
5 = major help (one or two people, physical), can sit
10 = minor help (verbal or physical)
15 = independent ______

MOBILITY (ON LEVEL SURFACES)
0 = immobile or < 50 yards
5 = wheelchair independent, including corners, > 50 yards
10 = walks with help of one person (verbal or physical) > 50 yards
15 = independent (but may use any aid; for example, stick) > 50 yards ______

STAIRS
0 = unable
5 = needs help (verbal, physical, carrying aid)
10 = independent ______

TOTAL (0–100): ______

The Barthel ADL Index: Guidelines
1. The index should be used as a record of what a patient does, not as a record of what a patient could do.

2. The main aim is to establish degree of independence from any help, physical or verbal, however minor and for whatever reason.

3. The need for supervision renders the patient not independent.

4. A patient's performance should be established using the best available evidence. Asking the patient,friends/relatives and nurses are the usual sources, but direct observation and common sense are also important. However direct testing is not needed.

5. Usually the patient's performance over the preceding 24-48 hours is important, but occasionally longer periods will be relevant.

6. Middle categories imply that the patient supplies over 50 per cent of the effort.

6. Use of aids to be independent is allowed.

APPENDIX 23: ADVERSE EVENT REPORTING FORM [170]

<table>
<tr><td>REC Protocol #:</td><td colspan="2"></td><td>PI Institution:</td></tr>
<tr><td>Principal investigator:</td><td colspan="2"></td><td>Phone:
Email:</td></tr>
<tr><td>Report prepared by:</td><td colspan="2"></td><td>Phone:
Email:</td></tr>
<tr><td colspan="4">Study title:</td></tr>
<tr><td colspan="4">Study sponsor:</td></tr>
<tr><td>Date of adverse event</td><td colspan="2">Subject’s initials or study #</td><td>Type of report
( ) Initial ( ) Follow up</td></tr>
<tr><td colspan="4">Brief description of adverse event(including diagnosis)</td></tr>
<tr><td colspan="2">Location of adverse event: ……………………….
Research involves a ( ) Drug
( ) Device
( ) Procedure

Name of Drug, Device, Procedure:</td><td colspan="2">Adverse event appears to be
( ) Not related ( ) Unlikely
( ) Possible related ( ) Related
( ) Probably related ( ) Unknown
Expectedness ( ) Expected
( ) Not expected
Severity of adverse event: ( ) Mild
( ) Moderate ( ) Severe ( ) Fatal</td></tr>
<tr><td colspan="2">Is the drug, device investigational? ( )Yes
( ) No

Has the adverse event been reported to:
( ) Sponsor, date of report ……………………………
( ) REC, date of report………………………………..</td><td colspan="2">Outcome of adverse event:
( ) Death (due to event)
( ) Death (due to other causes)
( ) Hospitalisation
( ) Extended hospitalisation
( ) Congenital abnormality
( ) Recovered ( ) Not yet recovered
Recovery of subject: ( ) Complete
( ) Moderate ( ) Minimal ( ) None
( ) Not yet resolved ( ) Unknown</td></tr>
<tr><td colspan="2">Was this adverse event addressed in the protocol and consent form
Was this adverse event addressed in the investigators brochure
Are changes required to the protocol?
Are changes required to the consent form?

If changes are required, please attach a copy of the revised protocol/consent form with changes highlighted with a bright coloured high lighter

If changes are not required, please explain as to why changes to the protocol/consent form are not necessarily based on the event</td><td colspan="2">Yes No Not applicable

Yes No Not applicable

Yes No Not applicable
Yes No Not applicable</td></tr>
<tr><td colspan="4">From the data obtained or from currently available information, do you see any need to reassess the risks and benefits to the subjects in this research ( ) Yes ( ) No</td></tr>
<tr><td colspan="4">P.I. Signature</td></tr>
</table>

Complete entire form, do not leave any blanks
